# Systematic measurement of combination-drug landscapes to predict *in vivo* treatment outcomes for tuberculosis

**DOI:** 10.1016/j.cels.2021.08.004

**Published:** 2021-11-17

**Authors:** Jonah Larkins-Ford, Talia Greenstein, Nhi Van, Yonatan N. Degefu, Michaela C. Olson, Artem Sokolov, Bree B. Aldridge

**Affiliations:** 1 Department of Molecular Biology and Microbiology, Tufts University School of Medicine, Boston, MA 02111, USA; 2 Stuart B. Levy Center for Integrated Management of Antimicrobial Resistance, Boston, MA 02111, USA; 3 Graduate School of Biomedical Sciences, Tufts University School of Medicine, Boston, MA 02111, USA; 4 Laboratory of Systems Pharmacology, Harvard Program in Therapeutic Science, Harvard Medical School, Boston, MA 02115, USA; 5 Department of Biomedical Engineering, Tufts University School of Engineering, Medford, MA 02155, USA

**Keywords:** tuberculosis, antibiotics, drug combinations, combination therapy, drug interactions, mycobacteria, infectious diseases

## Abstract

Lengthy multidrug chemotherapy is required to achieve a durable cure in tuberculosis. However, we lack well-validated, high-throughput *in vitro* models that predict animal outcomes. Here, we provide an extensible approach to rationally prioritize combination therapies for testing in *in vivo* mouse models of tuberculosis. We systematically measured *Mycobacterium tuberculosis* response to all two- and three-drug combinations among ten antibiotics in eight conditions that reproduce lesion microenvironments, resulting in >500,000 measurements. Using these *in vitro* data, we developed classifiers predictive of multidrug treatment outcome in a mouse model of disease relapse and identified ensembles of *in vitro* models that best describe *in vivo* treatment outcomes. We identified signatures of potencies and drug interactions in specific *in vitro* models that distinguish whether drug combinations are better than the standard of care in two important preclinical mouse models. Our framework is generalizable to other difficult-to-treat diseases requiring combination therapies. A record of this paper’s transparent peer review process is included in the supplemental information.

## Highlights

• Comprehensive dataset of TB drug combination responses in multiple *in vitro* models
• Computational modeling predicts mouse treatment outcome based on *in vitro* data
• Ensembles of *in vitro* models predict treatment outcomes in in *vivo* environments
• *In vitro* drug combination potencies predict outcomes in a relapsing mouse model

## Graphical abstract

**Figure:**
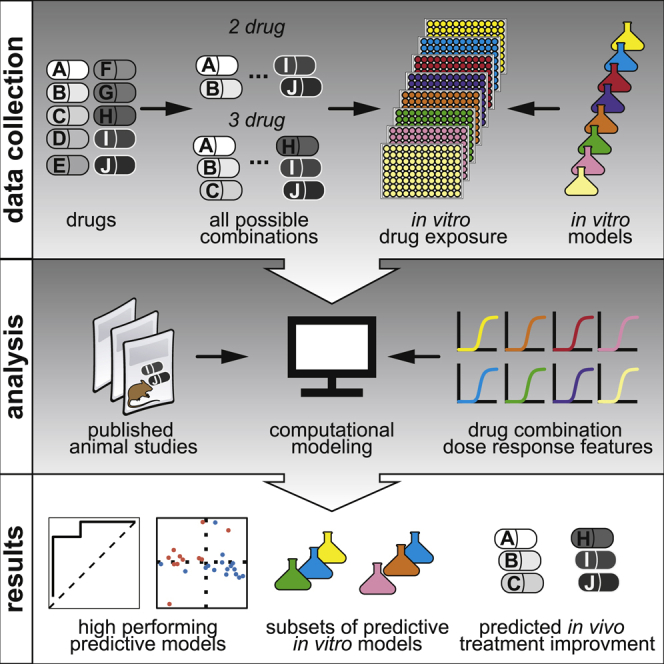

## Teaser

This study establishes a framework to prioritize combination antibiotic therapies for tuberculosis by combining *in vitro* dose response measurement and *in vivo* treatment outcomes using mathematical modeling. The experimental and computational tools developed in the study identified sets of *in vitro* conditions with highly predictive information and signatures of potency and drug interaction that distinguish drug combinations with improved *in vivo* treatment outcomes.

## Introduction

Tuberculosis (TB), caused by infection with *Mycobacterium tuberculosis* (Mtb), remains a major global health issue. In 2019, an estimated ten million people fell ill with TB, and about 1.4 million people died (World Health Organization, 2020). Development of shorter treatment regimens is a key part of the third pillar of the WHO End TB Strategy (World Health Organization, 2014). Multidrug treatment regimens were developed to treat active TB infections by shortening treatment duration, reducing disease relapse, and decreasing antibiotic resistance development (Fox et al., 1999). The standard TB treatment is six to nine months of multidrug treatment with an estimated 85% cure rate (World Health Organization, 2020; Kerantzas and Jacobs, 2017; Tiberi et al., 2018a). The first two months of treatment (intensive, bactericidal phase) consist of four drugs (isoniazid, rifampicin, pyrazinamide, and ethambutol) that reduce sputum Mtb levels but are less effective against non-replicative bacilli (Mitchison, 1996; Kerantzas and Jacobs, 2017; Fox et al., 1999). The following four to seven months of treatment (continuation phase) consist of two drugs (isoniazid and rifampicin) aimed at reducing disease relapse by treating persisting bacteria that survived the intensive phase (Fox et al., 1999; Kerantzas and Jacobs, 2017; Mitchison, 1996). New regimens that can more efficiently treat Mtb are needed to shorten the intensive phase of treatment and reduce or eliminate the bacteria that persist and require continuation phase treatment (Kerantzas and Jacobs, 2017).

**Figure 1:**
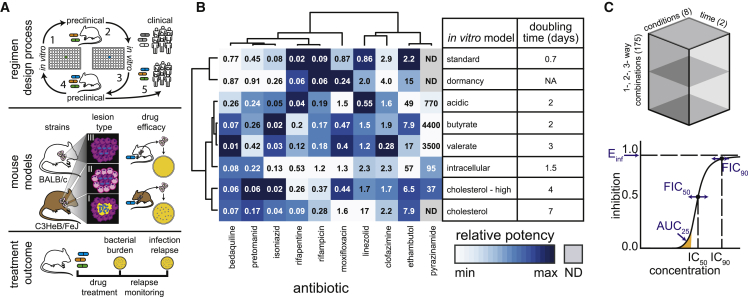
TB drug development pipeline and a ten-drug DiaMOND compendium of Mtb response to drug combination treatment (A) (Top) Schematic of TB drug regimen evaluation process highlighting in vitro drug discovery and assessment followed by preclinical (e.g., mouse model) evaluation of drug combinations. A new drug (green) is discovered and evaluated *in vitro* (1) and evaluated in combination with another drug (orange) in preclinical studies (2). Another drug (blue) is selected and evaluated *in vitro* (3) and then *in vivo* in combination with the previously tested combination (4). Superiority of a combination over the standard of care in preclinical models leads to clinical trials (5). (Middle) Diagram highlighting notable differences in disease pathology and drug response in mouse strains (BALB/c and C3HeB/FeJ) used in TB research. (Bottom) Schematic of treatment outcome assessment in mouse studies. Bacterial burden is assessed by monitoring bacterial burden during and immediately following drug treatment. Disease relapse is assessed by monitoring for culturable bacteria after drug treatment cessation. (B) Relative potencies of the ten compendium drugs in eight *in vitro* conditions (IC_90_, terminal time point; left) with doubling times for each condition in untreated Mtb (right). IC_90_ (μg/mL) values are indicated and color scaled (log_10_ transformation) within each drug. Hierarchical clustering of potencies as calculated with cosine distances and average linkage. ND, not determined; NA, not applicable. (C) (Top) Schematic data cube of the DiaMOND compendium. Mtb were treated with all 1-, 2-, and 3-way drug combinations (175 combinations) among 10 drugs in dose responses measured in 10-dose resolution in at least biological duplicate. Dose response measurements were made in eight *in vitro* models and at 3–4 time points, but we focus on 1–2 time points for analysis (Data S1); therefore, this data cube represents ~25% of the total measurements made. (Bottom) Metrics from DiaMOND dose response curves. IC_50_ and IC_90_ are used to calculate drug interactions at the 50% and 90% levels of growth inhibition (FIC_50_ and FIC90, respectively). Three potency metrics are derived: AUC_25_, normalized area under the curve until 25% inhibition; E_inf_, theoretical maximum inhibition, and (not shown); Grinf, theoretical maximum normalized growth rate inhibition (Box 1; STAR Methods).

Due, in large part, to the heterogeneity of TB lesions and treatment response among the Mtb population, combination therapy is required to treat active TB. Therapies should therefore be designed as combinations of antibiotics rather than single antibiotics alone. There are many drug options for new treatment regimens using existing drugs and drugs in development (Evans and Mizrahi, 2018), which creates an enormous number of possible drug combinations (Tiberi et al., 2018a). Despite the size of the combination space, new TB regimens are built by augmenting well-studied drug combinations with the substitution or addition of new drugs (Ginsberg and Spigelman, 2007; Kerantzas and Jacobs, 2017; Tiberi et al., 2018a, 2018b; Wallis et al., 2016; Lienhardt et al., 2019; Dooley et al., 2019), often based on the results of iterative *in vitro* and preclinical studies (Figure 1A). A phase 3 clinical trial (“Study 31”) recently demonstrated that treatment duration could be shortened by substituting two of the drugs in the standard four-drug regimen with existing TB antibiotics (Dorman et al., 2021). Relatively new drugs (bedaquiline, pretomanid, delamanid, and SQ109) that can target non-replicative bacteria in *in vitro* and preclinical studies (Iacobino et al., 2017; Liu et al., 2018; van den Boogaard et al., 2009) are components of new treatment-shortening regimens for multidrug resistant TB (MDR-TB) (Conradie et al., 2020; Pontali et al., 2019; Tiberi et al., 2018a). The treatment-shortening potential in phase 2b trials (Diacon et al., 2012; Dawson et al., 2015) led to the phase 3 STAND clinical trial to test the use of pretomanid with moxifloxacin and pyrazinamide (PaMZ, Table 1) (ClinicalTrials.gov, number NCT02342886). Augmentation of the PaMZ combination with the addition of bedaquiline (BPaMZ, Table 1) in a phase 2b trial (Tweed et al., 2019) shortened culture conversion time of MDR-TB so dramatically that the STAND trial was put on permanent hold to start the phase 3 SimpliciTB trial to evaluate BPaMZ for treating both drug-sensitive TB and MDR-TB (ClinicalTrials.gov, number NCT03338621). Using an iterative method of adding or substituting into effective combinations during TB drug regimen design, these studies have demonstrated that there is treatment-shortening potential in the drug combination space. A critical step for developing new treatment regimens is prioritizing the thousands of other drug combinations before clinical testing. However, it is not practical to evaluate thousands of combinations using the current preclinical regimen design process, which combines *in vitro* and small animal studies (Figure 1A). An efficient methodology is needed to systematically assess drug combinations and prioritize the thousands of multidrug combinations for their treatment-shortening potential.

**Table 1:** Abbreviations used in this study

Drugs	
B	bedaquiline, DiaMOND compendium drug, ATP synthesis inhibitor
C	clofazimine, DiaMOND compendium drug, antimycobacterial/multi-process inhibitor
E	ethambutol, DiaMOND compendium drug, cell wall synthesis inhibitor
H	isoniazid, DiaMOND compendium drug, cell wall synthesis inhibitor
L	linezolid, DiaMOND compendium drug, protein synthesis inhibitor
M	moxifloxacin, DiaMOND compendium drug, DNA synthesis inhibitor
Pa	pretomanid, DiaMOND compendium drug, cell wall synthesis inhibitor/ nitric oxide production
Z	pyrazinamide, DiaMOND compendium drug, antimycobacterial/multi-process inhibitor
R	rifampicin, DiaMOND compendium drug, transcriptional inhibitor
P	rifapentine, DiaMOND compendium drug, transcriptional inhibitor
D	delamanid, validation drug, cell wall synthesis inhibitor/ nitric oxide production
Su	sutezolid, validation drug, protein synthesis inhibitor
Sq	SQ109, validation drug, multi-process inhibitor
G	gatifloxacin, validation drug, DNA synthesis inhibitor
Cy	d-cycloserine, validation drug, cell wall synthesis inhibitor
Drug combinations	
PaMZ	bedaquiline + pretomanid + moxifloxacin
BPaMZ	bedaquiline + pretomanid + moxifloxacin + pyrazinamide
HRZE	isoniazid + rifampicin + pyrazinamide + ethambutol - four-drug standard of care
HRZ	isoniazid + rifampicin + pyrazinamide - three-drug standard of care
BPaL	bedaquiline + pretomanid + linezolid
MRZ	moxifloxacin + rifampicin + pyrazinamide
RZ	rifampicin + pyrazinamide
R-CHOP	rituximab + cyclophosphamide + doxorubicin hydrochloride + vincristine sulfate + prednisone – anti-cancer drug combination
Treatment outcome classification	
C0	as good or worse than standard of care (HRZE or HRZ)
C1	better than standard of care
Mouse models	
RMM	relapsing mouse model^a^
BMM	bactericidal mouse model^b^
BHeB	bactericidal outcome in C3HeB/FeJ mouse strain^c^
*In vitro* models	
a	acidic
b	butyrate
c	cholesterol (0.05 mM)
d	dormancy
h	cholesterol-high (0.2 mM)
i	intracellular
s	standard
v	valerate
Data and model metrics	
C	constant time point
T	terminal time point
CT	constant and terminal time point are the same
IC_n_	inhibitory concentration at n % growth inhibition
FIC_n_	fractional inhibitory concentration at n % growth inhibition
AUC_25_	normalized area under the dose response curve to the 25% inhibition point
E_inf_	effect at infinite drug concentration (maximum achievable effect)
GR_inf_	normalized growth inhibition effect at infinite drug concentration (maximum achievable effect)
ROC	receiver operator characteristic
AUC	area under the ROC curve
PR	precision-recall
F1	harmonic mean of the precision and recall

Abbreviations along with brief descriptions are listed.

a The RMM outcome assesses lasting cure months after cessation of drug treatment in the most commonly used mouse strains (e.g., BALB/c, C56BL/6, and Swiss).

b The BMM outcome assesses reduction of bacterial burden immediately following drug treatment in the most commonly used mouse strains.

c The BHeB assesses reduction of bacterial burden immediately following drug treatment but in the pathologically distinct C3HeB/FeJ mouse strain.

Animal models are critical to regimen development, and mouse models are a primary tool in multidrug therapy design (Figure 1A (Dooley et al., 2016; Gumbo et al., 2015; Nuermberger et al., 2008; Tasneen et al., 2011; Nuermberger, 2017)). Mouse strains where Mtb is primarily intracellular (e.g., BALB/c and C57BL/6) are the most widely used (Nuermberger, 2017). Mouse strains that form mixed lesion types (e.g., C3HeB/FeJ) are used to study drug response because the disease pathology is more human-like, include granulomas with caseous necrotic cores (Figure 1A) (Apt and Kramnik, 2009; Gumbo et al., 2015; Kramnik and Beamer, 2016). Mtb drug response differs between these two types of mouse models, and both are important preclinical tools because the model-specific drug response is thought to result from the different lesion microenvironments present in each animal model (Figure 1A), and the differential drug exposure in lesion compartments is influenced by lesion structure (Driver et al., 2012; Lanoix et al., 2015b; Lenaerts et al., 2015; Nuermberger, 2017). Drug treatment efficacies are often evaluated first by directly measuring bacterial burden followed by monitoring disease relapse once treatment is completed (Figure 1A). Enumerating bacterial burden at different times during treatment is an efficient method for assessing drug treatment and is often used to eliminate treatments with moderate effects from further consideration (Tasneen et al., 2016; Li et al., 2015; De Groote et al., 2011; Gumbo et al., 2015; Nuermberger, 2008). In contrast, monitoring relapse is considered a more reliable outcome for assessing the durability of cure and is more comparable with the clinical outcome of treatment success (Gumbo et al., 2015; Nuermberger, 2008; Dooley et al., 2016).

Despite their utility for regimen development, comprehensive drug combination measurements in mice are not feasible. It is only practical to perform systematic drug combination studies *in vitro*, but *in vitro* studies do not clearly map to *in vivo* outcomes (Parish, 2020; Nuermberger, 2017). Many *in vitro* models mimic aspects of the host microenvironment encountered in the different TB lesion types. Some of these *in vitro* models are well suited for systematic drug combination studies, but none have been validated to prioritize drug combinations against preclinical animal models. Mtb drug response is environment specific, underscoring the need to validate *in vitro* models. For example, compared with standard (neutral) glucose-rich growth conditions, pyrazinamide is more effective in acidic *in vitro* conditions (Tarshis and Weed, 1953; McDermott and Tompsett, 1954), whereas bedaquiline is more effective in lipid-rich medium (Koul et al., 2014). Environment-specific drug efficacies are also observed *in vivo*; whereas treating C3HeB/FeJ mice with either pyrazinamide or bedaquiline results in both responding and non-responding populations of Mtb, treatment of BALB/c mice resulted in only responding populations. Drug response differences among mouse strains were attributed to lesion type and microenvironment differences and differential drug exposure in specific lesion compartments (Lanoix et al., 2016b, Lanoix et al., 2016a, 2015b; Irwin et al., 2016;). The dependency of treatment efficacy on growth environment highlights the challenges in simplifying the complex *in vivo* environment into manageable *in vitro* growth conditions.

We propose to realize the potential of drug combinations to improve treatment by developing a workflow to link *in vitro* measurement of drug response to outcomes in mouse models. Our long-term goal is to extensively search the drug combination space empirically using practical *in vitro* measurement to prioritize combinations to be tested in preclinical animal models. Here, we utilized the efficiency of an experimental design and analysis method called DiaMOND (diagonal measurement of n-way drug interactions) (Cokol et al., 2017) to create a compendium of drug combination responses in Mtb using multiple *in vitro* models that were designed to reproduce aspects of the environments encountered in different lesion types. The compendium contains information that can be linearly combined to distinguish drug combinations that outperform the standard of care. Applying machine learning to this comprehensive *in vitro* dataset, we identified signatures of drug potency and interaction that could also predict treatment outcome *in vivo*. Classifiers based on these signatures also enabled us to establish a mapping between *in vitro* models and the different mouse models, which differ in lesion type (microenvironment) and outcome. Overall, our study establishes a logistical path to optimize combination therapies for TB that is consistent with current regimen design strategies and uses systematic measurement in validated *in vitro* growth models and computational modeling.

## Results

### Drug combination compendium construction

We developed an experimental and computational workflow to efficiently prioritize drug combinations early in regimen development based on drug combination measurements from *in vitro* models. Using the DiaMOND methodology (Cokol et al., 2017), we designed a compendium of drug combination measurements to survey informative drug-dose combinations (DiaMOND compendium). To compare *in vitro* data with treatment responses in animal models, our DiaMOND compendium focused on (1) first- and second-line agents, for which there are abundant animal data, and (2) measurements in *in vitro* growth conditions that model environments encountered during infection.

Mtb encounters a diversity of environmental niches during infection that influence response to drug treatment. We aimed to model drug response by aggregating measurements from a suite of *in vitro* models. We focused on modeling factors previously shown to influence Mtb growth and/or drug response, such as different carbon sources and abundance, low pH, low oxygen tension, and the intracellular environment (Gumbo et al., 2015; Parish, 2020; Lee et al., 2013; Early et al., 2016; Pethe et al., 2010; Guerrini et al., 2018; Baker et al., 2019; Vandal et al., 2009; Gold and Nathan, 2017; de Miranda Silva et al., 2019; Drusano et al., 2021). We developed or adapted eight *in vitro* models that were reproducible and scalable for systematic, high-throughput drug combination assays for this study. We varied carbon sources, with an emphasis on cholesterol and fatty acids, to model the lipid-rich environment in TB granulomas, using butyrate, valerate, cholesterol, and higher levels of cholesterol (cholesterol-high) as sole carbon sources. We used 7H9-based medium to compare against the most commonly utilized *in vitro* growth model with glycerol as a carbon source (standard). We also included *in vitro* models that mimic important factors encountered during infection including in low pH (acidic) and in infection of macrophages (with a J774 model, intracellular). Mtb in a non-replicative state are particularly challenging to sterilize (Sarathy et al., 2018). Many models of non-replicative state have been developed for laboratory study that involve single or multiple stresses (Deb et al., 2009; Early et al., 2019; Del Portillo et al., 2018; Parish, 2020; Gold et al., 2015). Based on some of these previously published models, we chose to develop a model that would be amenable to large-scale, multi-well experiments with drug combinations. Our dormancy model is a low-oxygen multi-stress model that induces dormancy using butyrate as a carbon source, sodium nitrate to respire (Cunningham-Bussel et al., 2013a, 2013b; Sohaskey, 2008), and plate seals to limit oxygen (dormancy). The doubling times varied considerably among the models, ranging from 16 h to one week (Figure 1). We scaled the timing of the experiments relative to the doubling time of each model so that drug response measurements would not be biased by changes in growth rate (Table S1).

### Drug combination dose response measurements

For the DiaMOND compendium, we selected ten antibiotics in first- and second-line TB treatment regimens and for which there are abundant *in vivo* (mouse) data. These drugs include cell wall synthesis inhibitors (ethambutol, isoniazid, and pretomanid), rifamycin transcriptional inhibitors (rifampicin and rifapentine), protein synthesis inhibitor (linezolid), inhibitors of energy metabolism and cellular respiration (bedaquiline and clofazimine), DNA replication inhibitor (moxifloxacin), and the antimycobacterial agent pyrazinamide (Table 1). We treated the Mtb Erdman strain carrying an autoluminescent reporter and measured both optical density (OD_600_) and luminescence at multiple time points after drug treatment. We observed a strong dependency in drug potency on *in vitro* model (Figure 1B, inhibitory concentration to achieve 90% inhibition, IC_90_), consistent with the idea that drug efficacy is influenced by bacterial stress (Warner and Mizrahi, 2006). We did not observe remarkable correlations in potency profiles by *in vitro* model. However, hierarchical clustering of drug potencies showed some groupings of drugs consistent with their target cell process (e.g., rifamycin transcriptional inhibitors group together, isoniazid and pretomanid—inhibitors of cell wall synthesis—group together). We also observed clustering of similar *in vitro* models. For example, potency profiles from growth media with short-chain fatty acids butyrate and valerate as the carbon source group together (Figure 1B).

We observed condition-specific drug potencies consistent with previous reports, suggesting that the models we adapted for high-throughput drug response measurements may be predictive of outcomes in animals. For example, the activity of pyrazinamide in acidic and intracellular models and inactivity in the standard model (Figure 1B) was consistent with *in vitro* (Zhang et al., 2012b; Lamont et al., 2020) and animal studies (Lamont and Baughn, 2019; Pires et al., 2015; Rohde et al., 2007). We also observed pyrazinamide activity with lipid carbon sources, which has not been previously reported. As previously described, the rifamycins shared similar potency profiles with higher potency of rifapentine (Figure 1B) (Alfarisi et al., 2017). Bedaquiline was more potent in medium with lipids as the carbon source compared with standard medium with sugars as previously described (Koul et al., 2014). Isoniazid potency was lower in the dormancy model, consistent with its inactivity toward non-replicating bacilli (Xie et al., 2005; Betts et al., 2002; Wayne and Sramek, 1994) and previous studies showing decreased efficacy in the presence of nitrite (Cunningham-Bussel et al., 2013a). The wide range of single-drug responses and consistency with prior studies suggest that the *in vitro* models in this study produce non-redundant drug response data and form a validated set of conditions to model the lesion-specific variation in drug response.

Using these eight *in vitro* models, we constructed a compendium of systematic drug combination measurements by utilizing the DiaMOND method's efficiency (see Box 1). DiaMOND is a geometric optimization of the traditional checkerboard assay of drug-dose combinations. DiaMOND estimates the effect of combining drugs using a fraction of possible drug-dose combinations and focuses on the single drug and equipotent drug combination dose responses (Cokol et al., 2017). We measured all one-, two-, and three-drug combination dose responses (totaling 175 combinations) in at least biological duplicate (Figure 1C), resulting in a compendium of over 51,000 individual dose response curves. We focused our analysis on up to two time points per *in vitro* model to navigate this complex dataset. We chose the last time point (terminal, T) that is relative to the doubling rate (four to five doublings for most models) and at a consistent treatment timepoint (constant, C) across *in vitro* models, ∼seven days post treatment; Figure 1B; Table S1. We also selected the measurement type that best benchmarks against colony-forming units (OD_600_ for all models except intracellular and dormancy models, for which we used luminescence, Figure S1). This selected dataset (Data S1) represents approximately one-quarter of the total number of compendium dose responses.

Box 1 **DiaMOND: A primer**

**Figure:**
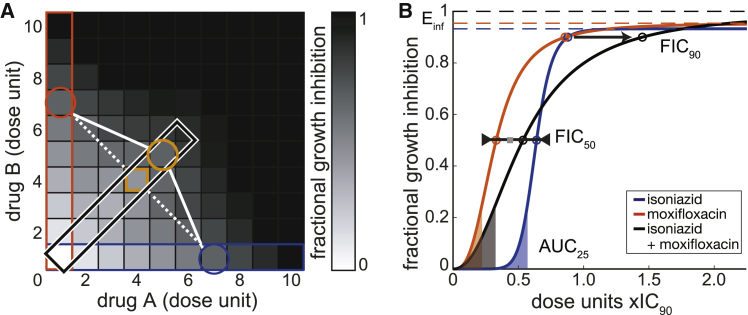
Box 1 Figure. Using DiaMOND to efficiently measure drug combination dose responses

DiaMOND (diagonal measurement of n-way drug interactions) is a quantitative framework to efficiently measure drug interactions. The method is based on geometric sampling of traditional combination checkerboards and can be applied to any number of drugs in combination. Optical density (OD_600_) or luminescence measures are normalized to untreated controls and subtracted from 1 to obtain fractional growth inhibition. The concentrations to achieve a particular effect (e.g., concentration to achieve 90% growth inhibition, IC_90_, depicted in the blue and orange circles of A of the inset figure) are experimentally determined for all single drugs so that dosing in subsequent measurement of drug combinations is equipotent (e.g., the IC_90_ should be dose #~7 for all drugs). Doses may be spaced linearly or logarithmically, but the spacing must be consistent between drugs. The single-drug dose responses (blue and orange boxes) and the equipotent drug combination dose response (black box) are highlighted. Drug interactions can be estimated using only the measurements from these boxes rather than the entire checkerboard by approximating the shape of isoboles (contours of equal effect). In the diagram, the isobole for IC_75_ is traced by the circles. If drug A and B are additive, the isobole would be a straight diagonal, and we calculate the expected IC_75_ on the combination dose response (orange square) where the dotted line intersects with the diagonal (combination) dose response curve. In this illustration, the combination reaches an IC_75_ at higher dose levels (orange circle) than the expected IC_75_, indicating an antagonistic interaction. The ratio of observed and expected doses (observed/expected) is the FIC:

$$FIC=\frac{observed\;combination\;dose}{expected\;combination\;dose}$$

The DiaMOND methodology was used to obtain dose response data for every drug and drug combination measured over multiple time points. A Hill function was fit to these data and several potency and drug interactions metrics were derived from these dose response curves (see B of the inset figure).

DiaMOND dose response metrics:

**E_inf_ (the maximum achievable effect)**: derived from the fitted Hill function (lower pane, dashed lines, colored by single drug or drug combination), Einf describes the maximal achievable effect (upper asymptote, dashed lines) of a given drug or drug combination at a particular time point, where the maximum possible effect is 1.

**AUC**_**25**_: the area under the curve (AUC) simultaneously captures variation in potency and effect of a drug or drug combination, i.e., sensitivity to drug. AUC_25_ captures sensitivity to drug at concentrations with low growth inhibition. To compare low dose potency with other drugs or drug combinations with different concentration ranges, we normalize the area by dividing by the IC_25_. The resulting AUC_25_ values range from 0 (no effect) and 1 (potent).

**FIC**: drug interactions measure the effect of combining drugs on drug potency, i.e., the dose required to achieve a specific effect. The FIC) is the ratio of the observed combination dose (black circle) to achieve X effect over the expected combination dose (gray square), where FIC < 1 indicates synergy, FIC > 1 indicates antagonism, and FIC = 1 indicates additivity. In this example, the FIC_50_ is approximately additive whereas the FIC_90_ is antagonistic, which is indicated by the relative position of the combination dose response (black) near (IC_50_) and to the right (IC_90_) of the single dose response curves. We log transform FICS to balance such that log_2_FIC < 0 is synergistic and log_2_FIC > 0 is antagonistic.

**GRinf**: derived from the growth rate curve (not shown here, see Hafner et al., 2016 for details), GRinf describes the maximal achievable effect of a drug or drug combination on the normalized growth rate, ranging between 1 and −1, where GR(c) is between 0 and 1 in the case of partial growth inhibition, GR(c) = 0 in the case of complete cytostasis, and GR < 0 indicates cell death. This unitless metric describes the effect of a drug on cells independent of doubling time, enabling comparison of drug effect on cells in different growth conditions.

We analyzed the single- and combination-drug treatments to derive potency and drug interaction information (see Box 1). With DiaMOND, we can quantify the degree and directionality of interactions at different growth inhibition levels using common null models (e.g., Loewe additivity and Bliss independence). Drug combinations that are more or less effective than expected based on single-drug behaviors are considered synergistic and antagonistic, respectively. Drug interactions are quantified with fractional inhibitory concentrations (FICs) at different growth inhibition levels (e.g., FIC_50_ and FIC_90_ are measured at the IC_50_ and IC_90_, respectively). FIC measurements were log-transformed to represent synergistic and antagonistic combinations with negative and positive log_2_(FIC) values, respectively. Drug interaction metrics based on Loewe additivity and Bliss independence were correlated (FIC_50_ and FIC_90_ for the constant and terminal time points, r = 0.81, p = 1.0 × 10^−4^, Pearson’s correlation, using permutation analysis Figure S2). In our analysis, we proceeded with Loewe additivity as the null model in our drug interaction (FIC_50_ and FIC_90_) calculations because we have previously validated additivity measures using sham combinations using Loewe additivity (Cokol et al., 2017). Dose response curves provide treatment potency metrics at a low dose (AUC_25_; a normalized area under the curve to IC_25_, see Box 1) or high dose (E_inf_; the maximum achievable effect). To compare potency across models where Mtb have different growth properties, we calculated the maximum achievable inhibition of normalized growth rate (GR_inf_; see Box 1), which allows direct comparison of treatment effects on cells with very different growth rates (Hafner et al., 2016). Though many other drug response metrics may be calculated from DiaMOND data, our analysis focused on these five metrics—FIC_50_, FIC_90_, AUC_25_, E_inf_, GR_inf_—because they represent well-characterized and biologically interpretable aspects of drug interactions and potencies across low- and high-dose ranges.

### Drug synergy is uncommon and does not distinguish effective combinations

**Figure 2:**
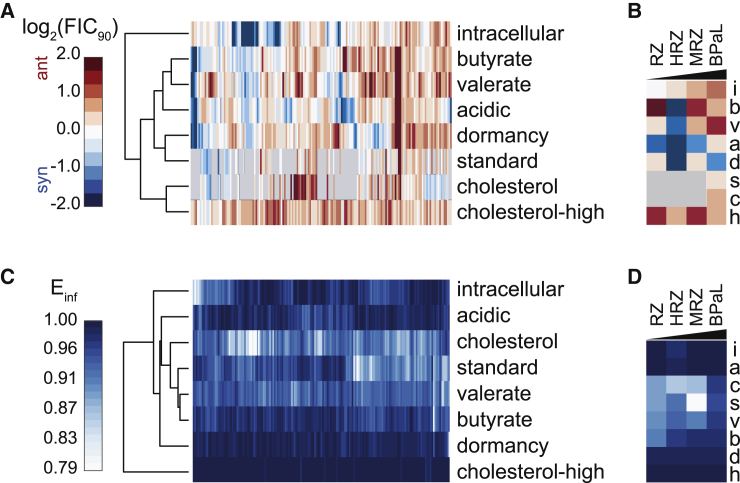
Drug interaction and potency patterns in the DiaMOND compendium (A) Drug interaction profiles of all two- and three-drug combinations among the ten compendium drugs across the *in vitro* models (log_2_(FIC_90_) at the terminal time point, hierarchically clustered based on cosine distance and average linkage). (B) Drug interaction profiles of selected drug combinations ordered by mouse relapse outcome efficacy (Tasneen et al., 2016; Xu et al., 2019; De Groote et al., 2011, 2012; Nuermberger et al., 2004a, 2004b, 2008; Rosenthal et al., 2007; Mourik et al., 2017). See Table 1 for drug combination abbreviations. (C) Drug combination potency profiles of all two- and three-drug combinations among the ten compendium drugs across the *in vitro* models (E_inf_ at the terminal time point, clustered based on cosine distance). (D) Drug interaction profiles of selected drug combinations ordered by mouse relapse outcome efficacy (Tasneen et al., 2016; Xu et al., 2019; De Groote et al., 2011, 2012; Nuermberger et al., 2004a, 2004b, 2008; Rosenthal et al., 2007; Mourik et al., 2017). See Table 1 for drug combination abbreviations. Gray, ND.

To identify patterns in drug interactions, we clustered the compendium drug interactions at the terminal time point in all eight growth environments, using 90% growth inhibition (log_2_(FIC_90_), Figure 2A) and 50% growth inhibition (log_2_(FIC_50_), Figure S3A). Clustering did not reveal obvious model-wide synergy for any combination. Instead, we observed that most drug interactions were antagonistic (70% of FIC_90_>0), consistent with a general trend toward antagonism in drug interactions observed in other organisms (Yeh et al., 2006; Cokol et al., 2011; Chandrasekaran et al., 2016; Mason et al., 2017; Cokol et al., 2017, 2018) and cancer (Richards et al., 2020). The tendency toward antagonism depended on the growth model, with some conditions showing a balance between synergy and antagonism (intracellular and acidic) and all other conditions showing statistically significant antagonism (one-sample t test, mu = 0, p < 0.05, adjusted for multiple hypothesis testing; Figure S3B). Our findings are consistent with those from other organisms and adds to a growing body of literature that suggests synergy is a property of both drug and growth environment rather than an intrinsic property of drugs in combination (Belanger et al., 2020; Sanders et al., 2018; Dillon et al., 2019; Cokol et al., 2018).

To understand whether combinations that tend toward *in vitro* synergy are more effective *in vivo*, we compared selected combinations with differences in disease relapse from the most commonly used mouse strains (e.g., BALB/c, C56BL/6, and Swiss). The relapsing mouse models (RMMs) evaluate drug efficacy months after cessation of drug treatment, somewhat analogous to the clinical measurement of relapse (Figure 1A) (Lanoix et al., 2016; Mitchison and Davies, 2008). We did not observe combination rank ordering by synergy in any growth condition that matched efficacy in the RMM; e.g., BPaL > MRZ > HRZ > RZ (Figure 2B) (Tasneen et al., 2016; Xu et al., 2019; De Groote et al., 2011; Nuermberger et al., 2004a, 2004b, 2008; Rosenthal et al., 2007; De Groote et al., 2012; Mourik et al., 2017). Instead, we observed that the three-drug standard of care (HRZ) was the most synergistic drug combination, and BPaL was the most antagonistic among this subset (Figure 2B). To understand whether the drug interactions in high-order (three or more drugs) combinations were due to lower-order interactions (for example, by pairwise synergies among the three component pairs of a three-way combination) or emergent properties from the high-order combination itself (Cokol et al., 2017; Beppler et al., 2016; Wood et al., 2012), we evaluated the contributions of lower-order and emergent interactions on the total interaction metrics (FICs, as shown in Figures 2A and 2B). The patterns in total and emergent drug interactions were similar while the lower-order drug interactions were generally more additive than the other two interaction types (Figure S3C). The strong emergent interaction scores indicate that the synergies and antagonisms we observed in total drug interactions are not systematically due to lower-order effects. Together, these results suggest that drug interaction scores alone in the measured *in vitro* models were poor indicators of *in vivo* combination efficacy.

Synergistic drug combinations are not necessarily more effective than antagonistic combinations as the maximum effect of a combination can change independently of the drug interaction (Meyer et al., 2019) (see Box 1). A trade-off between synergy and efficacy appears to be important to consider when selecting effective drug combinations for treating other diseases (e.g., hepatitis C, HIV, and cancer), with maximum effect often being more important than synergy (Palmer et al., 2019; Sen et al., 2019). To determine if the maximum effect could be used to prioritize combinations from the DiaMOND compendium, we clustered the E_inf_ (a measure of maximum dose response effect, see Box 1) for all compendium drug combinations in all eight *in vitro* models at the terminal time point (Figure 2C). We observed a high maximum effect (E_inf_ > 0.9, Figure 2C) in most combinations, consistent with the drugs' known anti-Mtb effects. Dormancy and cholesterol-high models exhibited little variation in E_inf_, suggesting that neither condition had the dynamic range of maximum effect needed to discriminate among combinations or that all drug combinations are effective in these growth conditions for extended drug exposures. We compared E_inf_ profiles for the selected combinations we examined before, and we found that BPaL was more potent than HRZ or MRZ (Figure 2D), consistent with animal outcomes of these regimens (Tasneen et al., 2016; Xu et al., 2019; De Groote et al., 2011; 2012; Nuermberger et al., 2004a, 2004b, 2008; Rosenthal et al., 2007; Mourik et al., 2017). These examples suggest that maximum achievable effect *in vitro* may be a stronger predictor of outcomes in mouse models than *in vitro* synergy. As with E_inf_, we observed correct rank ordering in some *in vitro* models by other potency metrics (AUC_25_ and GR_inf_) (Figures S4A and S4B), though we identified no drug combinations in the DiaMOND compendium that were maximally potent across all eight models (Figures S4C and S4D). We also observed different relationships between *in vitro* models when either metrics or separate time points were investigated (Figures S4C and S4D), suggesting that metrics of potency across timescales provide non-redundant information about drug combinations. The correct ordering of selected drug combinations by mouse outcome suggests that the DiaMOND compendium contains useful information for identifying efficacious drug combinations.

### DiaMOND metric signatures are predictive of treatment outcomes in the relapsing mouse model

**Figure 3:**
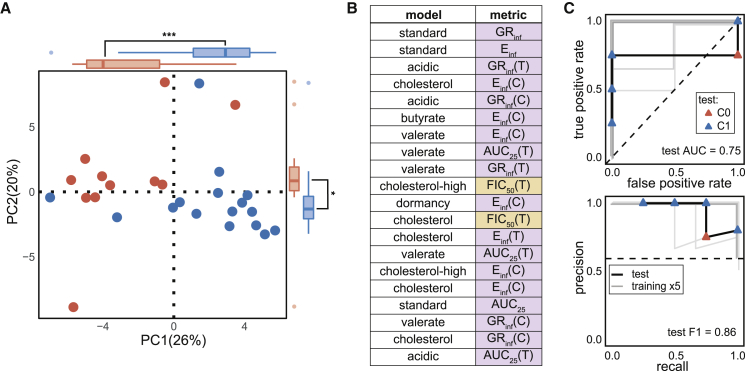
Prediction of combination treatment outcomes in the RMM with DiaMOND data (A) Projection of the DiaMOND compendium data from all *in vitro* models onto the first two PCs and a highlight of the percent variance explained by each PC. Outer box and whisker plots show the distributions of C1 and C0 combinations along PC1 and PC2 (Wilcoxon rank-sum test: ^∗∗∗^p < 0.005. ^∗∗^p < 0.01). Points are colored by outcome in the RMM (blue, C1, better than standard of care; red, C0, standard of care or worse). (B) Highest weighted features in PC1 with *in vitro* model (abbreviations in Figure 1A) and metric type indicated. Metrics are classified and shaded according to whether they are related to drug combination potency (purple: AUC_25_, E_inf_, and GR_inf_) or drug interaction (orange: FIC_50_ and FIC_90_). (C) ROC curves (top panel, Table 1) and PR curves (bottom panel, Table 1) of a RF-based classifier trained on all eight conditions in the DiaMOND compendium. The model is tested with high-order combinations (four- and five-drug combinations) that were excluded from training. Training (gray lines each show one of five cross validations; lines are slightly offset to aid visualization) and test (black) performances are shown with lines. Test combinations are colored by outcome class as in (A). Performance metrics are shown on plots for test data (area under the ROC curve [AUC] and F1, harmonic mean of precision and recall, Table 1). Dashed lines indicate theoretical “no-skill” model performance.

We hypothesized that combinations of *in vitro* measurements could be compiled to model the *in vivo* microenvironments experienced by Mtb during drug treatment. We asked whether signatures of DiaMOND compendium measurements could distinguish drug combinations that were better than the standard of care in animal studies, HRZE or HRZ (Table 1). We annotated 27 drug combinations that we measured in the compendium based on whether the treatment outcome in published RMM studies was better than the standard of care (C1) or not (C0) (Data S2). Most studies included a standard of care treatment making these annotations straightforward. For studies where no standard of care treatment was included, we inferred annotation by comparing with a study that shared at least one other drug combination treatment and that also included standard of care. This annotation limits the resolution of treatment improvement we can evaluate but does not necessitate normalization between studies (e.g., infection inoculum, drug treatment time, or Mtb strain) that might be needed for more quantitative assessment of treatment improvement. Principal component analysis (PCA) demonstrated that linear combinations of *in vitro* features could separate C0 and C1 drug combinations containing two and three drugs without using the *in vivo* class information (Figure 3A, Wilcoxon rank-sum, p < 0.005; Data S2). A separation was also apparent in principal component (PC) spaces of combinations with two, three, and four drugs (Figure S5A). Lastly, we also observed similar class separation when PCA was repeated using data from the constant and terminal time points separately (Figure S5B), indicating that similar conclusions would be drawn from any of the time point data. Because PCA does not use class labels in its computation, the observed separation of *in vivo* classes suggests that *in vitro* measurements from the DiaMOND compendium contain strong signal that characterizes the performance of drug combinations *in vivo*. Inspection of feature contributions to PC1, which best separates C0 and C1 drug combinations (Figure 3A, Wilcoxon rank-sum, p < 0.005; Data S3), revealed many features from the cholesterol, standard, and valerate growth models (Figure 3B; Data S3). We also observed that potency metrics (AUC_25_, E_inf_, and GR_inf_) are almost exclusively represented in the top-20 contributing features (Figure 3B). Together these results suggest that effective separation of C1 and C0 drug combinations requires measurement of drug combination potency in multiple growth environments.

To better separate C0 and C1 combinations based on the signals observed in PCA and make predictions for new combinations, we trained binary classifiers with eight different machine-learning (ML) methods to distinguish C0 and C1 drug combinations and compared their performance in 5-fold cross validation. We observed that nonlinear ensemble methods (Bayesian additive regression trees, random forest (RF), and gradient boosted trees) outperformed other ML algorithms, as measured by the area under the receiver operator characteristic (ROC) curve (AUC) and the F1 statistic, which is the harmonic mean of precision and recall (Table S2). We performed additional validation of the RF model by applying it to higher-order (four- and five-way) drug combinations commonly used in preclinical and clinical tests that were not considered during model training (Data S4). The RF model accurately predicted outcomes (Figure 3C, AUC = 1, F1 = 0.86) and exhibited performance similar to what was estimated in cross validation. We noted that for validating the RMM classifier, there were five higher-order combinations for which we had DiaMOND data in all eight conditions (Figure 3C). To determine whether the five test combinations of the eight-condition model represented a generalized measure of performance, we retrained a classifier using seven conditions (all but the intracellular model) from which we had more test data available (14 combinations). We observed similarly high validation performance in the seven-condition model (Figure S5C, AUC = 0.79, F1 = 0.87), suggesting that the performance of the eight-condition model is not simply an artifact of the small test set.

Because drug combination design and testing in animal models is iterative (Figure 1A), we encounter an unavoidable overlap of drugs between many of the combinations used for model training (low-order) and validation (high-order, Data S2). We asked how much this overlap contributes to the observed classifier performance by systematically considering training/test splits with one-, two-, and three-drug overlaps (see STAR Methods). As expected, we found that validation performance increased with higher drug overlap between the model training and validation combinations (Figure S5D). A major goal of drug regimen design is to understand how to best use new drugs, rather than avoid overlap with previously tested combinations; for example, by substituting a new drug for an existing drug in a well-tested combination based on combination efficacies in animal models (Ginsberg and Spigelman, 2007; Kerantzas and Jacobs, 2017; Tiberi et al., 2018a, 2018b; Wallis et al., 2016; Lienhardt et al., 2019). We therefore sought to evaluate model performance on combinations containing a drug that had not been encountered by the model during training (“leave-one-drug-out”). We observed that for most (seven) drugs, the model was able to accurately predict whether the addition of that drug to a lower-order combination would result in improvement over the standard of care (Figure S5E; AUC > 0.7 and F1 > 0.66). Taken together, these results indicate that our modeling structure is well matched to the data generated during TB drug regimen design, where combinations are constructed iteratively so that a few combinations with new antibiotics of interest are tested early for efficacies in animal models.

**Figure 4:**
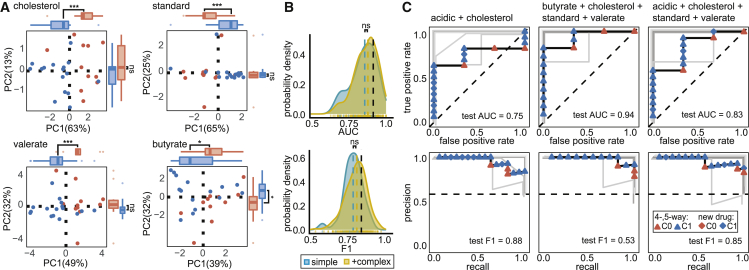
Prediction of combination therapy outcomes in the RMM using fewer *in vitro* models (A) Projection of the DiaMOND data from single *in vitro* conditions (subplots) onto the first two PCs and the percent variance explained by each PC (plots are labeled as in Figure 3A). Outer box and whisker plots show the distributions of C1 and C0 combinations along PC1 and PC2 (Wilcoxon rank test: ^∗∗∗^p < 0.005, ^∗∗^p < 0.01, ^∗^p < 0.05; ns, p > 0.05). (B) Density distribution plots of estimated classifier performances from systematic survey of all possible *in vitro* model subsets. Distributions of ROC AUC (top) and F1 (bottom) are separated based on whether technically complex models (intracellular, cholesterol-high, dormancy) are included (yellow) or whether only simple conditions (acidic, butyrate, cholesterol, standard, and valerate) are considered. Colored dashed lines indicate mean value for distribution. The estimated performances when using all *in vitro* models (as in Figure 3) is shown with black dashed lines. Distributions are compared with a Wilcoxon rank-sum test (ns, not significant). (C) Comparison of classification performances of three high-performance random forest classifiers using subsets of simple *in vitro* models. Training (gray lines each show one of five cross validations; lines are slightly offset when they are on top of each other) and test (black) performance is demonstrated with ROC (top) and PR (bottom) curves. Test combinations are colored by outcome class as in (A). Plot shapes indicate whether a test combination contained higher-order four- and five-drug combinations (triangle) or a combination containing a new drug (diamond-shape) not included in the compendium described in Figure 1. Dashed lines indicate theoretical “no-skill” model performance.

We observed that some of the *in vitro* models in the DiaMOND compendium are well represented among the top-ranked features in the classifying PCs (Figure 3B). In contrast, other *in vitro* models are not present, suggesting that a subset of *in vitro* models may be sufficient to predict treatment outcome in the RMM. We asked whether classifiers using the DiaMOND compendium data from one *in vitro* model at a time were predictive of RMM outcome class. A Horn’s parallel analysis (Figure S5F) identified the first four PCs as explaining more variance than expected by chance. We then observed that the data signal separating C0 and C1 drug combinations appeared in at least one of the first four PCs for all eight *in vitro* models (Figure 4A, Wilcoxon rank-sum test, p < 0.05; Figure S5G). Furthermore, the five technically simpler models to work with exhibited clear C0 and C1 separation (Figure 4A, *in vitro* models cholesterol, butyrate, standard, valerate; Figure S5G *in vitro* model acidic).

Though the single *in vitro* model classifiers were moderately predictive, they did not perform as well as the classifier trained using data from all eight *in vitro* models (Data S3). We asked whether another high-performing classifier could be derived using a subset of *in vitro* models. We systematically trained RF classifiers by considering all possible model combinations and observed that among the 255 possible combinations of *in vitro* models, 67 (26.3%) performed better than the classifier trained on all eight models (Data S4). Furthermore, predictors including only the simpler *in vitro* models performed as well or better than those including the “complex” (intracellular, dormancy, and cholesterol-high) models (Figure 4B, Student’s t test, p > 0.05). We further validated the highest performing classifiers trained on the simple *in vitro* models by applying them to the higher-order (four- and five-way) drug combinations as well as drug combinations involving antibiotics (delamanid, sutezolid, and SQ109, Table S1) that were not included in the compendium’s ten-drug set (Data S2). SQ109 represents a drug in a new antibiotic class, while delamanid and sutezolid are in the same antibiotic classes as drugs in the 10-drug set (pretomanid and linezolid, respectively). The high performance of classifiers on this validation set suggests that computationally combining simple *in vitro* models can produce classifiers that inform possible RMM outcomes (Figure 4C) and that there may be multiple combinations of *in vitro* models that are predictive of outcomes in the RMM.

With many high-performing RMM classifiers trained using subsets of the five simple *in vitro* models (Data S4; Figure 4C), we assessed whether the predicted RMM outcome for specific drug combinations would be consistent between these classifiers. The classifiers produce a probability that a drug combination belongs to each class (e.g., drug combination X belongs to C1 with 60% probability and C0 with 40% probability). The threshold probability is usually at 50% to assign the classification, but the probability can also rank combination classification likelihood. We tabulated the predicted probabilities of outcome for all combinations in the compendium, as well as the higher-order and new drug combination validation set, using the top-performing simple *in vitro* model classifiers shown in Figure 4C. As we had previously observed, rank ordering the percent probabilities within each classifier shows high predictive performance when evaluating the validation set (Data S4). Among all predictions made for the compendium and validation combinations, we noted that 36% of drug combinations had discordant predictions among the three classifiers. We did not observe a consistent pattern in which a classifier was discordant. We next tested whether a consensus prediction could be generated by simply averaging the probabilities of the top three classifiers. We observed that the discordant combinations were clustered in the second quartile (probability of C1 around 25%–50%), suggesting that classifiers are most prone to error for combinations that are C0. This may be due to the mild class imbalance in the training set (11 C0 and 16 C1 combinations). The consensus prediction was highly accurate (84% on validation set and 93% overall). Incorrect consensus predictions were at the border between C0 and C1 at 42%–47% C1, indicating that the misclassification was due to ambiguity near the 50% decision boundary instead of strong classifier discordance. We conclude that a simple averaging of the probabilities generated by top classifiers is a practical means to construct an accurate consensus rank ordering for predicting drug combination response outcomes for RMM.

### DiaMOND metrics describe the efficacy of drug combination treatments in the C3HeB/FeJ mouse model

We next asked whether the DiaMOND compendium contains information useful for distinguishing outcomes in other mouse models. Bactericidal activity in the most commonly used mouse strains (e.g., BALB/c, C56BL/6, and Swiss) has been used extensively to evaluate drug combination effectiveness. Bactericidal activity in these models (bactericidal mouse model, BMM, Table 1) measures the reduction in bacterial burden during and immediately following drug treatment and can be assessed more quickly than relapse. Using the same analysis process, we investigated whether drug combinations with different BMM outcome classes (Data S2) were separable in the top PCs. In contrast to RMM classes, we observed no separation of BMM C0 and C1 in the top two PCs (Wilcoxon rank-sum test, p > 0.05, Figure S6A). Consistent with the lack of separation in the PC space, we found that ML classifiers could not predict C0 or C1 drug combinations for the BMM outcome (Data S3; AUC = 0.67, F1 = 0.40; Figure S6B). Additional analysis of *in vitro* model subsets identified many predictors with improved performance, but this improvement did not generalize to test data (Data S4). Moderate model training performance and poor generalizability to new data suggest that the drug combination information needed for BMM outcome predictions may be difficult to capture with the *in vitro* models developed and used in this study.

The C3HeB/FeJ (HeB) mouse strain has become important for TB regimen development because the disease pathology is more similar to humans than other mouse strains (Driver et al., 2012; Harper et al., 2012; Kramnik and Beamer, 2016). This includes the formation of caseous, necrotic granulomas that are characterized by low oxygen content (hypoxia) (Driver et al., 2012; Harper et al., 2012; Irwin et al., 2015) and differential drug penetrance (Irwin et al., 2016; Dartois, 2014). These lesions also contain large numbers of extracellular, non-replicating bacteria (Nuermberger, 2017; Irwin et al., 2015). HeB mice are used to measure both use bactericidal (BHeB) and relapse outcomes to determine treatment efficacy. Fewer drug combinations have been tested and published using HeB mice than other mouse strains. The DiaMOND compendium contained too few measured combinations to train ML classifiers. When we integrated the compendium combinations with higher-order drug combinations, we obtained a total of 16 combinations (Data S2) for the BHeB outcome, which was sufficient to train ML classifiers. However, we were not able to do the same for the relapse outcome, where we had four total combinations, even after augmenting with higher-order information.

**Figure 5:**
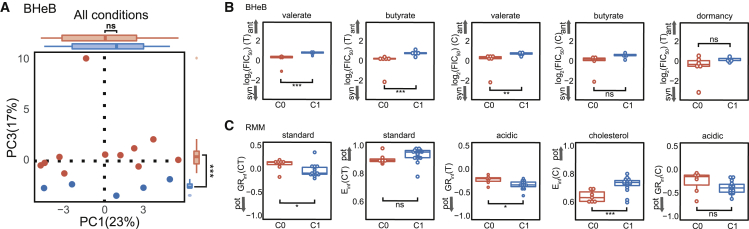
Signatures of DiaMOND data to describe outcomes in multiple mouse models (A) Projections of the DiaMOND data onto PC1 and PC3 and the percent variance explained by each PC Points are colored by outcome in the BHeB (blue: C1, better than standard of care; red: C0, standard of care or worse). (B and C) Values of the four highest weighted features in the most discriminatory PC are compared for C1 and C0 combinations in the BHeB (B) and RMM models (C) using dot and box plots. The top features in BHeB are drug interaction metrics whereas the top features are potency metrics in RMM. High (pot) versus low potency and synergy (syn) versus antagonism (ant) is indicated with arrows on each subplot. (Wilcoxon rank test: ^∗∗∗^p < 0.005, ^∗∗^p < 0.01, ^∗^p < 0.05; ns, p > 0.05).

To understand if DiaMOND metrics distinguish C0 and C1 BHeB combinations using the training dataset including lower- and higher-order combinations, we evaluated class separation with PCA. A Horn’s parallel analysis identified the first four PCs as explaining more variance than expected by chance (Figure S7A). We observed significant separation of BHeB outcome classes along the third PC (PC3) (Figure 5A, p < 0.005; Data S3; Figure S7B). We then examined the top ten features in PC3 by contribution (Data S3) and found that the *in vitro* models and metrics were distinct from those we observed in the RMM analysis (Figure 3B). Notably, the metrics for the BHeB were entirely drug interactions, and the presence of the dormancy model in the top ten features was of particular interest because we expected hypoxia-induced dormancy to be a microenvironment specific to the C3HeB/FeJ mice (Irwin et al., 2015; Harper et al., 2012; Driver et al., 2012). Using the same approach described for RMM, we developed accurate RF models to classify BHeB C1 and C0 combinations (all *in vitro* models, AUC = 0.9, F1 = 0.80, Data S4). Systematic evaluation of RF classifiers using all possible combinations of *in vitro* model subsets revealed that complex models did not improve performance (Figure S7C). Specifically, we found that models without dormancy perform as well as those with it (Figure S7D). As with the RMM classifiers, we identified *in vitro* model subsets that performed better than all models together trained for the BHeB outcome (37 [12.9%], Data S4). Lipid and acidic *in vitro* models featured prominently among the most accurate classifiers (Data S4). Together, these analyses demonstrate that the DiaMOND compendium data predict outcomes in two pathologically distinct mouse models, suggesting that enough key information can be captured by simple *in vitro* models to prioritize combination therapies for animal model tests.

### Potency and antagonism are correlated with improved outcomes in mouse models

The signatures of DiaMOND data describing outcomes in RMM and BHeB highlighted that potency metrics were key predictors for RMM, while drug interactions were key for BHeB outcome classification. To understand whether C0 and C1 drug combinations showed significant differences in these metrics, we examined the top four features from the most discriminatory PCs for both mouse models. Univariate analysis revealed significant differences between C1 and C0 combinations for three of the top four features describing either the BHeB, or RMM outcomes (Figures 5B and 5C, Wilcoxon rank-sum, p < 0.05). That *in vitro* antagonistic drug combinations may be more favorable is consistent with the results of our comparison of BPaL to the standard of care (HRZ, Figure 2B). For BHeB outcomes, drug interactions were more antagonistic for C1 than for C0 combinations (Figure 5B). C1 combinations in the RMM outcome were more potent than C0 combinations (Figure 5C), which is consistent with expectations of increased potency for the most effective drug combinations. We found that different metric types (potency or interactions) may provide information that maps to different outcome types (bactericidal or relapse) in animal studies. Furthermore, our analysis suggests that high potency and antagonism in *in vitro* assays may be characteristics of favorable drug combinations.

## Discussion

Our goal in this study was to develop a workflow to efficiently prioritize drug combinations early in the TB regimen design process. Most *in vitro* drug efficacy studies utilize single growth conditions, which have not been clearly mapped to *in vivo* outcomes (Nuermberger, 2017; Parish, 2020). Furthermore, conflicting results from multiple *in vitro* models have not been readily resolvable. We hypothesized that treatment efficacy *in vivo* could be modeled as a “sum of parts” of the complex microenvironment. Therefore, we generated a dataset that profiles drug combination effects against Mtb in eight different *in vitro* growth environments. With this comprehensive drug combination data compendium, we identified signatures of potencies and drug interactions in specific *in vitro* models that distinguish whether drug combinations are better than the standard of care in two important preclinical mouse models. PCA on the compendium data alone was able to separate drug combinations that show treatment improvement in these mouse models. We found that ML classifiers were accurate predictors of mouse disease relapse using data from only a few simple *in vitro* models. These classifiers were validated with higher-order (4 and 5 drugs) combinations and had predictive power for combinations with drugs not included in the model training. Our classifiers perform best when a drug of interest is included in some combinations in the training set. Nonetheless, we expect that our buildable approach to use systematic *in vitro* data to prioritize drug combinations for *in vivo* study will have immediate benefit. As more antibiotics with more diverse mechanisms of action are tested in animals and added to DiaMOND datasets, we anticipate that we will be able to make accurate predictions of untested combinations containing these new antibiotics. We can therefore leverage these data with more expansive DiaMOND measurement to prioritize combinations for further testing *in vivo*. Together, our study establishes a practical approach to prioritize combination therapies using economical, scalable, and expandable *in vitro* measurements while maximizing the use of *in vivo* efficacy data that are generated early in TB drug development.

Synergy is often assumed to be a property of optimized combination therapies because synergistic drugs are more effective together than expected based on single-drug efficacies alone. Our mapping of the DiaMOND compendium onto outcomes in two different mouse models challenges this notion. In the relapsing mouse model, drug interactions were not key features for classification; instead, the potency measures from the drug-dose response curves were the most important predictors of outcome (Figure 3B). Our findings are consistent with reports of treatment in hepatitis C, cancer, and HIV (Palmer et al., 2019; Palmer and Sorger, 2017; Sen et al., 2019) that show a trade-off between maximizing synergy and potency of a drug combination. Maximizing potency was often more important than synergy in treating these diseases with multidrug therapies (Palmer et al., 2019; Palmer and Sorger, 2017; Sen et al., 2019). Antagonism was prevalent in our compendium (Figure 2A), and we found that antagonism was characteristic of more efficacious drug combinations for the C3HeB/FeJ bactericidal model (Figure 5B, C1 more antagonistic than C0). Partnering the most potent drugs together during regimen design may be generating highly potent combinations but biasing these combinations toward antagonistic *in vitro* drug interactions. Bedaquiline, pretomanid, and linezolid were recently found to be more potent in treating mice infected with the Mtb HN878 strain than the H37Rv strain (Bigelow et al., 2020). When combined, the drugs antagonized each other for treating Mtb strain HN878-infected mice. Despite this antagonism, the BPaL combination was highly effective at curing mice infected with either Mtb strain. These *in vivo* results are consistent with our findings that BPaL is a highly potent but antagonistic drug combination for *in vitro* treatment of Mtb Erdman. One view of how drugs in combination exert their effect on cell populations is that each drug targets a different subpopulation rather than multiple drugs targeting the same cells (Palmer et al., 2019; Palmer and Sorger, 2017). Drug interactions would then explain how well a drug acts on the cellular population that was not susceptible to the other drugs in the multidrug treatment. This leads to the hypothesis that very potent drugs that alone can kill most of the cells in a population would achieve high maximum effect when combined but may tend toward antagonism rather than synergy. Study of the multidrug anti-cancer therapy R-CHOP (Table 1) supports this hypothesis (Palmer et al., 2019) and an expanded study using more antibiotics could be used to test this hypothesis in tuberculosis. Our study suggests that for TB, potent drug combinations should be prioritized for further study and should not necessarily be deprioritized if they are antagonistic in *in vitro* assays.

Our approach enabled us to determine the relative importance of specific *in vitro* models to distinguish combinations with treatment improvement in mice, thereby serving to validate which growth conditions map to *in vivo* responses. The cholesterol *in vitro* model was the top-performing single *in vitro* model classifier for the RMM outcome and performed almost as well as the classifier with all *in vitro* models (Table S3; Data S3). This is consistent with the importance of cholesterol metabolism for Mtb survival and infectivity (Wilburn et al., 2018). We also observed that other lipid-rich environments induced distinct drug response patterns and that the best classifiers for both RMM and BHeB outcomes utilized metrics from multiple lipid-rich growth conditions. These findings suggest that measuring drug combination responses with a suite of simple growth environments may be sufficient to model the complex lipid environment encountered in TB lesions.

Mtb in the RMM mouse strains are thought to be primarily intracellular (Nuermberger, 2017), and intracellular Mtb are exposed to the acidification of the phagolysosome (Baker et al., 2019; Vandal et al., 2009). Therefore, we expected the acidic growth environment to be important for determining treatment improvement for the RMM. We found that measurements from the acidic growth environment alone were not strongly predictive of outcomes in the RMM but that these metrics were prominent in the best mixed-condition classifiers. Furthermore, other single growth environment models separated classes better than the acidic model (Table S3). These results indicate that response to acidic stress is important for Mtb intracellular survival to drug treatment in the RMM, but adaptation to other environmental factors (such as lipid carbon sources) are important drivers of treatment response. We also observed that the acidic model was prominent among the best classifiers for the bactericidal outcome in the C3HeB/FeJ mouse strain (BHeB outcome, Data S4). The C3HeB/FeJ mice are noted for the formation of the caseous necrotic granulomas (type-I lesions; Irwin et al., 2015) that have been shown to have a neutral pH (pH > 7) (Sarathy and Dartois, 2020; Lanoix et al., 2016b), high-lipid content (Driver et al., 2012; Kim et al., 2010; Guerrini et al., 2018), and with primarily extracellular Mtb (Irwin et al., 2015; Lenaerts et al., 2015). However, like the BALB/c mice which only form lesions with intracellular Mtb, these animals have abundant intracellular bacteria in other lesion types and within macrophages that acidify the intracellular Mtb compartments (Irwin et al., 2016), which may explain why acidic growth environments are important predictors of drug response in this mouse model. Furthermore, Mtb residency in lipid-laden, foamy macrophages is important in both the BALB/c (and similar mouse strains; Shim et al., 2020; Zhang et al., 2011; Rosenthal et al., 2012; Driver et al., 2012) as well as C3HeB/FeJ mice (Driver et al., 2012; Rosenthal et al., 2012), supporting the idea that *in vitro* lipid growth conditions provide important information about *in vivo* drug response for both the RMM and BHeB model.

Use of both acidic and lipid-rich *in vitro* models in developing classifiers is an example of how we were able to combine measurement in a “sum of parts” approach from relatively simple growth environments to model treatment outcomes despite the complexity of the microenvironments in TB lesions (Figures 4 and S5G). These results suggest that there is predictive drug combination response information obtained from simple *in vitro* models that only needs to be combined correctly to predict drug treatment outcomes in mice. The practical implication is that researchers can choose a subset of the most amenable *in vitro* models for performing drug combination experiments and still retain predictive capacity. Despite the success of summing together simple “parts” in predicting coarse outcome classifications, the importance of combination potency and variation of potency among growth conditions raises the question of whether measurement in more complex growth environments (e.g., reflecting multiple aspects of the host microenvironment) would further refine the classifications and improve prediction accuracy. The richness of this DiaMOND dataset will also enable future studies to understand whether *in vivo* combination outcomes can be predicted using few *in vitro* data, for example by using lower-order combination data alone.

Several changes to the experimental design may improve the experimental and computational workflow developed in the current study. The importance of potency metrics in signatures of combination efficacy is perhaps surprising given that we design combination dose responses to have equipotent combinations of each drug. There is growing evidence that there is differential drug penetration into the lesions where Mtb is found (Dartois, 2014), which would lead to non-equipotent levels of drug reaching Mtb cells. Utilizing pharmacokinetic data to design drug combinations may increase this approach's utility and power and lead to a more predictive DiaMOND compendium dataset. The current standard of care and other new regimens (e.g., “Study 31” and SimpliciTB) involve intensive and continuation phases of treatment. Including sequential treatments in an experimental approach could help understand how prior treatment sensitizes the bacterial population to future treatment regimens. One reason to use combination therapy for TB is to slow the acquisition of drug resistance. A systematic study of the drug combination space in different growth environments can also be used to investigate the evolution of drug resistance. For example, antagonistic drug interactions have been shown to suppress the evolution of drug resistance (Palmer et al., 2019; Yeh et al., 2006; Michel et al., 2008; Coates et al., 2020) and the evolution of drug resistance can be tied to the growth rate and duration of drug exposure (Bigelow et al., 2020; Yeh et al., 2006; Liu et al., 2020; Greenfield et al., 2018). Though this study was not designed to test the impact of combination therapy on development of drug resistance, future studies could adapt the DiaMOND experiments to assess drug resistance using luminescence reporters (Zhang et al., 2012a). We did not directly evaluate drug resistance in our assays, but we did not observe outgrowth over time at high drug concentrations in the longer drug treatments, suggesting that any resistance development that occurred did not overtake the populations and influence dose response measurements. The depth of the DiaMOND compendium may also be well complemented with transcriptomic data of drug response to prioritize drug combinations based on predicted mechanisms of drug interaction (Ma et al., 2019). We expect this pipeline to improve as the component drug set is expanded and diversified via new drug discovery methods (Hie et al., 2020; Johnson et al., 2019; Rock, 2019; Rock et al., 2017; VanderVen et al., 2015; Bryk et al., 2008; Gold and Nathan, 2017; Gold et al., 2015) and as more animal studies provide outcome data for drug combinations with new combinations and drugs. We also note that the size of drug combination classification sets used in this study was achieved by compiling and giving equal weight to diverse animal studies from multiple investigators with differences in study designs (e.g., infection protocols, drug treatment times, Mtb strains, etc.). Developing and validating across-study standardizations may improve future analysis, modeling, and predictions. Mtb drug response and virulence can be strain dependent (De Groote et al., 2012; Bigelow et al., 2020), and the strain of Mtb used in this study (Erdman) differed from those used in some of the animal studies (e.g., H37Rv). In future work, including multiple Mtb strains (including clinical isolates) may improve combination classification separation and model predictions.

## STAR★Methods

### Key resources table

**Table:** 

REAGENT or RESOURCE	SOURCE	IDENTIFIER
**Bacterial and virus strains**		

*Mycobacterium tuberculosis*: Strain Erdman	ATCC	ATCC 35801

**Chemicals, peptides, and recombinant proteins**		

bedaquiline	NIH AIDS Reagent Program	N/A
clofazimine	Sigma	C8895
ethambutol	Sigma	E4630
Isoniazid	Sigma	I3377
linezolid	Sigma	PZ0014
moxifloxacin	Sigma	SML1581
pretomanid	TB Alliance	N/A
pyrazinamide	Sigma	PHR1576
rifampicin	Sigma	R3501
rifapentine	Sigma	R0533
delamanid	VWR	10189
sutezolid	Sigma	PZ0035
SQ109	Fisher	50-186-7024
gatifloxacin	Sigma	32345
d-cycloserine	Sigma	C6880

**Deposited data**		

Data cube and IC_90_ table	This study	https://doi.org/10.17632/m2y7jpz4wz.1

**Experimental models: Cell lines**		

Mouse: J774A.1	ATCC	ATCC TIB-67

**Recombinant DNA**		

pMV306hsp+LuxG13	Andreu et al., 2010	http://n2t.net/addgene:26161; RRID:Addgene_26161

**Software and algorithms**		

Code for modeling, and figure generation	This study	supplemental information
MATLAB	N/A	https://www.mathworks.com/products/matlab.html
R	N/A	https://www.R-project.org/
tidyverse(R package)	Wickham et al., 2019	https://CRAN.R-project.org/package=tidyverse
ggplot2(R package)	Wickham, 2016	https://CRAN.R-project.org/package=ggplot2
ggpubr(R package)	N/A	https://CRAN.R-project.org/package=ggpubr
openxlsx(R package)	N/A	https://CRAN.R-project.org/package=openxlsx
readxls(R package)	N/A	https://CRAN.R-project.org/package=readxl
stats(R package)	N/A	https://www.R-project.org/
paran(R package)	N/A	https://CRAN.R-project.org/package=paran
mlr(R package)	Bischl et al., 2016	https://CRAN.R-project.org/package=mlr
bartMachine(R package)	Kapelner and Bleich, 2016	https://CRAN.R-project.org/package=bartMachine
randomForestSRC(R package)	Ishwaran et al., 2008	https://CRAN.R-project.org/package=randomForestSRC
xgboost(R package)	Chen and Guestrin, 2016	https://CRAN.R-project.org/package=xgboost
e1071(R package)	N/A	https://CRAN.R-project.org/package=e1071
kknn(R package)	N/A	https://CRAN.R-project.org/package=kknn
rstatix (R package)	N/A	https://CRAN.R-project.org/package=rstatix
wPerm (R package)	N/A	https://CRAN.R-project.org/package=wPerm

### Resource availability

#### Lead contact

Further information and requests for resources and reagents should be directed to and will be fulfilled by the lead contact, Bree Aldridge (bree.aldridge@tufts.edu).

#### Materials availability

Autoluminescent *M. tuberculosis* strain generated in this study is available upon request.

### Experimental model and subject details

#### Bacterial cell lines and culture

*M. tuberculosis* Erdman strain was transformed with pMV306hsp+LuxG13 to generate an autoluminescent strain that was used for all experiments in this study (Addgene plasmid # 26161; http://n2t.net/addgene:26161; RRID:Addgene_26161) (Andreu et al., 2010)). Standard 7H9 Middlebrook medium supplemented with 0.2% glycerol, 10% OADC (0.5g/L oleic acid, 50g/L albumin, 20g/L dextrose and 0.04g/L catalase) and 0.05% Tween-80 with 25 μg/mL kanamycin was used for Mtb strain maintenance. Growth and culturing were performed at 37°C with aeration unless noted. Cells were passaged before reaching OD_600_ = 0.8. Standard 7H10 Middlebrook agar plates supplemented with 0.5% glycerol, 10% OADC, 0.05% Tween-80 and 25 μg/mL kanamycin were used for enumerating colonies.

#### Mammalian cell lines and cell culture

We used the mouse cell line, J774, as a model of intracellular residency because J774 cells have been used as a macrophage-like cell line to study early infection processes and Mtb drug response to complex host-like intracellular environment (Pires et al., 2015; Stanley et al., 2012). J774 cells were cultured in high glucose DMEM supplemented with 2mM L-glutamine, 1mM sodium pyruvate, and 10% heat-inactivated fetal bovine serum (FBS) at 37°C in 5% CO_2_ as previously described (Stanley et al., 2012). Media was changed every one-three days and cells passaged at ∼80% confluence.

### Method details

#### Generation of autoluminescent Mtb strain

Autoluminescent *M. tuberculosis* strain was generated by transforming the Erdman parent strain with pMV306hsp+LuxG13, resulting in a single copy chromosomal integration of the bacterial luciferase operon. The pMV306hsp+LuxG13 plasmid contains a reorganized and codon-optimized bacterial luciferase operon for maximum mycobacterial light production (Andreu et al., 2010). Briefly, the construct was electroporated into Mtb, and kanamycin resistant colonies were isolated and tested for auto-luminescence. These positive strains were expanded in standard 7H9 supplemented media and frozen down. The frozen stocks were used as the starting strains for *in vitro* model acclimation and drug combination experiments.

#### Mtb *in vitro* model acclimation

All *in vitro* model media were buffered with 100 mM 3-(N-morpholino)propanesulfonic acid (MOPS, pH 7), unless noted, and filter-sterilized prior to use. The acidic model was based on the standard 7H9 Middlebrook media above and buffered with 100 mM 2-(N-morpholino)ethanesulfonic acid (MES) and adjusted to pH 5.7. For acclimation to lipid carbon sources, a base medium consisting of 7H9 powder (4.7g/L), fatty acid-free BSA (0.5g/L), NaCl (100mM) and tyloxapol (0.05%) with 25 μg/mL kanamycin was used and the lipids sodium butyrate (5mM, final concentration), valeric acid (0.1% final concentration) or cholesterol (0.05mM or 0.2mM final concentration) were added to the base medium. For the cholesterol media, a cholesterol stock solution (100mM) was first prepared by dissolving cholesterol in a 1:1 (v/v) mixture of ethanol and tyloxapol and heated to 80°C for 30 minutes and added to pre-warmed (37°C) base medium (Lee et al., 2013). The dormancy medium was based on the butyrate medium with the addition of sodium nitrate (5mM) as a terminal electron acceptor (Cunningham-Bussel et al., 2013a,2013b; Gold and Nathan, 2017; Sohaskey, 2008).

Mtb were inoculated into standard 7H9 Middlebrook medium, grown to mid-log phase (optical density, OD_600_ ∼0.5-0.7) and were subcultured for less than two weeks prior to acclimation to assay medium. For acclimation to standard and acidic media, Mtb cells were diluted into assay media at a starting density of OD_600_ = 0.05, acclimated for 3-5 doubling times or until they reached mid-log phase (OD_600_ ∼0.5-0.7), diluted to OD_600_ = 0.05 and grown back to mid-log phase before use in DiaMOND assays.

Similar to standard, and acidic conditions, Mtb were acclimated to butyrate, and valerate media and acclimated cells were frozen for use in assays. Frozen acclimated Mtb in butyrate and valerate media were inoculated into assay media, grown to mid-log phase (OD_600_ ∼0.5-0.7), diluted into fresh lipid media at a starting concentration of OD_600_ = 0.05 and grown back to mid-log phase (OD_600_ ∼0.5-0.7) and used for DiaMOND assays. The dormancy model used Mtb acclimated to butyrate medium grown to mid-log phase (OD_600_ ∼0.5-0.7) and then diluted to a starting OD_600_ 0.05 in dormancy media. For the dormancy model (d), cells were incubated at 37°C without aeration for 28 days, which reduced autoluminescence close to media-only background levels, which we interpret as being dormant with very low metabolic activity.

Mtb growth on cholesterol media slowed without the exchange of fresh medium. Cholesterol and cholesterol-high acclimation were similar to standard and acidic conditions with fresh media exchanges every seven days to ensure continued growth. Mtb acclimated between 14 and 28 days were used for assays. Mtb growth rate on cholesterol-high was faster (four day doubling time) than cholesterol (seven day doubling time).

For the intracellular model, J774 cells were plated at 375,000 cells/mL in 384-well plates and cultured overnight, expecting ∼one doubling prior to infection. Mtb grown to mid-log phase in standard media was syringe-passed 8 times with a 25-gauge needle to reach a single-cell suspension, and J774s were infected with Mtb at MOI 2 for 24 hours followed by drug treatment for 5 days.

#### Drugs, dose responses, and dispensing

The drugs used in this study are listed in Table 1. All drugs were reconstituted and diluted in DMSO except for pyrazinamide for the intracellular model; to avoid exceeding the DMSO limit (0.5%) in the intracellular condition, pyrazinamide was diluted in 1x phosphate-buffered saline with 0.01% Triton-X. Drugs were dispensed with an HP D300e digital dispenser, and locations were randomized to reduce plate effects. For each *in vitro* model, the concentration to achieve 90% inhibition (IC_90_) was determined. IC_90_ were used to design combination dose responses with equipotent mixtures of drugs (Cokol et al., 2017). A ten-dose resolution with 1.5- or 2-fold dose spacing was used for all experiments.

#### Benchmarking luminescence measurements

Decreases in autoluminescent Mtb have been shown to correspond to decreases in optical density and colony forming units (Andreu et al., 2010; Zhang et al., 2012a), specifically in response to drug treatment (Zhang et al., 2012a; Sharma et al., 2014). Luminescence must be used for the intracellular model because optical density measures both the mammalian and Mtb cells. We chose to use luminescence measurements for the dormancy model because the optical density measurements were highly variable. To more directly compare luminescence dose responsiveness from the intracellular and dormancy models to the optical density dose responsiveness of the other *in vitro* models, we sought to benchmark luminescence to growth inhibition. In the intracellular model, drug treatment was performed as described above. Luminescence was measured six days after infection (five days after addition of drugs, Constant/Terminal time point). Mtb were then lysed from macrophages with 0.01% sodium dodecyl sulfate (SDS) in distilled water for 15 minutes at 37°C, 10-fold serially diluted with standard 7H9 media and plated on 7H10 Middlebrook agar for colony forming unit (CFU) enumeration. Mtb dormancy was established as described above and treated with drugs. At the appropriate constant and terminal time point, luminescence was measured. Mtb were then 10-fold serially diluted with standard 7H9 media and plated on 7H10 Middlebrook agar for CFU enumeration. Normalized luminescence inhibition was calculated as described below and correlation assessed using “polyfit” in MATLAB.

#### Dose centering

For every *in vitro* model, each single drug was tested to identify the IC_90_ (concentration to inhibit 90% growth). Each dose response was ten units and the IC_90_ for single drugs was designed to be between dose 6 and dose 9. Drug combinations were designed for dosing to be equipotent around the IC_90,_ and doses were spaced 1.5x or 2x apart to capture the drug's full range of response.

#### Treatment and DiaMOND assays

Mtb were acclimated to *in vitro* model media prior to drug treatment as described above. For acidic, butyrate, cholesterol, cholesterol-high, standard, and valerate models: 50μL of acclimated Mtb at the indicated density was added to each well in 384-well plates containing freshly dispensed drugs and incubated at 37°C in humidified bags to prevent evaporation. Edge wells contained media but were not used for assays. For the dormancy model: Mtb were acclimated as described above, gently resuspended, and 20μL of dormant Mtb culture was transferred to each well on the assay plates. Plates were sealed with PCR seals to reduce oxygen exposure during drug treatment and incubated for seven days. We measured regrowth after drug treatment as a readout of drug effect during dormancy. Therefore, after drug treatment, plate seals were removed, 80μL of standard media was added to each well, and plates were incubated at 37°C in humidified bags to prevent evaporation. For the intracellular model: drugs were printed into media-only plates and transferred onto infected J774 cells 24 hours after Mtb infection. To accommodate quality control assessment, we included multiple untreated and positive drug treatment controls in each plate as well as uninfected J774 cells for the intracellular model.

#### Plate measurements

Luminescence and OD_600_ measurements were made at three-five time points per sample on a Synergy Neo2 Hybrid Multi-Mode Reader. Time points were based on the approximate doubling time of each model. To simplify the analysis, we generally compare time points at either a relatively similar time point (constant) or time ∼4-5x doubling times after drug exposure (terminal time point). Constant and terminal time points correspond to the same set of measurements for the standard and intracellular *in vitro* models (constant/terminal, CT). For the dormancy model, plate readings were made during recovery in standard media, and time points were selected based on doubling time in standard media. For the dormancy and intracellular models, OD_600_ measurements could not reflect Mtb biomass alone, so only luminescence measurements are used. Autoluminescence has been demonstrated as a proxy for Mtb cell growth (Andreu et al., 2010) and viability in response to drug treatment (Sharma et al., 2014; Zhang et al., 2012a). To benchmark changes in luminescence to changes in growth in our conditions, we performed a series of drug treatment experiments in the dormancy and intracellular models (Figure S1). Briefly, cells were treated as described above, followed by plating treated cells on 7H10 plates to enumerate colony forming units (CFU). Portions of the luminescence dose response curve that correlated with CFU changes were considered indicative of growth inhibition, and metrics derived from these portions of the curve were used for analysis.

#### Data processing and metric calculation

Data processing and dose response metric calculation were performed using custom MATLAB scripts. In brief, raw data were background-subtracted using the median of media-only wells and normalized to the mean of untreated wells within each plate. For the intracellular model, uninfected macrophages provided the background (rather than media only) for subtraction from raw data, and subsequently, data was normalized to (infected) untreated within each plate. A three-parameter Hill function was fit to each dose response (single drug or combination). Inhibitory concentrations (ICs) were calculated based on the Hill curve parameters. The area under the curve at 25% inhibition (AUC_25_) was calculated using the integral of the fit curves from 0 to the 25% inhibitory concentration (IC_25_) and normalized to the IC_25_, allowing comparisons between drug combinations. Drug interaction scores were quantified by the fractional inhibitory concentration (FIC) using Loewe additivity and Bliss independence (See Box). FICs calculated by Loewe additivity and Bliss independence were well correlated, and neither model was observed to suffer from significant bias relative to the order of the drug combination (Russ and Kishony, 2018); therefore, we proceeded to analyze drug interactions based on Loewe additivity. The growth rate inhibition (GR) metrics were calculated as previously described (Hafner et al., 2016).

#### Fitting Hill function to dose response data

We used a three-parameter Hill function where for any concentration *x* of a drug or drug combination, *Hill(x)* describes the effect at that concentration as defined

$$Hill\left(x\right)=\;\frac{E_{inf}}{1+{\left(\frac{EC50}{x}\right)}^{h}}$$

where E_inf_ describes the maximum effect achievable by a given drug or drug combination, EC_50_ describes the concentration to achieve 50% of the maximum effect, and h is the Hill slope. Data was normalized to untreated, and therefore the bottom asymptote of the Hill function was bound at 0. We found that dose response data had non-constant error variance in the media-based growth conditions, and therefore we implemented weights when we fit the Hill function to our data such that

$$Weights\;for\;Hill\left(x_{i}\right)=\;\frac{1}{stdev\left(growth\;measurement\;for\;biological\;replicates\;of\;dose\;i\right)}$$

Data points with lower variance are assigned more weight than samples with high variance when fitting. Two fitting algorithms were used: the Levenberg-Marquardt algorithm and the trust-region-reflective algorithm, each with different constraints as permitted by each algorithm. The Levenberg-Marquardt algorithm does not allow bound constraints while trust-region-reflective does; therefore, we restrict the E_inf_ to not go above 1 with the trust-region-reflective solution but cannot apply that bound to the Levenberg-Marquardt solutions. This occasionally results in a fit from Levenberg-Marquardt where the E_inf_ is much greater than 1 (i.e., 100% inhibition). As this has no biological meaning, such fits are not appropriate for our purposes, and we discarded those fits. To assess fit quality, an R^2^ was calculated; the fit from the two algorithms with the higher R^2^ was chosen.

Occasionally, the E_inf_ of the fit Hill functions was far above or below the maximum measured effect (E_max_). We categorized these dose responses into those that had a maximum effect asymptote in the normalized data or those that had no clear asymptote. Accurate representation of maximum achievable effect was important for our analysis. Therefore, we attempted to improve agreement between the E_inf_ of the fitted Hill function with the E_max_ using a custom refitting strategy. For original fits that had an E_inf_ below the E_max_, the refitting of the Hill function had the lower bound of E_inf_ parameter space constrained to within 1.25% of the E_max_. For fits that had an E_inf_ above the E_max_, the refitting of the Hill function had the upper bound of E_inf_ parameter space constrained to within 1.25% of the E_max_. Refitting with and without weights were assessed using R^2^ values. Refits with the highest R^2^ were chosen as the final fit for a given dose response curve. Additionally, the Hill coefficient during fitting had an upper bound at 10.

For all fits, the inhibitory concentration (IC) to achieve 10, 25, 50, 75, and 90% growth inhibition or kill was calculated according to the formula:

$$IC_{N}\left(drug\;x\right)=\;\frac{EC50_{drug\;x}}{\sqrt[{h_{drug\;x}}]{\frac{E_{inf,drug\;x}}{inhibition\;level}-1}}$$

The area under the curve at 25% inhibition (a measure of low potency) was calculated:

$$AUC_{25}=\;\frac{\int_{0}^{IC_{25}}Hill\left(x\right)dx}{IC_{25}}$$

#### Drug interaction quantification

DiaMOND is a tool that employs geometric optimization of the combination dose space to quantify drug interactions with fewer measurements (Cokol et al., 2017). Drug interactions are quantified by the fractional inhibitory concentration (FIC) score, which is the ratio of the observed combination dose to achieve a certain effect over the expected combination dose to achieve that same effect. An FIC < 1 is considered synergistic, FIC > 1 is antagonistic, and FIC = 1 indicates additivity. For this study, we calculated FIC scores at various growth inhibition levels. The expected dose is based on the behavior of the single drugs in the combination. We employed two null models to calculate the expected combination dose: Loewe (dose) additivity and Bliss independence (Foucquier and Guedj, 2015). Loewe additivity assumes dose additivity; that is, the effect of drugs in combination is determined by the sum of their normalized doses. By the Loewe model, the expected combination dose to achieve any inhibition level falls on the hyperplane defined by the single doses of each drug to achieve that inhibition level. The intersection of the combination dose line and the hyperplane is the expected combination dose. Bliss independence assumes response additivity; that is, drugs in combination act independently such that one cannot interfere with another. By the Bliss model, the effect of drugs in combination can be predicted by multiplying the effects of the singles, and thus the expected combination dose to achieve any inhibition level can be calculated.

FICs using Loewe additivity as a null model (above) represent the total drug interaction (Cokol et al., 2017). Total drug interaction (total FIC) is the product of the lower-order drug interactions (lower-order FIC, the recursive geometric mean of the composite lower-order drug interactions) and emergent drug interactions (emergent FIC, drug interaction properties not attributed to lower-order behaviors) (Cokol et al., 2017). The factorization of total FIC scores into lower and emergent interactions enables us to evaluate the contribution of lower-order interactions (vs. emergent behaviors) to the overall drug interaction of high-order combinations.

#### Growth rate (GR) metrics

In addition to growth inhibition at static time points, growth rate inhibition was calculated as described previously (Hafner et al., 2016). Normalized growth rate inhibition is calculated according to the formula:

$$GR\left(c\right)=2^{\frac{log_{2}\left(\frac{x\left(c\right)}{x_{0}}\right)}{log_{2}\left(\frac{x_{unt}}{x_{0}}\right)}}$$

where x(c) is the OD_600_ or luminescence readout for a given drug at concentration c at a specific time point, x_0_ is the OD_600_ or luminescence readout at time point 0 (T0), and x_unt_ is the OD_600_ or luminescence readout of the untreated population at the same specific time point. This was computed for all the concentrations in each dose response curve and then a three-parameter Hill function was fit to the data using the trust-region-reflective algorithm:

$$GR\left(c\right)=\;GR_{inf}+\frac{1-GR_{inf}}{1+{\left(\frac{c}{EC50_{GR}}\right)}^{h_{GR}}}$$

where GR_inf_ is the maximum growth rate inhibition achievable by a given drug or combination, EC50_GR_ is the dose to achieve 50% of the maximum growth rate inhibition, and h_GR_ is the Hill slope of the curve.

#### Data quality

Experiments were performed in a minimum of biological triplicate. Comparisons of data between plates and between experimental days required data quality control assessment. Each dose response was assigned a quality score that takes into account the overall quality of the data from a plate, the quality of fit of the Hill function, the single drug dose responsiveness from an experiment, and in the case of drug combinations, the equipotency in the drug combination dose responses. In brief, plate data quality was assessed with a Z’-score using multiple untreated (negative) and complete inhibition treatment (positive) wells in each plate. The fitting of the Hill function was assessed by the coefficient of determination (R^2^) of the fit as well as the closeness of the E_inf_ for each fit to the maximum observed effect for each dose response curve. Drug combination equipotency was assessed by comparing the proportional combinations normalized to their respective MICs and the idealized combination of drugs if they were perfectly equipotent. Dose responses with poor quality scores were excluded from further analysis.

#### *Z*’ calculation

To determine which conditions showed reproducible drug responses, we calculated a Z’ score. A Z’ score was calculated by the formula

$$Z^{\prime }=1-\frac{3\left(\sigma_{pos}+\sigma_{neg}\right)}{\left|\mu_{pos}-\mu_{neg}\right|}$$

where σ is the standard deviation and μ is the mean of the positive (pos) and negative (neg) controls, respectively, of those populations. We used the Z’ to assess *in vitro* model reproducibility and for in-plate quality control.

#### Data quality and processing

Several measures were taken to ensure high quality measurements for this dataset. Every plate contained untreated bacteria and in-plate standards (specified drug at the IC_90_ as determined from dose centering) as follows: for butyrate, cholesterol, acidic, standard, and valerate conditions: isoniazid and linezolid; for cholesterol-high: isoniazid; for intracellular and dormancy: moxifloxacin. Each dose response curve was assigned a quality control score that took into account the quality of growth and drug treatment within a plate (Z’ score), the success of capturing the dose response range (dose space score), the quality of the fit of the Hill function to the data (E_inf_ score and R^2^ score), and the equipotency of drug combinations (angle score).

A Z’ score was calculated for every plate to measure separation of strong positives from untreated in each experiment (See Z’ calculation section). If the Z’ score of a plate was less than 0.3, the plate was assigned a score of 2. If the Z’ score was between 0.3 and 0.5 the plate score was 1. Plates with Z’ score greater than 0.5, indicating that there was moderate discriminatory power between untreated and maximum treated wells, were assigned a plate score of 0.

To assess the quality of dose response range for a drug or combination, the number of data points collected for that drug/combination that fell between 10% and 90% inhibition was quantified. If this number was greater than or equal to 3, the “dose space score” assigned was 0. If the number was 0, the dose space score assigned was 2. If the number fell between 0 and 3, the dose space score assigned was 1.

To assess the quality of fit of the Hill function, two measures were quantified: an “E_inf_ score” and the “R^2^ score.” The E_inf_ score assessed how the E_inf_ compared to the effect at the highest tested dose of a drug or combination (E_max_). If the absolute value difference between the E_inf_ and E_max_ is greater than 0.1, the assigned E_inf_ score is 2. If the absolute value difference is between 0.05 and 0.1, the assigned E_inf_ score is 1. Below 0.05 was assigned 0. For each fit, an associated R^2^ was also calculated. If the R^2^ was < 0.7, the R^2^ score assigned was 2. If the R^2^ fell between 0.7 and 0.9, the R^2^ score was 1. Greater than 0.9 was assigned 0.

To assess the equipotency of drugs in combination dose responses (an important consideration for DiaMOND calculations), an “angle score” was calculated for combinations. This score measured the difference between the true diagonal measured (the combination of N drugs in an N-way combination) and the ideal diagonal if every drug in that combination were precisely centered around the IC_90_. If the difference between the angles (in degrees) was greater than 22.5, the angle score was 2. Between 10 and 22.5 received a score of 1, and less than 10 received a score of 0. All these scores were combined to compute a “composite score” for every single drug and combination. For single drugs, the composite score was calculated by:

$$Composite\;score\;\left(single\right)=\;\frac{1}{3}\left(Plate\;Score\right)+\frac{1}{3}\left(Dose\;Space\;Score\right)+\frac{1}{6}\left(E_{inf}\;Score+\;R^{2}\;Score\right)$$

For drug combinations, the composite score took into account the data quality of the underlying singles and the combination itself, where the underlying single score for each single drug in an N-way combination was calculated by:

$$Underlying\;single\;score=\;\frac{1}{2}\left(Plate\;Score\right)+\frac{1}{4}\left(E_{inf}\;Score+\;R^{2}\;Score\right)$$

and the resulting combination score was calculated by:

$$Composite\;score\;\left(combination\right)=\;\frac{2}{3}\left(\frac{1}{3}Plate\;Score+\frac{1}{3}E_{inf}\;Score+\frac{1}{6}\left(R^{2}\;Score+Angle\;Score\right)\right)+\frac{1}{3*N}\sum Underlying\;single\;scores$$

The composite score ranges between 0 and 2, where 0 is optimal and 2 is poor. Drugs or combinations with a composite score greater than or equal to 1 were rejected for further analysis. In addition, all fit Hill functions, and raw data for all single drugs in every experiment were checked manually; drugs that behaved unexpectedly or where the IC_90_ was below dose 5, at or above dose 10 of the dose response were removed along with all combinations that contained that drug.

#### Computational analyses

Biological replicate dose response and drug interaction data passing quality control were averaged. Means of replicate data were used for all downstream analyses unless noted. Hierarchical clustering was performed using cosine distance, and heatmaps with complete linkage dendrograms were generated using MATLAB. Other data preparation and visualizations were performed in R using the tidyverse environment packages (v1.3.0) and ggplot2 (v3.3.0) and ggpubr (v0.3.0) packages for visualization. Data table import and export were performed in R using the openxlsx (v4.1.4) and readxls (v1.3.1)packages

PCA was performed in R using the prcomp function from the stats package with each feature scaled to have unit variance before PCA. Some features were missing data; e.g., FIC_90_ metrics were missing because single drugs did not achieve IC_90_. Features with more than 35% missing data points were excluded from PCA. The remaining missing values were imputed using the mean of the corresponding input features (mean imputation) (Dray and Josse, 2015). Horn’s parallel analysis (Horn, 1965) was used to determine the number of PCs that capture more variance than expected by chance; the analysis was carried out by using the paran package (v1.5.2) in R

Machine learning was performed in R. The machine learning in R (mlr v2.17.0) package was used for all machine learning tasks involving projections of the original features onto the principal component (PC) space as the input features and drug combination outcome (C0 or C1) as labels.

#### Feature selection, feature number optimization, and model validation

The Kruskal-Wallis test was used to rank order the PC input features for ML based on the ability to discriminate outcome classes C0 and C1. As there were a limited number of drug combinations, we aimed to reduce the number of features used in the model. A Monte-Carlo resampling strategy was used to split the training data into 70/30% training/test partitions, to which we applied grid search to find the number of features that produced the largest test AUC. This feature number optimization was repeated five times for each training set, and the smallest feature set from the five iterations was chosen as the final training feature set. Models were trained on the full set of training data, and performance on new data was estimated using standard 5-fold cross validation. Validation was performed by projecting new data onto the PC space used for the model training and testing model classification performance.

#### Machine learner packages

Upon feature selection, machine learning algorithms were compared using standard 5-fold cross validation. The performance was evaluated using the AUC and the F-score (F1). The mlr package made possible on-demand loading of learners from other R packages, including Bayesian additive regression tree (bartMachine, v1.2.5.1), random forest (randomForestSRC, v2.9.3), extreme gradient boosting (xgboost, v1.1.1.1), logistic regression (stats), naive bayes (e1071, v1.7-3), support vector machine (e1071, v1.7-3), and weighted k-nearest neighbors (kknn, v1.3.1).

#### Drug overlap between training and test sets

For each drug combination with *in vivo* classification, a training set was composed from combinations that share a specific number of drugs (one, two, or three) in common with the test combination. Each training set was used to train a model to distinguish C1 and C0 outcome labels, which was subsequently applied to compute the probability that the corresponding test combination belongs to the C1 class. For a given drug overlap (one, two, or three), the C1 probability of all test combinations were then rank ordered and true positive rate (recall), false positives rate, and positive predictive value (precision) were calculated. Aggregate ROC and PR curves were constructed from the C1 probabilities of all test combinations, followed by computing of AUC and F1 metrics.

#### “Leave-one-drug-out” analysis

For each of the ten drugs in the DiaMOND compendium, drug combinations containing that drug and *in vivo* outcome annotations were set aside for validation, and the remaining drug combinations were used for model training. Performance on new data was estimated using standard 3-fold cross validation.

### Quantification and statistical analysis

Differences between outcome class groups for DiaMOND features or PCs were assessed by means (IC_90_ averages), medians (class comparisons), and standard deviation of drug combinations from each outcome group in each *in vitro* model. Because data normality could not easily be assessed with small numbers of drug combinations in each group, the Wilcoxon rank-sum test was used to compare outcome group means for statistical significance. Student’s t-tests were used for testing hypotheses of differences between model performance distributions. The hypothesis that Loewe and Bliss interaction (FIC) scores were correlated was tested using Pearson correlation, with the corresponding p-value computed empirically by randomly permuting values 20,000 times. Comparison of *in vitro* model log_2_(FIC) means was performed using one-sided t-tests with additivity (0) as the comparator. Statistical analyses were performed using the stats, ggpubr (v0.3.0), rstatrix (v0.5.0) and wPerm (v1.0.1) packages in R. The level of statistical significance is chosen to be 0.05, unless otherwise indicated in the manuscript.

## Data Availability

• All data reported in this paper are present within the published figures and publicly available in the supplemental information. Additionally, the data cube and IC_90_s have been deposited at Mendeley and are publicly available at https://doi.org/10.17632/m2y7jpz4wz.1. • Original code used for machine learning is available in this paper’s supplemental information. • The scripts used to generate the figures reported in this paper are available in this paper’s supplemental information. • Any additional information required to reproduce this work is available from the Lead Contact.

## Supporting citations

The following references appear in the supplemental information: Ammerman et al., 2018, Andries et al., 2010, Clemens et al., 2019, Cynamon et al., 2007, Fattorini et al., 2003, Lanoix et al., 2016c, Lee et al., 2017, Lee et al., 2018, Li et al., 2017, Mourik et al., 2018, Nikonenko et al., 2007, Nuermberger et al., 2006, Robertson et al., 2017, Rosenthal et al., 2008, Saini et al., 2019, Tasneen et al., 2015, Tyagi et al., 2015, Williams et al., 2012, Williams et al., 2009a, Williams et al., 2009b.

## Supplemental information

Document S1. Figures S1–S7 and Tables S1–S3

Data S1. DiaMOND compendium data cube

Data S2. Classification of drug combinations with *in vivo* outcomes Microsoft Excel workbook with single letter combination abbreviation along with drug number, classified outcome score for *in vivo* outcome (RMM, BMM, or BHeb), and literature reference. Each *in vivo* outcome is a separate tab in the workbook.

Data S3. PC class separation and feature contributions for RMM and BHeB outcomes Microsoft Excel workbook with C1 and C0 combinations compared for each PC separately using the Wilcoxon rank sum test and percent contribution to each PC of each DiaMOND compendium feature. PCA transformed labeled (by RMM or BHeB) data were used. Wilcoxon test statistics and p values and PC contribution for each *in vivo* outcome are each a separate tab in the workbook.

Data S4. Combined ML modeling input and results files and scripts for figure generation The compressed file includes, (1) Modeling files; (2) Model performance files; (3) 3 model consensus; (4) Figure panel scripts and input files. Modeling files are Microsoft Excel workbook ML input files and corresponding R scripts for running *in vitro* model subset ML model training and testing for each outcome (RMM, BMM, and BHeB). Model performance files are Microsoft Excel workbooks of systematic *in vitro* model subset classifier performance measurements for each outcome (RMM, BMM, BHeB) with training and test performance for all *in vitro* model subsets classifiers listed as area under the ROC curve (AUC) and F1(harmonic mean of precision and recall). If no true or false positives are identified, then the F1 cannot be calculated (ND, not determined). All *in vitro* model highlighted in bold and gray. Consensus prediction file is Microsoft Excel Workbook of RMM model classifier prediction probabilities. Probability of classification as C1 for all compendium (one-, two-, three-drug combinations) and validation (higher- order, and new drug) drug combination sets (Dataset). A combination single letter abbreviation is listed. Probability of C1 classification for each of the top three classifiers using simple in vitro models (ac, acidic + cholesterol; acsv, acidic + cholesterol + standard + valerate; bcsv, butyrate + cholesterol + standard + valerate, Figure 4C; Data S3) are listed as columns. Discordance between the models is indicated (Discordance, x). Combination outcome from literature listed (Class). Arithmetic mean of classifier probabilities (Consensus) are colored according to the consensus predicted class (blue > 50% and red < 50%). Combinations with incorrect consensus classification are indicated (Consensus Incorrect, x). Figure panel scripts and input files are MATLAB, R scripts, and associated workspace files that can generate plots from main Figures 1, 2, 3, 4, and 5 in paper. Scripts and input files are organized into sub folders where each folder corresponds to the corresponding figure and panel in the main text of the paper.

Document S2. Transparent peer review records for Larkins-Ford et al.

Document S3. Article plus supplemental information

## Footnotes

Supplemental information can be found online at https://doi.org/10.1016/j.cels.2021.08.004.
