# Supplementary material for: Systematic measurement of combination-drug landscapes to predict in vivo treatment outcomes for tuberculosis
Source: Cell Syst. 2021 Nov 17;12(11):1046–1063.e7. doi: 10.1016/j.cels.2021.08.004 (PMC8617591; doi:10.1016/j.cels.2021.08.004)
Supplement: Document S1. Figures S1–S7 and Tables S1–S3 [file mmc1.pdf]

Cell Systems, Volume *12*

Supplemental information

Systematic measurement of combination-drug landscapes to predict *in vivo* treatment outcomes for tuberculosis

Jonah Larkins-Ford, Talia Greenstein, Nhi Van, Yonatan N. Degefu, Michaela C. Olson, Artem Sokolov, and Bree B. Aldridge

Supplemental Information:

Figure S1.

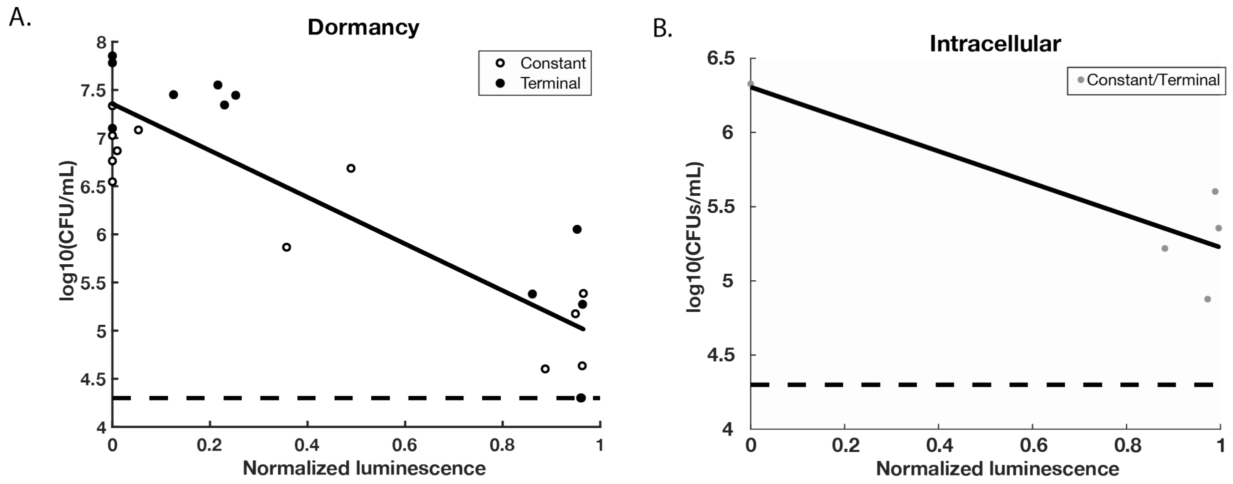

Figure S1. Benchmarking cell viability with luminescence measurements. Normalized luminescence inhibition is compared with the resulting CFU/mL for **(A)** dormancy or **(B)** intracellular *in vitro* conditions. Cells were untreated or treated with drugs as in main experiments. At indicated times (**(A)** constant or terminal, or **(B)** constant/terminal), luminescence was measured, cells were removed from multiwell plates, diluted and CFU enumerated. Luminescence was normalized to untreated as described in STAR Methods. Dashed line indicates the limit of detection for CFU/mL. Solid line indicates linear regression line ((A) r = -0.89, p-value = 8.9×10^{-9}, (B) r = -0.86, p-value = 0.06, using Pearson correlation).

Figure S2.

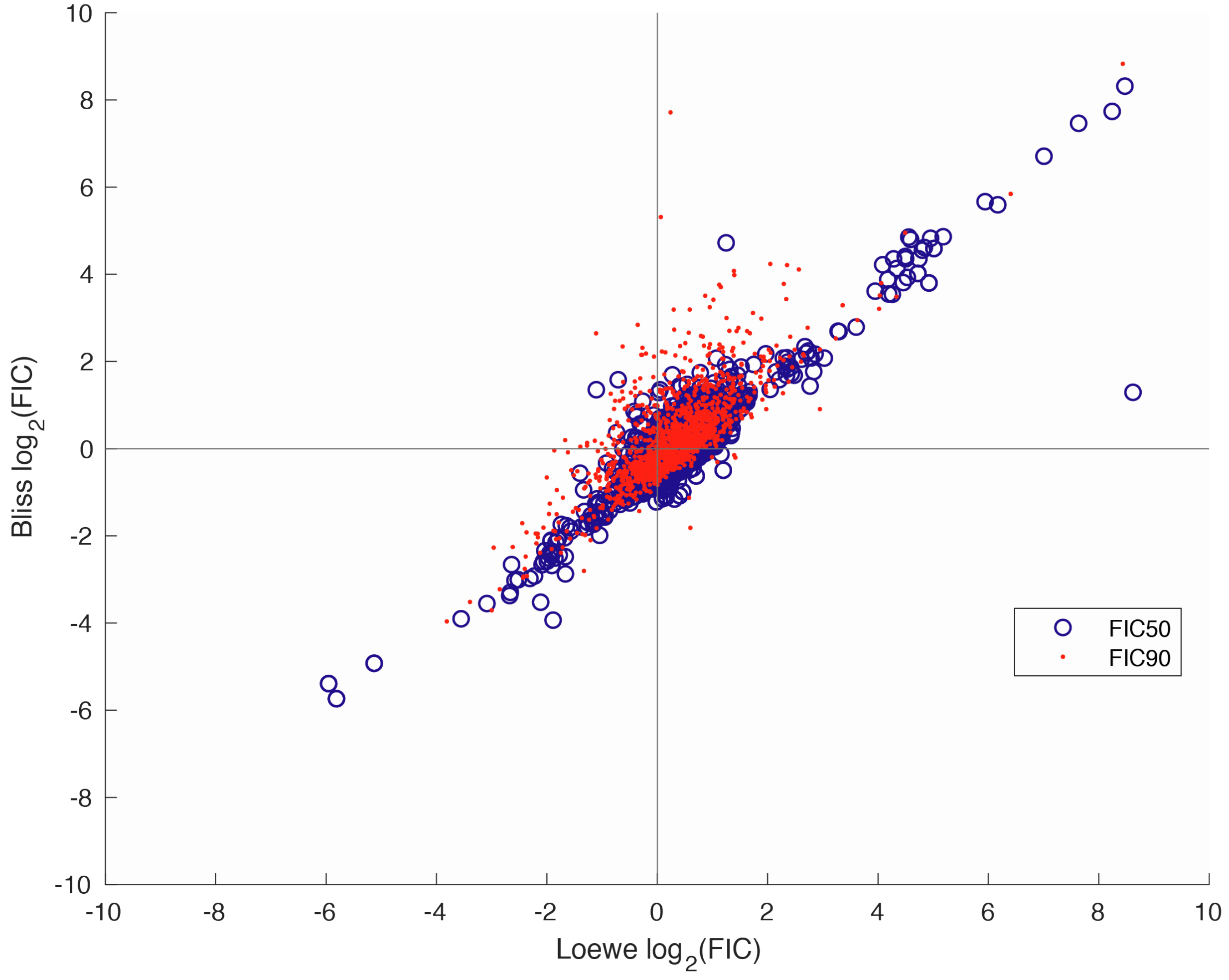

Figure S2. Comparing null reference models for drug interaction scoring. The $\log_2(FIC_{50})$ and $\log_2(FIC_{90})$ scores calculated using either the Bliss independence or the Loewe additivity null models for each DiaMOND compendium 2- and 3-drug combination at the constant and terminal time points are compared using Pearson correlation, r=0.81, empirical p = 0.0001, permutation test with 20,000 permutations).

Figure S3.

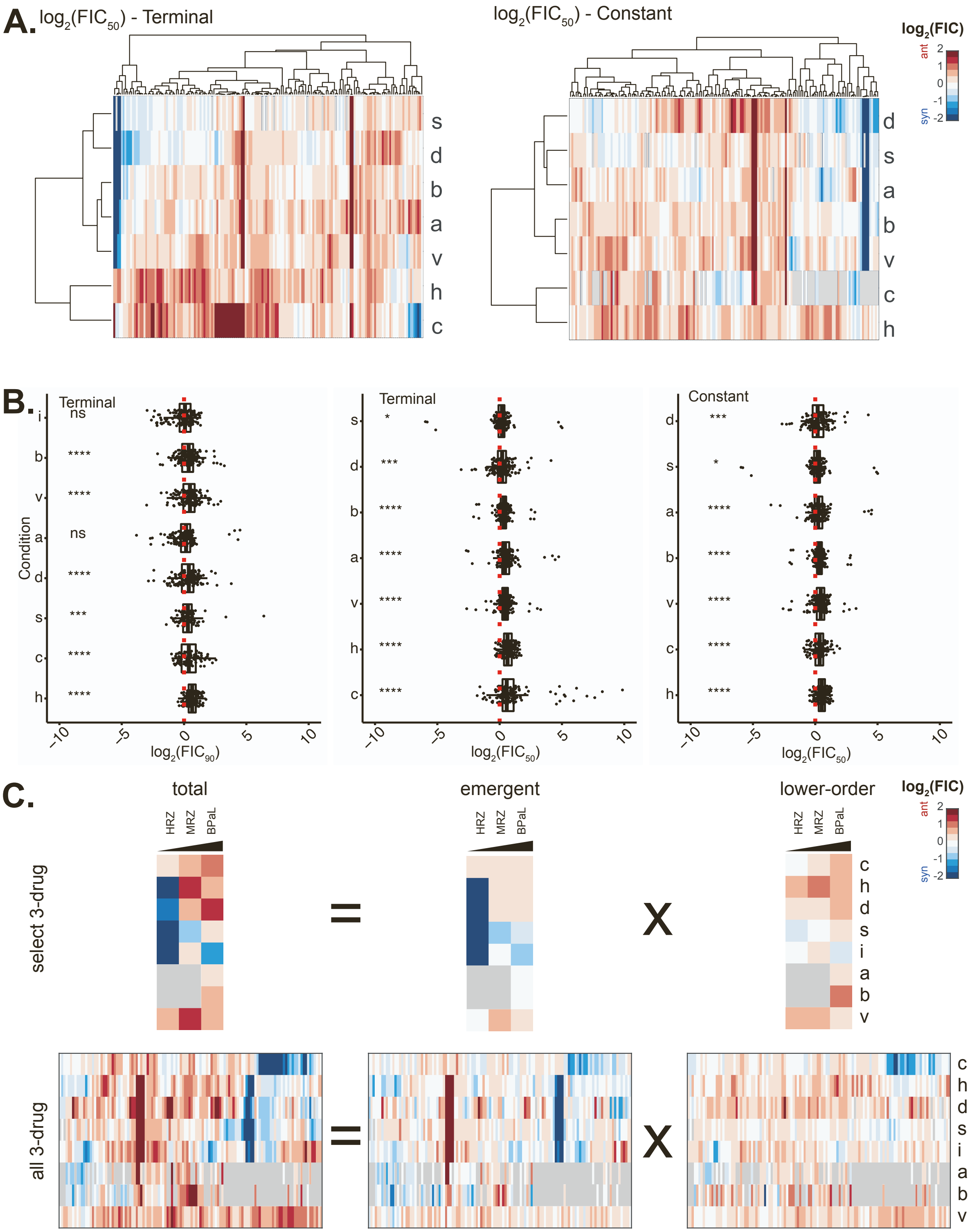
A.
log2(FIC50) - Terminal
log2(FIC50) - Constant
log2(FIC)
ant
syn
2
1
0
-1
-2
s
d
b
a
v
h
c
d
s
a
b
v
c
h
B.
Terminal
Terminal
Constant
Condition
ns
****
***
*
log2(FIC90)
log2(FIC50)
log2(FIC50)
-10
-5
0
5
10
C.
total
emergent
lower-order
HRZ
MRZ
BPaL
select 3-drug
all 3-drug
=
X
c
h
d
s
i
a
b
v

Figure S3. Intermediate potencies and component drug interaction profiles. Drug interaction profiles of each DiaMOND compendium 2- and 3-drug combination among the 10-drugs in the compendium across the *in vitro* models ($log_2(FIC_{50})$) at the **(A)** terminal time point (left), and the constant time point (right). Clusters are based on cosine distance with complete linkage as described in Figure 2. *in vitro* model order is as shown in Figure 2A and 2B. *in vitro* model abbreviations are as in Table 1 (a = acidic, b = butyrate, c = cholesterol(0.2mM), d = dormancy, h = cholesterol-high(0.2mM), i = intracellular, s = standard, v = valerate). **(B)** Box and whisker plots of drug interaction scores for each *in vitro* model are shown for $log_2(FIC_{90})$ at the terminal time point (left), $log_2(FIC_{50})$ at the terminal time point (middle), and $log_2(FIC_{50})$ at the constant time point (right). Mean $log_2(FIC)$ difference from 0 (additivity, dashed red line) is indicated to the left of each box (one-sample t-test, mu=0: **** $p<0.001$, *** $p<0.005$. ** $p<0.01$. * $p<0.05$. ns $p>0.05$, corrected for multiple hypothesis testing). **(C)** Total (left), emergent (center) and lower-order (right) $log_2(FIC_{90})$ at the terminal time point for select three drug combinations (top) and all three drug combinations (bottom) in the DiaMOND compendium. Drug combinations were hierarchically clustered using total drug interaction based on cosine distance and average linkage. *in vitro* model order and select drug combination order is as shown in Figure 2A and 2B. Drug combination abbreviations are as in Table 1 (RZ = rifampicin+pyrazinamide, HRZ = isoniazid+rifampicin+pyrazinamide, MRZ = moxifloxacin+rifampicin+pyrazinamide, BPaL = bedaquiline+pretomanid+linezolid).

Figure S4.

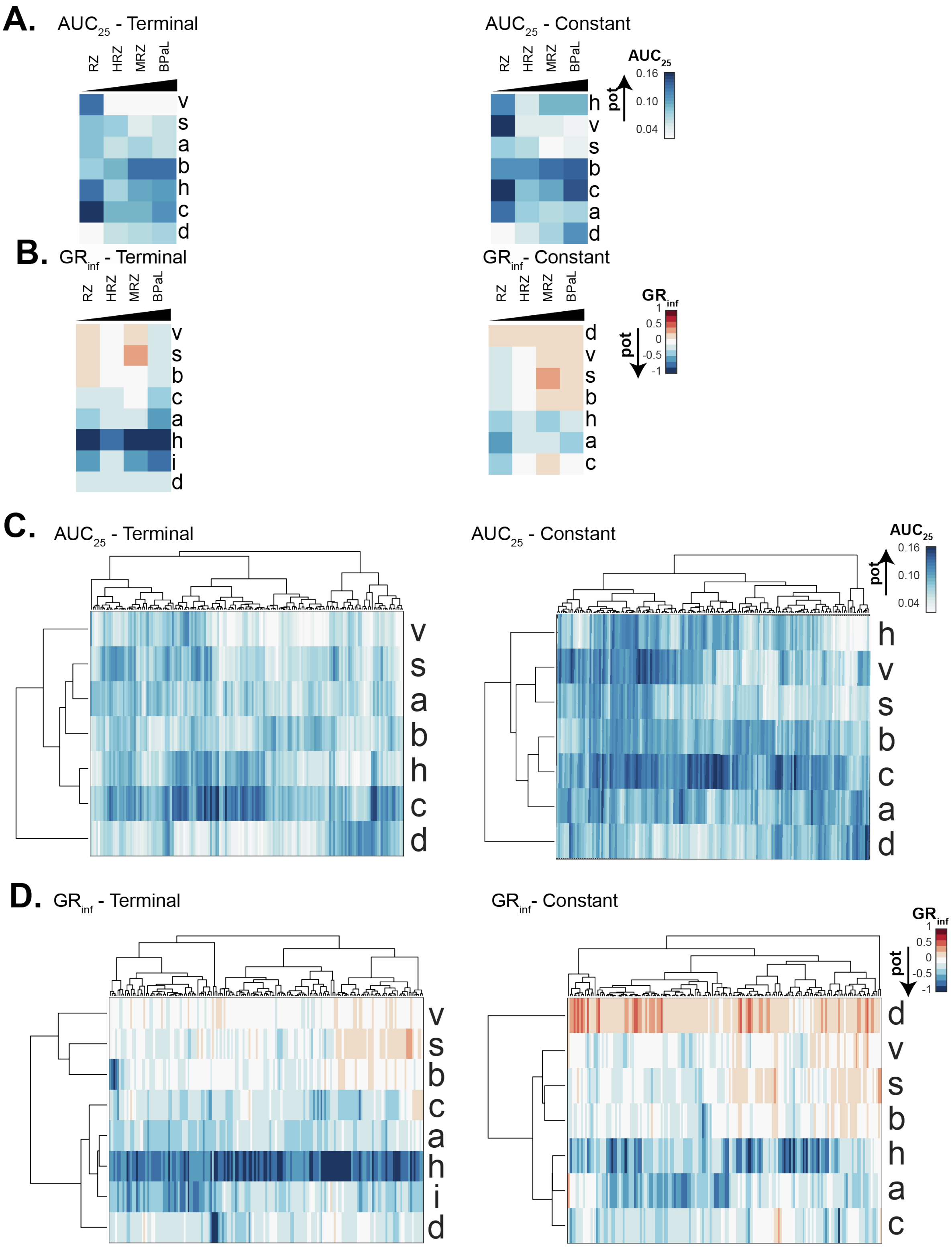

A. AUC25 - Terminal
RZ HRZ MRZ BPaL
v s a b h c d
AUC25 - Constant
RZ HRZ MRZ BPaL
h v s b c a d
AUC25
0.16
0.10
0.04
pot
B. GRinf - Terminal
RZ HRZ MRZ BPaL
v s b c a h i d
GRinf - Constant
RZ HRZ MRZ BPaL
d v s b h a c
GRinf
1
0.5
0
-0.5
-1
pot
C. AUC25 - Terminal
v s a b h c d
AUC25 - Constant
h v s b c a d
AUC25
0.16
0.10
0.04
pot
D. GRinf - Terminal
v s b c a h i d
GRinf - Constant
d v s b h a c
GRinf
1
0.5
0
-0.5
-1
pot

Figure S4. Alternative potency metric profiles for DiaMOND compendium. Profiles of drug combinations for AUC_{25} **(A and C)** and GR_{inf} **(C and D)** at the terminal (left) and constant (right) time points. **(A and B)** Selected drug combinations are ordered by relapse outcome efficacy as in Figure 2 (RZ is least and BPaL is most effective in group). **(C and D)** Profiles of all 2- and 3-drug combination in the DiaMOND. Clusters are based on cosine distance with complete linkage as described in Figure 2. Drug combination abbreviations as in Table 1 (RZ = rifampicin+pyrazinamide, HRZ = isoniazid+rifampicin+pyrazinamide, MRZ = moxifloxacin+rifampicin+pyrazinamide, BPaL = bedaquiline+pretomanid+linezolid). *in vitro* model abbreviations as in Table 1 (a = acidic, b = butyrate, c = cholesterol(0.2mM), d = dormancy, h = cholesterol-high(0.2mM), i = intracellular, s = standard, v = valerate).

Figure S5.

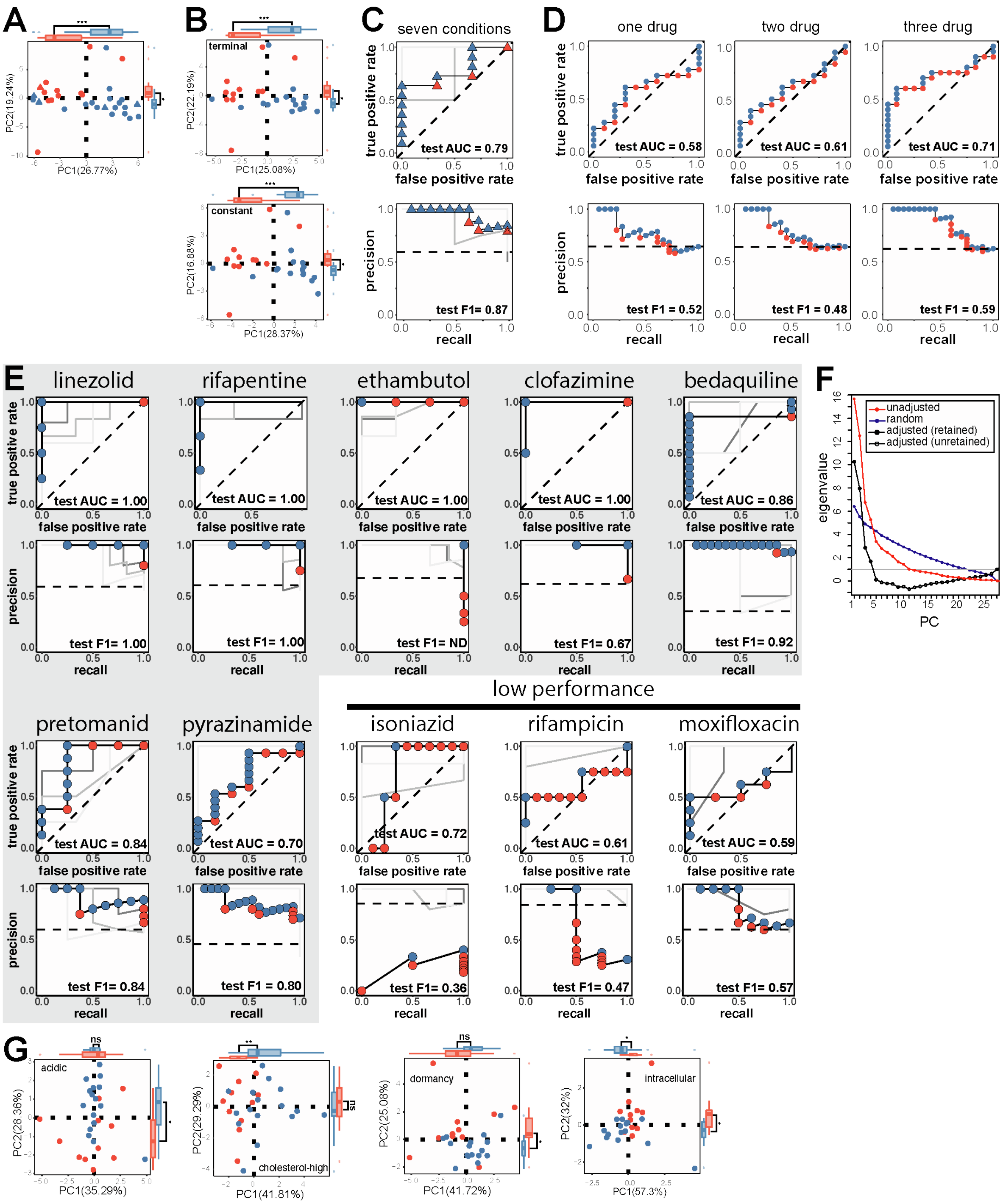

Figure S5. Separation of RMM annotated drug combinations by PCA and ML. **(A)** Projections of the DiaMOND compendium data from all *in vitro* models using two-, three-, four-, and five-

drug combinations with RMM outcome classifications (labeled as in Figure 3A) onto the first two PCs and a highlight of the percent variance explained by each PC. Outer box and whisker plots show the distributions of C1 and C0 combinations along PC1 and PC2 (Wilcoxon rank test: *** p<0.005. ** p<0.01. * p<0.05. ns p>0.05). Points are colored by class (red = C0, blue = C1) with the marker shape designating the number of drugs in a combination (circle = two-, three-drug combinations. Triangle = four-, five-drug combinations). **(B)** Projections of DiaMOND compendium data from all *in vitro* models at either the terminal (left) or constant(right) time points only using two- and three-drug combinations onto the first two principal components. Outer box and whisker plots show the distributions of C1 and C0 combinations along PC1 and PC2 (Wilcoxon rank test: *** p<0.005. ** p<0.01. * p<0.05. ns p>0.05).Points are colored by RMM outcome classifications, with percent variance explained by each PC shown as in panels (A). **(C)** ROC (top) and PR (bottom) curves for RF classifiers trained with seven of the eight in vitro models (omitting intracellular) with AUC and F1 validation performance metrics indicated in the bottom-right corner of each panel. Labeled as in Figure 3C. **(D)** Aggregate ROC (top) and PR (bottom) curves from model evaluation indicating RF classifier performance for drug combinations with specified overlap (one-drug, left; two-drug, middle; three-drug, right) between drug combinations in the validation set and those used for model training. Curves were constructed using the predicted probability for each validation combination, with the corresponding and AUC and F1 metrics indicated in the bottom-right corner of each panel. Combination are colored as in (A). **(E)** ROC (top) and PR (bottom) curves for RF classifiers with combinations containing one drug withheld for model evaluation, with AUC and F1 validation performance metrics indicated in the bottom-right corner of each panel. Labeled as in Figure 3C. **(F)** Horn’s parallel analysis showing the eigenvalues associated with each principal component,

computed using all *in vitro* models, all time points, and two- and three-drug combinations with known RMM outcome. Shown are unadjusted (red) and adjusted (black) eigenvalues, as well as eigenvalues associated with random permutation of the data (blue). Retained PCs (solid circles) and unretained (open circles) PCs are indicated based on whether the adjusted eigenvalues were above or below random (one), respectively. **(G)** PCA of DiaMOND compendium data from single *in vitro models* using two- and three-drug combinations with RMM outcome classifications. First two PCs and percent variance explained by each PC shown as in panels (A) and (B).

Figure S6.

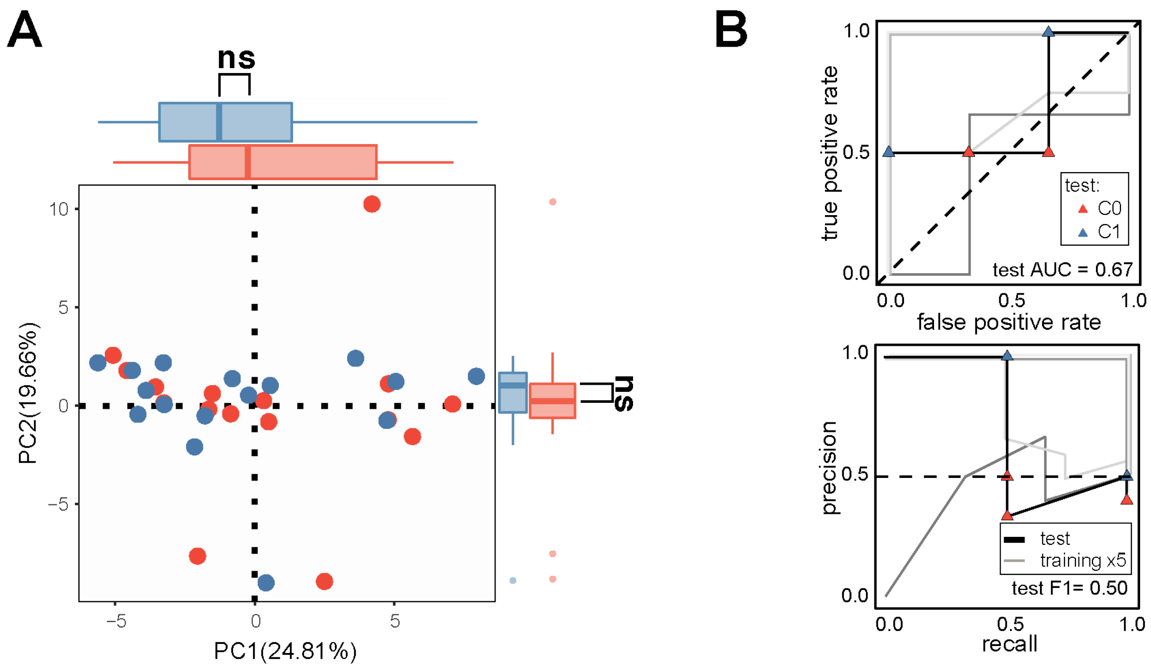

Figure S6. Separation of BMM classified combinations using PCA and ML. **(A)** Projection of DiaMOND compendium data from all *in vitro* models using two-, and three-drug combinations with BMM outcome classifications (labeled as in Figure 3A) onto the first two PCs, with the percent variance explained shown for each PC. Outer box and whisker plots show the distributions of C1 and C0 combinations along PC1 and PC2 (Wilcoxon rank test: ns p>0.05). Combinations classes are colored (red = C0, blue = C1). **(B)** ROC (top) and PR (bottom) curves of a random forest-based classifier trained on all eight *in vitro* models in the DiaMOND compendium. The model is tested with high-order combinations (4- and 5-drug combinations) that were excluded from training. Plots are labeled as in Figure 3C. Training (black) and test (grey) performances are shown with lines. Test combinations are colored by outcome class as in (A). Performance metrics are shown on plots for training and test data (Area Under the ROC

curve (AUC) and F1, harmonic mean of precision and recall). Dashed lines indicate theoretical "no-skill" model performance.

Figure S7.

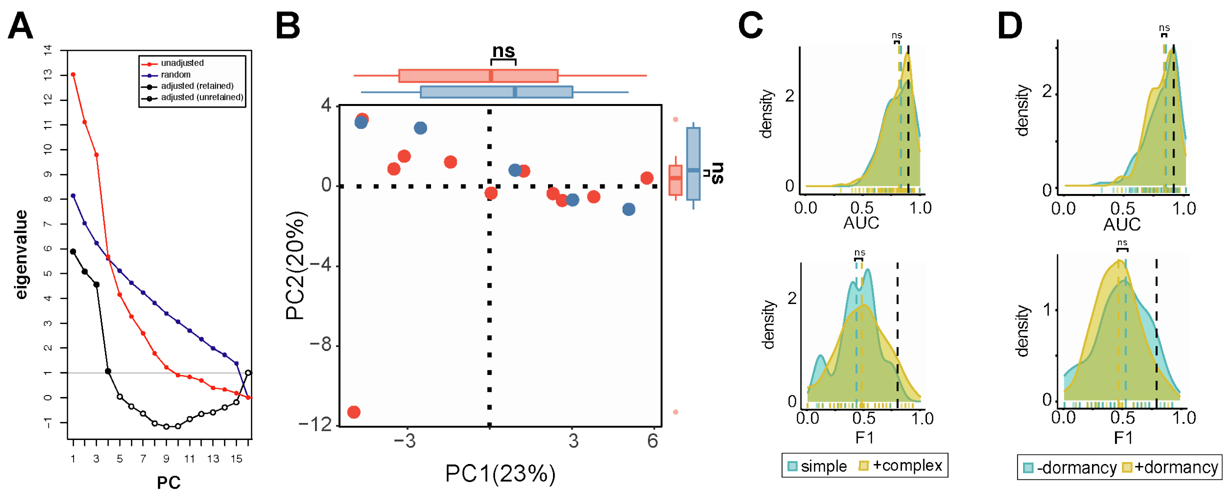

Figure S7. Separation of BHeB annotated drug combinations using PCA and ML. **(A)** Horn's parallel analysis showing the eigenvalues associated with each principal component, computed using all *in vitro* models with known BHeB outcome. Shown are unadjusted (red) and adjusted (black) eigenvalues, as well as eigenvalues associated with random permutation of the data features (blue). Retained PCs (solid circles) and unretained (open circles) PCs are indicated based on whether the adjusted eigenvalues were above or below random (one), respectively. **(B)** Projections of DiaMOND compendium data from all *in vitro* models with BHeB outcome classifications (labeled as in Figure 3A) onto the first two PCs and a highlight of the percent variance explained by each PC shown. Outer box and whisker plots show the distributions of C1 and C0 combinations along PC1 and PC2 (Wilcoxon rank test: ns $p>0.05$). Points are colored by class (red = C0, blue = C1). **(C and D)** BHeB *in vitro* model subset model performance distributions. Density distribution plots of estimated classifier performances from systematic survey of all possible *in vitro* model subsets. Distributions of ROC AUC (top) and F1 (bottom) are separated based on whether (C) technically complex models (yellow - intracellular,

cholesterol-high, dormancy) (yellow) or simple models (blue - standard, acidic, butyrate, cholesterol, valerate) or **(D)** whether dormancy is included (yellow) or not (blue). Colored dashed lines indicate the mean value for the distribution. The estimated performances when using all *in vitro* models is shown with black dashed lines. (Wilcoxon rank test: ns $p>0.05$).

Table S1.

Model	Estimated doubling time (days)	Measurement time (days)	Estimated relative doublings at each measurement
standard	0.7	2.1, 2.7, 3.4, 4.2(CT)	3, 4, 5, 6(CT)
intracellular	1.5	2, 3, 4, 5(CT)	1.3, 2, 2.8, 3.3(CT)
acidic	2	6(C), 8, 10, 12(T)	3(C), 4, 5, 6(T)
butyrate	2	6(C), 8, 10(T)	3(C), 4, 5(T)
valerate	3	9(C), 12, 15(T)	3(C), 4, 5(T)
cholesterol-high	4	12(C), 16, 20, 24(T)	3(C), 4, 5, 6(T)
cholesterol	7	7(C), 14, 21, 28(T)	1(C), 2, 3, 4(T)
dormancy	ND	2(C), 3, 4(T)	2.9(C), 4.3, 5.7(T)

Table S1. Experiment time points and estimated growth amounts for *in vitro* models. *In vitro* models, estimated doubling times, experiment measurement times, and estimated relative doubling times for each model. Constant (C), terminal (T), and constant/terminal (CT) time points were used in this study (as explained in the main text). The dormancy model had no applicable growth rate (NA). Time points for dormancy were chosen based on the standard media growth rate added for the recovery period. Day 5 was chosen for the terminal time point for the intracellular model because the uninfected J774 cells began to lift from the plates.

Table S2.

Learning algorithm	AUC	F1
Bayesian additive regression trees	0.89	0.86
random forest	0.89	0.84
extreme gradient boosting	0.73	0.75
logistic regression	0.68	0.78
naïve bayes	0.67	0.67
support vector machine	0.65	0.48
k-nearest neighbors	0.65	0.65

Table S2. Machine learning algorithm benchmarking performance metrics. PCA transformed RMM annotated 2- and 3-way data were used to compare C1 and C0 classified combinations. Area Under the ROC curve (AUC) and F1 (harmonic mean of precision and recall).

Table S3.

	Training performance		Test performance	
***in vitro* model subset**	**AUC**	**F1**	**AUC**	**F1**
all models	0.92	0.84	0.75	0.86
c	0.91	0.90	0.75	0.61
s	0.91	0.82	0.69	0.70
i	0.90	0.79	1.00	0.40
h	0.83	0.67	0.85	0.94
b	0.79	0.73	0.72	0.74
v	0.79	0.68	0.80	0.77
a	0.68	0.81	0.58	0.91
d	0.67	0.76	0.50	0.80

Table S3. RMM single *in vitro* model classifier performance. Training performance for single *in vitro* model classifiers compared with the all-*in vitro* model classifier. PCA transformed RMM annotated 2- and 3-way data were used to compare C1 and C0 classified combinations. Area Under the ROC curve (AUC) and F1(harmonic mean of precision and recall).
