# Supplementary material for: Systematic measurement of combination-drug landscapes to predict in vivo treatment outcomes for tuberculosis
Source: Cell Syst. 2021 Nov 17;12(11):1046–1063.e7. doi: 10.1016/j.cels.2021.08.004 (PMC8617591; doi:10.1016/j.cels.2021.08.004)
Supplement: Data S1. DiaMOND compendium data cube [file mmc2.pdf]

Cell Systems

Transparent Peer Review Record

Systematic measurement of combination drug landscapes to predict *in vivo* treatment outcomes for tuberculosis

Jonah Larkins-Ford, Talia Greenstein, Nhi Van, Yonatan N. Degefu, Michaela C. Olson, Artem Sokolov, Bree B. Aldridge

Summary

Initial Submission:	Received Mar. 2, 2021
	Preprint: https://doi.org/10.1101/2021.02.03.429579
	Deposited on bioRxiv, Feb. 6, 2021
	Scientific editor: Quincey Justman, Ph.D.
First round of review:	Number of reviewers: Three
	Three confidential, zero signed
	Revision invited Apr. 16, 2021
	Major changes anticipated
	Revision received Jun. 16, 2021
Second round of review:	Number of reviewers: Three
	Three original, zero new
	Three confidential, zero signed
	Accepted Aug. 4, 2021
Data freely available:	Yes
Code freely available:	Yes

This Transparent Peer Review Record is not systematically proofread, type-set, or edited. Special characters, formatting, and equations may fail to render properly. Standard procedural text within the editor's letters has been deleted for the sake of brevity, but all official correspondence specific to the manuscript has been preserved.

Editorial decision letter with reviewers' comments, first round of review

Dear Dr. Aldridge,

I'm enclosing the comments that reviewers made on your paper, which I hope you will find useful and constructive. As you'll see, they express interest in the study, but they also have a number of criticisms and suggestions. Based on these comments, it seems premature to proceed with the paper in its current form; however, if it's possible to address the concerns raised with additional experiments and/or analysis, we'd be interested in considering a revised version of the manuscript. ***We appreciate that the COVID-19 pandemic challenges and limits what you and your lab can do, so to make sure we're absolutely on the same page about the feasibility of revisions, let's schedule a Zoom call at our earliest mutual convenience.***

Before I jump into the scientific aspects of this letter, I want to be very clear that Cell Systems sees great value in this work outside of the machine learning and ability to predict. Although these are clearly appealing and important aspects of this manuscript, this rich work doesn't boil down to either of those things (note how appreciative Reviewers 2 and 3 are!). I want to mention this at the outset because Reviewer 1 raises issues that tend to be red flags in our experience. I've highlighted these in yellow. They may not prove to be in your case because the structure of the experimentation is so unique. But even if they prove very difficult to wrestle with, I want you to know two things:

1) We care deeply that the analytical work is done to a very high standard, and if that makes the conclusions "messier," then so be it. We expect some trade-off between analytical cleanliness and biological complexity, and the biology is very complex here, which we absolutely respect.

2) If, ultimately, the analytical side of this paper becomes a non-starter, we can work together to find a path forward with the rest of the paper. From my perspective, the progress made here re: elucidating the relationships between in the in vitro and in vivo data, and between drugs and conditions, is exceptionally compelling, even if it falls short of formal prediction.

With those things said, please note that as a matter of principle, I only invite a revision when I'm reasonably certain that the authors' work will align with the reviewers' concerns and produce a publishable manuscript. Treating the highlighted points with rigor and transparency amounts to make-or-break points for us, and we ask that you err on the side of rigor and transparency even it makes "punchlines" more equivocal. In addition to the concerns I've detailed above, I've highlighted portions of the reviews that strike me as particularly critical and made some notes inline.

As you address revise your manuscript, it's important that you and I stay on the same page. I'm always happy to talk, either over email or by phone, if you'd like feedback about whether your efforts are moving the manuscript in a productive direction. Please don't hesitate to reach out, as I expect this revision may be tricky. Do note that we generally consider papers through only one major round of revision, so the revised manuscript would be either accepted or rejected based on the next round of comments we receive from the reviewers. If you have any questions or concerns, please let me know. More technical

information and advice about resubmission can be found below my signature. Please read it carefully, as it can save substantial time and effort later.

I look forward to seeing your revised manuscript.

All the best,
Quincey

Quincey Justman, Ph.D.
Editor-in-Chief, Cell Systems

Reviewers' comments:

Reviewer #1: OVERVIEW

In this work, the authors aim to use in-vitro measurements of drug combinations to predict the outcome of treatments in in-vivo mouse models of tuberculosis. They measure responses of Mtb to different drugs and their combinations in eight different conditions designed to mimic relevant aspects of the environment encountered by Mtb during infection. The main approach is to use machine learning on various characteristics of drug effects measured in vitro to predict the performance of specific drug combinations in vivo. A key conclusion is that drug synergy is less predictive of in vivo outcomes than simpler measures of drug potency.

The ability to predict treatment outcomes based on in-vitro measurements of drug effects would certainly be a major advance in the field with considerable potential for improving tuberculosis treatment. Using machine learning on large datasets is a promising approach to make progress in this direction. At a more conceptual level, investigating drug interactions across different growth conditions - and, in particular, addressing if they are conserved - is an important aspect that has been largely neglected in previous work. Unfortunately, the present work has several major limitations (see below), which make it hard to fully trust the conclusions reached. Some of the conclusions are implausible and would require further validation.

DATA & CODE AVAILABILTY

For lack of time, I did not try to retrieve the data and code associated with this paper.

REQUIRED MAJOR REVISIONS

1. It is puzzling that the machine learning approach makes the worst predictions for the most direct treatment outcome (bactericidal mouse model, BMM, which measures drug effects immediately following treatment). In contrast, the relapsing mouse models (RMM), which evaluate efficacy months after

treatment - an outcome that seems much less directly connected to the in-vitro measurements - are better predicted. This does not seem plausible: How can an indirect phenomenon that occurs months after drug treatment be better predicted from the in-vitro data than the immediate effect of the drug treatment? There is a considerable risk that this is an artifact of the machine learning procedure: The outcome predicted for the test data might essentially have been included in the training data. It would be critical to explain if and how the authors can rule this out. In any case, it would be essential to add a convincing explanation for the biological plausibility of this result.

2. The value of the machine learning predictions for the 4- and 5-drug combinations (shown e.g. in Fig 3C) is limited since a large number of subsets of the drugs used is part of the training data. ***[From QJ: It's critically important to structure training vs. test properly.]*** From Fig. 3C, it seems that a total of only five drug combinations (of 4 or 5 drugs each) were used for testing, while the training set was much larger. Hence, as is, this is a way to predict a few outcomes based on many measurements, while the main challenge in the design of drug combinations is the opposite: Predictions need to be made for large numbers of combinations (combinatorial explosion) based on a much smaller number of measurements. ***[From QJ: I don't see this as an issue, although I may be wrong. I see one message of your paper as the following: in vitro measurements (which can be made in large numbers) can be used to inform in vivo experiments which must be performed small numbers. This is different from the more canonical challenge that the Reviewer is articulating. If you agree that there's a misapprehension on the reviewer's part here, note that other readers will likely have misapprehension, so please carefully craft text that could be misinterpreted.]***

A similar problem might affect the general training procedure used since the training data contains many combinations of the same drugs as the test data.

3. It seems that the drug interactions the authors find are quite variable across conditions and even determined more by the growth conditions (rows in Fig. 2 A,B) than by the drugs (columns). This is quite surprising. It would completely change the current paradigm (which is that the drugs used largely determine the drug interaction). Consequently, this result requires particularly careful validation. It should be validated in checkerboard assays that these interactions really change drastically in the different growth conditions, at least for the two-drug combinations (and the corresponding response surfaces shown in the supplement). Further, it should be noted that this variability of drug interactions across conditions alone is already a strong indication that drug interactions measured in vitro can only have limited predictive value for treatment outcomes.

4. Closely related to the previous point, all the higher-order drug combinations shown in Fig. 2B exhibit anything from strong antagonism to strong synergy depending on growth condition. This may also indicate that the definition used to classify higher-order drug interactions is inappropriate. ***[From QJ: It is critically important that the key conclusions of this work don't rest on arbitrary/fragile/narrow definitions of interaction/synergy/antagonism. The biological phenomena should be robust to conceptually minor changes/judgement calls re: these definitions.]*** It seems that only the single-drug responses were used as a reference for classifying the higher-order drug interactions (p. 9, lines 10-11). This is misleading since the drug interactions between all lower-order combinations of drugs involved should clearly be taken into account to define a sensible reference for a higher-order drug interaction. For

example, if drugs A and B are strongly synergistic, synergy is also expected for any combination of A and B with a third drug if only the single-drug responses are used as a reference. Various ways to address this issue have been proposed (see e.g. PMID 22773816 or 27974577 and other work from the same groups). Any conclusions about the prevalence of synergy or antagonism (p. 10) and the lack of correlation of synergy with in-vivo efficacy (p. 10) remain doubtful unless these conceptual issues are properly resolved.

5. The fact that ignoring part of the data ('models') in the machine learning approach often leads to better performance (p. 14, top) is confusing. It indicates that the performance of the machine learning algorithms used may be limited (since they do not detect that it would be better to just ignore part of the data). A comparison to a straightforward linear regression (which should at least not suffer from this problem) would be helpful to clarify the value of the machine learning approach.

MINOR REVISIONS, SUGGESTIONS & COMMENTS

6. Fig. 3A: The fraction of variance explained by the PCs should be shown or mentioned in the figure legend. In particular, how high is it for the 27th PC? Is the clear discrimination for the two PCs shown robust (e.g. if PCA is performed on only part of the dataset or if some weak artificial noise is added to the data)?

7. The representation of the drug interaction data in Fig. 2A is not ideal: It remains unclear which drug combinations are shown in the different columns. Since each drug is present in various different combinations, there should be a more systematic way of representing these data (e.g., for the 2-drug combinations, this is commonly done in a two-dimensional matrix, see e.g. Fig. 3 in PMID 16550172). It is clear that this is more difficult for higher-order interactions, but the authors should try to find a more informative way of showing these data than the current Fig. 2A.

8. The color scale in Fig. 1A needs to be labeled with numbers (and possibly units). According to the figure legend, this color scale shows the IC90 but high IC90s would correspond to low potency, contradicting the label on the color bar. It needs to be clarified what is shown here.

9. There is no doubt that the correlation in Fig. S2 is significant, but the p-value for the Pearson correlation (p. 9, line 17) is extremely small, which might be a result of strong assumptions underlying the statistical test used. The statistical test that was used to estimate this value should be mentioned here. A permutation test would be suitable here to estimate a more plausible p-value.

Reviewer #2: This exciting work addresses a fundamental challenge in improving therapies for tuberculosis, which rely on combination therapies. The paper builds on an approach called DiaMOND, developed by the Aldridge group in 2017, in which the universe of combination therapies is sampled in an efficient and optimized way to assess for synergy and antagonism. Here DIAMOND is applied to multiple in vitro growth conditions for M. tuberculosis that reflect the diverse microenvironments that M. tuberculosis encounters in vivo and then overlaid on fairly comprehensive compiled data from different animal models on efficacy of different regimens.

In the original work, pairwise and combination interactions were examined for nine drugs used against M. tuberculosis under standard in vitro growth conditions. But we know that during treatment of TB, one of the relevant factors is the range of microenvironments that we have to treat, and so the authors incorporate this into understanding optimal treatment regimens. Here, the authors expand on the proof-of-principle 2017 work, performing the modelling in 8 different in vitro models that capture the complex biology of TB. The DiaMOND method is highly efficient for reducing complexity and enabling assessment of drug combinations under defined conditions.

I really like the work - in concept, rigor, and extent. One of the major advances is the comprehensive integration of multiple in vitro conditions reflecting what we know about in vivo conditions into the prediction around a given regimen. One point, which the authors might be able to address (or articulate), is the added value of DiaMOND over predictions based on simpler models that would incorporate the 8 in vitro models without the array of drug combinations. The training and validation of course accounts for this and the output makes no assumptions about what will be important. But in other words, if one of the interesting conclusions that falls out is that potency is the key metric and that *antagonism* of combinations is correlated with efficacy, will it be more informative ultimately to develop, examine and measure fewer combinations under more and different in vitro conditions? ***[From QJ: Very interesting points! Note that our enthusiasm for the work is not predicated on DiaMOND, so comparing it to simpler models/methods may be a good route forward wrt Reviewer 1's comments as well.]***

The mouse outcome data across models is a fantastic compilation of studies and is used to great effect. I do think there should be some discussion of any caveats that might come from 1) combining and giving equal weight to diverse animal studies from different investigators; and 2) using the binary metric of C0 and C1 for outcome. I don't have any problem with the approach, but the authors should consider or mention any caveats.

There were honestly no technical issues at all that I could find - the work is superb.

The authors state in passing in the results section that they developed a low-oxygen multi-stress model based on the inclusion of a nitrate source for respiration in addition to butyrate as a carbon source and hypoxia. Since this is the first description of these particular conditions in the literature and is one of the conditions that they use it might be nice to expand a little on this somewhere.

Some of the references appear to be unlisted anywhere (eg Li et al 2015, Li et al 2017). Should double check.

One stylistic point, which is totally up to the authors. Some of the metaphorical jargon, particularly when combined in close succession, can be distracting - pipelines, landscapes, maps, paths - and might be used judiciously.

Reviewer #3: This well-written manuscript describes a very ambitious study in which the authors

systematically measured the Mycobacterium tuberculosis response to every 2- and 3-drug combination of 10 drugs in up to 8 different in vitro conditions intended to simulate key aspects of the various microenvironments and lesion compartments encountered by the pathogen in vivo, then used machine learning to develop classifiers predictive of combination treatment outcomes in previously published mouse model studies. The classifiers were validated using higher order (4- and 5-drug) combinations and combinations including several drugs not included in the training set.

A number of important observations are made. Firstly, the model using cholesterol as a carbon source appeared to be the single in vitro model most predictive of results in mice, but signatures including several in vitro models performed better than signatures using data from any one in vitro model. Moreover, predictors including only the technically simpler in vitro models performed as well or better than those including more complex in vitro models (ie, intracellular infection, hypoxic stress, high cholesterol concentration). This observation may pave the way to using multiple simpler in vitro models together to give a composite ("sum-of-parts") picture of combination performance across several conditions and thereby close a major gap in TB drug and regimen development by enabling confident prediction of in vivo efficacy from in vitro models. Secondly, drug potency measures were more important predictors of combination drug outcomes than interaction measures, suggesting that potent drug combinations should be prioritized for further study and should not necessarily be deprioritized if they are antagonistic in in vitro assays. There are also surprising results that will require further study to confirm and explain. For example, in vitro models had less predictive capacity for predicting in vivo outcomes based on the change in lung CFU counts in non-C3HeB/FeJ mouse strains (BMM outcomes) compared to outcomes based on relapse in non-C3HeB/FeJ mouse strains (RMM outcomes) and change in CFU in C3HeB/FeJ mice (BHeB outcomes). Other interesting observations stemming from the in vitro studies themselves include that synergy appears to be a property of both drug and growth environment rather than an intrinsic property of drugs.

Overall, the manuscript and the work described is a tour de force that is expected to have important and lasting impact on the TB drug and regimen development space. Some additional major and minor comments are provided for the authors' consideration below.

Major comments:
The observation that multiple in vitro models may combine to give a "sum-of-parts" picture of in vivo efficacy is important. Even in less complex (i.e., non-C3HeB/FeJ) mouse models, some bacilli reside in acidified phagolysosomes (for which the acidic growth model may be most relevant), while others reside in foamy macrophages (for which cholesterol and lipid carbon source models may be most relevant). This point that even the simpler in vitro models identified as most informative may be representing specific in vivo niches and bacterial sub-populations is implied in the Discussion but could be made a bit more explicitly. The similar predictive power of the best classifiers between the RMM and BHeB outcomes also suggests that the key microenvironments may be more similar in these mouse models than is currently appreciated.

There is limited diversity in the regimens tested in mice by virtue of relying on published data. Nearly all combinations (e.g., all but 2 3-drug combinations) include either a rifamycin or bedaquiline, and often pyrazinamide. This is an important caveat when assessing the value of drug interaction features for

classification. If the efficacy of rifamycin-based and bedaquiline-based regimens is highly dependent on the efficacy of those single drug components (or the combination with pyrazinamide), the observation about the limited predictive impact of drug interaction features may not be as generalizable as one may assume. This may be compounded by the challenges inherent in modeling the in vivo effects of pyrazinamide in many in vitro conditions.

The high performance of the best-performing classifiers trained on the simple in vitro models on the validation set of 4- and 5-drug combinations and additional drugs is promising but it should be acknowledged that 2 of 3 new drugs added in this validation set (delamanid and sutezolid) are from the same drug class and have the same mechanism of action as drugs in the training set. Another limitation is that the efficacy of the higher order combinations is often driven by the efficacy of lower order combinations.

The analyses focused on 1-2 time points per in vitro model, although more time points were assessed. Fig S3 seems to suggest that results using the terminal time point vs. the constant time point may be more different than one would expect. Were any sensitivity analyses considered or performed to determine whether use of different time points would have altered any results or conclusions?

It should be acknowledged in the discussion that the bacterial strain used in the in vitro studies was not the same strain used in many of the mouse studies. This is not necessarily a weakness but it is a potential limitation that may not be evident to many readers.

Was there any concern for selection of drug-resistant mutants over 7 days in the active growth model(s) that might confound the drug interaction measures?

Minor comments:
Page 4, line 1 (and elsewhere): suggest "treatment-shortening" rather than "treatment-shorting"

Page 4, last line: suggest "…including granulomas…"

Page 5, line 3: model-specific drug responses may also be attributable to different lesion structures leading to differential drug exposures in specific lesion compartments in addition to different microenvironments

Page 7, paragraph 2: The mechanism of action of clofazimine may not be well described by inhibition of energy metabolism or respiratory inhibitor (as in Table S2). What is the basis for this classification?

Page 9, 6th line from bottom: the rationale for selecting Loewe additivity over Bliss independence as the null model for systematic analysis in the compendium should be provided for the reader.

Page 23, last 10 lines: discussion of Study 31 and the PHZE prediction should also include discussion of the PHZM prediction from the DiaMOND compendium. PHZM is the first trial-confirmed 4-month regimen non-inferior to RHZE. What was the consensus prediction for PHZM from the in vitro compendium and how did that align with RMM and clinical outcomes? This section paints the predictions of RMM and

DiaMOND as dichotomous (ie, right or wrong) but in reality treatment response is continuous. The rank order of PZM > PZH > RZM > RZH in the RMM was also consistent with rank ordering from Study 31 and REMox (ie, PZMH > PZHE > RZM (H/E) > RZHE, although only PZMH was the only regimen to meet non-inferiority criteria as a 4-month regimen. Therefore, the DiaMOND compendium results may be more consistent with mouse outcomes and not "contradictory", as implied here.

Fig 1 legend: "C" is missing on the figure; "B" should be next to the curve below the cube rather than next to the cube

Page 30, paragraph 3, line 3: "To compare low dose potency…"

Page 30, second-to-last line: "We log transform FICs…"

Fig S4: why does the vertical order of the models change across the graphs? It makes it more difficult to compare results across the graphs. Is there a rationale for doing this?

Fig S5C: cholesterol is missing an "l" at the end in the right y axis.

Figs S8 and S9: there are asterisks in the figure legends but none in the graphs themselves.

Table S2: it's likely not appropriate to pigeonhole pyrazinamide as a respiration inhibitor based on studies by Thomas Dick and colleagues, nor SQ109 as strictly a cell wall synthesis inhibitor based on evidence for its effects on membrane potential and respiration.

Table S2 legend, line 2: combination should be singular, not plural?

Table S7: shouldn't HRpZE be classified as "C1" here, as discussed in the text?

Authors' response to the reviewers' first round comments

Attached.

Editorial decision letter with reviewers' comments, second round of review

Dear Bree,

I'm very pleased to let you know that the reviews of your revised manuscript are back, the peer-review

process is complete, and only a few minor, editorially-guided changes are needed to move forward towards publication.

In addition to the final comments from the reviewers, I've made some suggestions about your manuscript within the "Editorial Notes" section, below. Please consider my editorial suggestions carefully, ask any questions of me that you need, make all warranted changes, and then upload your final files into Editorial Manager. ***We hope to receive your files within 5 business days, but we recognize that the COVID-19 pandemic may challenge and limit what you can do. Please email me directly if this timing is a problem or you're facing extenuating circumstances.***

As you look forward to acceptance, please do consider submitting one of the protocols you've developed in this paper to STAR Protocols, or extending this offer to one of your trainees. STAR Protocols is geared towards trainees and its key purpose is to provide complete and consistent instructions for how to conduct reproducible experiments. If you have any questions, please email starprotocols@cell.com.

I'm looking forward to going through these last steps with you. Although we ask that our editorially-guided changes be your primary focus for the moment, you may wish to consult our FAQ (final formatting checks tab) to make the final steps to publication go more smoothly. More technical information can be found below my signature, and please let me know if you have any questions.

All the best,
Quincey

Quincey Justman, Ph.D.
Editor-in-Chief, Cell Systems

Editorial Notes

Title: Your title is clear and accurate, but I always ask authors to double-check that their titles are as effective as they can be. Note that an effective title is easily found on Pubmed and Google. A trick for thinking about titles is this: ask yourself, "How would I structure a Pubmed search to find this paper?" Put that search together and see whether it comes up is good "sister literature" for this work. If it does, feature the search terms in your title.

Abstract: I've gone over your abstract with the goals of increasing accessibility to a broad audience and making it more concrete. See what you think. Please feel free to revert anything that you don't like or that you feel distorts your meaning! I apologize if there are instances of the latter.

Lengthy multidrug chemotherapy is required to achieve a durable cure in tuberculosis. However, lack well-validated, high-throughput in vitro models that predict animal outcomes. Here, we provide an extensible approach to rationally prioritize combination therapies for testing in validated in vivo mouse models of tuberculosis. We systematically measured Mycobacterium tuberculosis response to all 2- and 3-drug combinations among ten antibiotics in eight conditions that reproduce lesion microenvironments, resulting in <number> measurements. Using these in vitro data, we developed classifiers predictive of multidrug treatment outcome in a mouse model of disease relapse and identified ensembles of in vitro models that best describe in vivo treatment outcomes. We identified signatures of potencies and drug interactions in specific in vitro models that distinguish whether drug combinations are better than the standard of care in two important preclinical mouse models. Our framework is generalizable to other difficult-to-treat diseases requiring combination therapies. A record of this paper's Transparent Peer Review process is included in the Supplemental Information.

Manuscript Text: Your text is compelling and clear, and I greatly admire the forthright, results-speak-for-themselves tone of the whole paper, and the results section especially. The paper is on the long side, but I don't want to cut any of the following sections for the sake of text length: intro, results, figure legends, STAR Methods, references. But the discussion is too long, both from a text-length perspective and from the perspective that too much text begins to erode clarity. My suggestion is to cut the following: lines 549-569, 609-642, 680-684.

Also, one important note about clarity: for a study like this, especially for a broad audience, it's very important to define in vitro and in vivo explicitly, then use them carefully, deliberately, and consistently throughout the paper. My preference would be to call cell line work "in vitro" and then use "in vivo" to refer to the mouse models (n.b. please do say "in vitro culture" and "in vivo mouse model" often, just to anchor them in your readers' minds).

Thank you!

Reviewer comments:

Reviewer #1: In their revised manuscript, the authors made a considerable effort to clarify the issues I had raised. My most important concerns were due to misunderstandings, which are largely resolved now due to the additional explanations, analysis, and discussion that was added. In particular, the potential value of these results for TB drug regimen design has become clearer and it is reassuring that key results do not depend on the machine learning approach used but are also captured by linear methods (PCA).

It is only on the third point I had raised that I am still not convinced: It seems that the drug interactions are determined more by the growth conditions (rows in Fig. 2 A,B) than by the drugs (columns). Here, the authors argue that drug efficacy depends on conditions, which I do not doubt. Neither do I doubt that drug interactions can change across conditions. The most drastic illustration of what I'm concerned about is

that, according to the data in Fig. 2 A,B, virtually *all* drug combinations become antagonistic in the cholesterol-high condition, which seems implausible (there are less drastic examples of the same issue for some of the other growth conditions). It is relatively easy to imagine conditions that change the efficacy of many drugs (e.g. if drug uptake is generally increased) but I find it much harder to see how the growth condition would change drug interactions in the same direction across the board. If the authors are sure that this is no artifact, it would help to include and discuss a potential explanation for this phenomenon. It might make sense if all drug interactions are caused by the same underlying mechanisms but that seems quite unlikely.

Reviewer #2: Exciting work! The authors have responded thoughtfully to all my concerns and suggestions.

Reviewer #3: The authors' responses to prior reviews are thoughtful and comprehensive. No further comments.

Response to Review

We thank the reviewers for their suggestions and supportive remarks about our work. We believe that the new analyses and text revisions included in response to your criticisms and questions have improved our paper and the clarity of the conclusions, and we are grateful for the thoughtful reviews. Please find below a detailed response to the concerns of each reviewer (in blue text). The figure numbers, especially for supplementary materials, have changed from the original submission to meet figure limits - we reference the new numbers below.

Reviewer #1
Major comments

1. It is puzzling that the machine learning approach makes the worst predictions for the most direct treatment outcome (bactericidal mouse model, BMM, which measures drug effects immediately following treatment). In contrast, the relapsing mouse models (RMM), which evaluate efficacy months after treatment - an outcome that seems much less directly connected to the in-vitro measurements - are better predicted. This does not seem plausible: How can an indirect phenomenon that occurs months after drug treatment be better predicted from the in-vitro data than the immediate effect of the drug treatment? There is a considerable risk that this is an artifact of the machine learning procedure: The outcome predicted for the test data might essentially have been included in the training data. It would be critical to explain if and how the authors can rule this out. In any case, it would be essential to add a convincing explanation for the biological plausibility of this result.

We thank the reviewer for raising this concern, as it made us aware that we did not adequately frame the relationships among (and uses of) the different preclinical mouse models for TB drug regimen design. Relapse is not an indirect outcome of bactericidal outcomes, but is rather a measurement of the residual of treatment (relapse) compared to how the culturable bacterial burden was reduced. For this reason, relapse is not a secondary metric to bactericial measures. In TB, this is evident in the discordance between these outcomes in animal studies and clinical trials. Bactericidal measures of drug treatment are not a strong predictor of durable treatment efficacy in humans (and this can also be observed in mice), necessitating use of relapsing models as key preclinical models (sample publications outlining the challenge of using culturable bacteria to predict long term clinical treatment outcome in TB: Dooley et al 2017 Adv Drug Del Rev (PMID=26827911), Horne et al 2013 Lancet Infectious Disease (PMID=20510279), Phillips et al 2016 BMC Medicine (PMID=26847437), Goletti et al 2018 Eur Resp J (PMID=30361242)). For example, Nuermberger et al 2006 AAC (PMID=18285479) demonstrated that three regimens (HRZ, MRZ and PaMZ) had no culturable bacteria after four months of treatment but that HRZ showed 50% relapse free outcome while MRZ and PaMZ showed 95% and 100% relapse free outcome, respectively. RMM and BMM outcomes measure different processes or bacterial niches and may require different growth conditions or metrics for informative *in vitro* study. To clarify these points and to make this work more accessible to a broader readership, we have added a new figure (new Fig. 1A) and section to the introduction to explain the different *in vivo* outcomes and why multiple models are needed to study TB drug response:

> Lines 94-106: Mtb drug response differs between these two types of mouse models, and both are important preclinical tools because the model-specific drug response is thought to result from the different lesion microenvironments present in each animal model (Figure

> 1A) and the differential drug exposure in lesion compartments is influenced by lesion structure (Driver et al., 2012, Lanoix et al., 2015b, Lenaerts et al., 2015, Nuermberger, 2017). Drug treatment efficacies are often evaluated first by directly measuring bacterial burden followed by monitoring disease relapse once treatment is completed (Figure 1A). Enumerating bacterial burden at different times during treatment is an efficient method for assessing drug treatment and is often used to eliminate treatments with moderate effects from further consideration (Tasneen et al., 2016, Li et al., 2015, De Groote et al., 2011, Gumbo et al., 2015, Nuermberger, 2008). In contrast, monitoring relapse is considered a more reliable outcome for assessing the durability of cure and is more comparable to the clinical outcome of treatment success (Gumbo et al., 2015, Nuermberger, 2008, Dooley et al., 2016).

Though we believe we have provided a plausible reasoning for why we could predict RMM but not BMM outcomes using our dataset, we have also used linear methods (PCA, unsupervised) to compare separation in signal between outcome classes for these outcome types. We observe a strong separation of *in vivo* outcome classes in RMM both in training combinations only (Fig. 3A) and in all (training and test) combinations together (Fig. S5A). Therefore, separation of classes is independent of machine learning methodology and how the data is split into training and test sets. In contrast, the classes are not separable for the BMM outcomes in the top two principal components (Fig. S6A). These analyses suggest that there is a strong signal distinguishing outcome classes in RMM, but our dataset is not capturing key metrics or culture conditions for an equivalent signal in BMM. We have revised the manuscript to explain the strong differences in ability to separate classes and included the new RMM combined training and testing PCA plot (Fig. S5A) and the updated BMM PCA plot (Fig. S6A) to the revised manuscript:

> Lines 319-329: Principal component analysis (PCA) demonstrated that linear combinations of *in vitro* features could separate C0 and C1 drug combinations containing two and three drugs without using the *in vivo* class information (Figure 3A, Wilcoxon rank-sum, $p<0.005$, Data file S2). A separation was also apparent in PC spaces of combinations with two, three and four drugs (Figure S5A). Lastly, we also observed similar class separation when PCA was repeated using data from the constant and terminal time points separately (Figure S5B), indicating that similar conclusions would be drawn from any of the time point data. Because PCA does not use class labels in its computation, the observed separation of *in vivo* classes suggests that *in vitro* measurements from the DiaMOND compendium contain strong signal that characterizes the performance of drug combinations *in vivo*.

> Lines 431-436: Using the same analysis process, we investigated whether drug combinations with different BMM outcome classes (Data file S2) were separable in the top PCs. In contrast to RMM classes, we observed no separation of BMM C0 and C1 in the top two PCs (Wilcoxon rank sum test, $p>0.05$, Figure S6A). Consistent with the lack of separation in the PC space, we found that ML classifiers could not predict C0 or C1 drug combinations for the BMM outcome (Data file S3; AUC = 0.67, F1 = 0.40; Figure S6B).

2. The value of the machine learning predictions for the 4- and 5-drug combinations (shown e.g. in Fig 3C) is limited since a large number of subsets of the drugs used is part of the training data. From Fig. 3C, it seems that a total of only five drug combinations (of 4 or 5 drugs each) were used for testing, while the training set was much larger. Hence, as is, this is a way to predict a few outcomes based on many measurements, while the main challenge in the design

of drug combinations is the opposite: Predictions need to be made for large numbers of combinations (combinatorial explosion) based on a much smaller number of measurements A similar problem might affect the general training procedure used since the training data contains many combinations of the same drugs as the test data.

We agree that it is important that we are clear about the limitations of this pipeline and how it should be used. We thank the reviewer for making such an important point - there is overlap of drugs and combination subsets between the training and test sets. In TB drug regimen design, high-order combinations are tested after there are a few combination data in preclinical animal models. Therefore the overlap in combination sets does not limit the practical use of the machine learning predictions, as this overlap occurs in the current workflow of TB regimen design (where combinations are tested iteratively). Our training/test structure is well matched to the process of drug regimen design for tuberculosis and can be used to better leverage these difficult-to-gather data. In the revised introduction, we have included more background about the process and challenges in TB drug regimen design. In the results and discussion, we build on this background to clarify how DiaMOND, predictors, and *in vivo* data should be best integrated and expanded as the field gathers more data and drug candidates.

> Lines 61-67: Despite the size of the combination space, new TB regimens are built by augmenting well-studied drug combinations with the substitution or addition of new drugs (Ginsberg and Spigelman, 2007, Kerantzas and Jacobs, 2017, Tiberi et al., 2018a, Tiberi et al., 2018b, Wallis et al., 2016, Lienhardt et al., 2019, Dooley et al., 2019), often based on the results of iterative *in vitro* and preclinical studies (Figure 1A). A Phase 3 clinical trial ("Study 31") recently demonstrated that treatment duration could be shortened by substituting two of the drugs in the standard four-drug regimen with existing TB antibiotics (Dorman et al., 2021).

> Lines 509-518: Our classifiers perform best when a drug of interest is included in some combinations in the training set. Nonetheless, we expect that our buildable approach to use systematic *in vitro* data to prioritize drug combinations for *in vivo* study will have immediate benefit. As more antibiotics with more diverse mechanisms of action are tested in animals and added to DiaMOND datasets, we anticipate that we will be able to make accurate predictions of untested combinations containing these new antibiotics. We can therefore leverage these data with more expansive DiaMOND measurement to prioritize combinations for further testing *in vivo*. Together, our study establishes a practical approach to prioritize combination therapies using economical, scalable, and expandable *in vitro* measurements while maximizing the use of *in vivo* efficacy data that are generated early in TB drug development.

We have performed additional analyses to understand the effect of drug and subset overlap between training and test sets. There are too few TB antibiotics and *in vivo* data of combination efficacies to structure the training and test sets so that there is no overlap in the mechanisms of drug action or drug subsets. Instead, we considered each test combination in isolation and generated suites of training sets that overlap with it by one, two, and three drugs. We then

measured performance as a function of the drug overlap and observed increased classification accuracy associated with larger overlap (Figure S5D). We also tested how classifiers would perform on a drug that is absent from the training set entirely; in the majority (but not all) of these "leave one drug out" tests, we find that we accurately predict outcome labels (Figure S5E). These results suggest a possible utility of our workflow in a real-world setting where combinations are tested in animals and in the clinical in a sequential manner, e.g. combinations prioritized for testing are designed from the bottom up in an *ad hoc* manner that incorates *in vivo* efficacy from single-drug and a few combinations. We discuss these analyses and implications of use-cases of these classifiers in the results and discussion:

> Lines 354-372: Because drug combination design and testing in animal models is iterative (Figure 1A), we encounter an unavoidable overlap of drugs between many of the combinations used for model training (low-order) and validation (high-order, Data file S2). We asked how much this overlap contributes to the observed classifier performance by systematically considering training/test splits with 1-, 2- and 3-drug overlaps (see STAR Methods). As expected, we found that validation performance increased with higher drug overlap between the model training and validation combinations (Figure S5D). A major goal of drug regimen design is to understand how to best use new drugs, rather than avoid overlap with previously tested combinations; for example, by substituting a new drug for an existing drug in a well-tested combination based on combination efficacies in animal models (Ginsberg and Spigelman, 2007, Kerantzas and Jacobs, 2017, Tiberi et al., 2018a, Tiberi et al., 2018b, Wallis et al., 2016, Lienhardt et al., 2019). We therefore sought to evaluate model performance on combinations containing a drug that had not been encountered by the model during training ("leave-one-drug-out"). We observed that for most (seven) drugs, the model was able to accurately predict whether the addition of that drug to a lower-order combination would result in improvement over the standard of care (Figures S5E, AUC>0.7 and F1>0.66). Taken together, these results indicate that our modeling structure is well-matched to the data generated during TB drug regimen design, where combinations are constructed iteratively so that a few combinations with new antibiotics of interest are tested early for efficacies in animal models.

The reviewer also correctly points out that the test performance in Figure 3C is computed using only five data points, which raises the question of how well this estimate holds up when the predictor is applied to additional data. To increase the power of the estimate, we relaxed the requirement that a drug combination must have data across all eight conditions. Specifically, we repeated the analysis in Figure 3C on 14 combinations with data in 7 of 8 conditions (excluding the intracellular model, because we had fewer measurements in that model) and included results as a new Supplementary Figure S5C. We observe that the performance estimate computed on a higher number of data points is comparable to the one in Figure 3C, suggesting that the predictor accuracy will generalize to new data. This critique also highlights that we did not do an adequate job explaining the process of drug regimen design for TB - the nature of the work is that very few combinations will be tested in animals and in the clinic. Our goal is

therefore to leverage the practical ability to make large numbers of *in vitro* measurements to prioritize the few combinations to be tested *in vivo*. We have described this motivation more clearly in the revision.

> Lines 138-140: Overall, our study establishes a logistical path to optimize combination therapies for TB that is consistent with current regimen design strategies and uses systematic measurement in validated *in vitro* growth models and computational modeling.
>
> Lines 126-128: Our long-term goal is to extensively search the drug combination space empirically using practical *in vitro* measurement to prioritize combinations to be tested in preclinical animal models.
>
> Lines 346-353: We noted that for validating the RMM classifier, there were five higher-order combinations for which we had DiaMOND data in all eight conditions (Figure 3C). To determine whether the five test combinations of the eight-condition model represented a generalized measure of performance, we retrained a classifier using seven conditions (all but the intracellular model) from which we had more test data available (14 combinations). We observed similarly high validation performance in the seven-condition model (Figure S5C, AUC=0.79, F1=0.87), suggesting that the performance of the eight-condition model is not simply an artifact of the small test set.

3. It seems that the drug interactions the authors find are quite variable across conditions and even determined more by the growth conditions (rows in Fig. 2 A,B) than by the drugs (columns). This is quite surprising. It would completely change the current paradigm (which is that the drugs used largely determine the drug interaction). Consequently, this result requires particularly careful validation. It should be validated in checkerboard assays that these interactions really change drastically in the different growth conditions, at least for the two-drug combinations (and the corresponding response surfaces shown in the supplement). Further, it should be noted that this variability of drug interactions across conditions alone is already a strong indication that drug interactions measured in vitro can only have limited predictive value for treatment outcomes.

Though we have demonstrated condition-specific drug potency and interaction effects at a large scale in our study, ours is not the first study to observe condition-specific effects on drug interactions measurements (Belanger et al., 2020 (PMID=33318204), Sanders et al., 2018 (PMID=29771999), Dillon et al., 2019 (PMID=31353294), Cokol et al., 2019 ACS Chem Biol (PMID=30596642)). Indeed, the strong dependence on drug efficacy on the microenvironment for Mtb is very well described. For example, pyrazinamide is known to be ineffective in rich growth conditions but is potent in acidic growth conditions *in vitro*. This effect has been demonstrated *in vivo*, and has motivated the parallel use of multiple animal models (including expansion of mouse models from BALB/c to C3HeB/FeJ mouse models) (Irwin et al 2016 ACS Infect Dis (PMID=27227164), Lanoix et al 2016 AAC (PMID=26643352), Lanoix et al 2015 Dis Model Mech (PMID=26035868), Lanoix et al 2016 AAC (PMID=26574016)). Similarly, bedaquiline and isoniazid efficacies are dependent on the growth environment (Smith et al 2020 PNAS (PMID=32680963), Koul et al 2014 Nat Commun (PMID=24569628), Lee et al Nat

Commun (PMID=31266959), Koul et al 2008 J Biol Chem (PMID=R207910), Betts et al 2002, Mol Micro (PMID=11929527))

We have previously published validation of DiaMOND to approximate checkerboard assays and to measure the null-effect (using sham combinations of drugs in combinations with themselves) (Cokol et al, 2017 Sci Adv (PMID=29026882)). When we designed our study, we noted that the field lacks *in vitro* conditions that are reliably predictive of *in vivo* drug response. Therefore, we designed our *in vitro* models to cover a wide range of stressors encountered during infection with a goal of validating specific *in vitro* models for use in drug studies for Mtb. We therefore view the differences in drug interactions among conditions as an asset - drug action is dependent on the microenvironment in the animal and in these practical *in vitro* models. The accuracy of our predictors suggests that we can utilize suites of *in vitro* models to predict *in vivo* outcomes.

We have clarified these points in the main text:

> Lines 113-124: Mtb drug response is environment-specific, underscoring the need to validate *in vitro* models. For example, compared to standard (neutral) glucose-rich growth conditions, pyrazinamide is more effective in acidic *in vitro* conditions (Tarshis and Weed, 1953, McDermott and Tompsett, 1954) whereas bedaquiline is more effective in lipid-rich medium (Koul et al., 2014). Environment-specific drug efficacies are also observed *in vivo*; whereas treating C3HeB/FeJ mice with either pyrazinamide or bedaquiline results in both responding and non-responding populations of Mtb, treatment of BALB/c mice resulted in only responding populations. Drug response differences among mouse strains were attributed to lesion type and microenvironment differences and differential drug exposure in specific lesion compartments (Lanoix et al., 2016b, Irwin et al., 2016, Lanoix et al., 2016a, Lanoix et al., 2015b). The dependency of treatment efficacy on growth environment highlights the challenges in simplifying the complex *in vivo* environment into manageable *in vitro* growth conditions.
>
> Lines 255-258: Our findings are consistent with those from other organisms and adds to a growing body of literature that suggests synergy is a property of both drug and growth environment rather than an intrinsic property of drugs in combination (Belanger et al., 2020, Sanders et al., 2018, Dillon et al., 2019, Cokol et al., 2019).

4. Closely related to the previous point, all the higher-order drug combinations shown in Fig. 2B exhibit anything from strong antagonism to strong synergy depending on growth condition. This may also indicate that the definition used to classify higher-order drug interactions is inappropriate. It seems that only the single-drug responses were used as a reference for classifying the higher-order drug interactions (p. 9, lines 10-11). This is misleading since the drug interactions between all lower-order combinations of drugs involved should clearly be taken into account to define a sensible reference for a higher-order drug interaction. For example, if drugs A and B are strongly synergistic, synergy is also expected for any combination of A and B with a third drug if only the single-drug responses are used as a reference. Various ways to address this issue have been proposed (see e.g. PMID 22773816 or 27974577 and other work from the same groups). Any conclusions about the prevalence of synergy or antagonism (p. 10) and the lack of correlation of synergy with in-vivo efficacy (p. 10) remain doubtful unless these conceptual issues are properly resolved.

This is a very interesting point that we had not included in the first version of the paper. In previously published work, we have used DiaMOND to create a quantitative framework to

measure total drug interactions in high-order combinations and to measure the contribution of the total drug interaction (FIC_n) to emergent (εFIC_n) and lower-order (λFIC_{n-1}) components. This is similar in the emergent interaction framework from the Yeh lab noted by the reviewer. The total FIC (the metric we report in the manuscript) is the product of the emergent interaction and the geometric mean of the lower-order combinations. We have previously observed a mild correlation between both emergent interaction scores and lower-order interaction scores with the total drug interactions scores, as shown in figure S7 from Cokol et al. 2017 Sci Adv (10.1126/sciadv.1701881)):

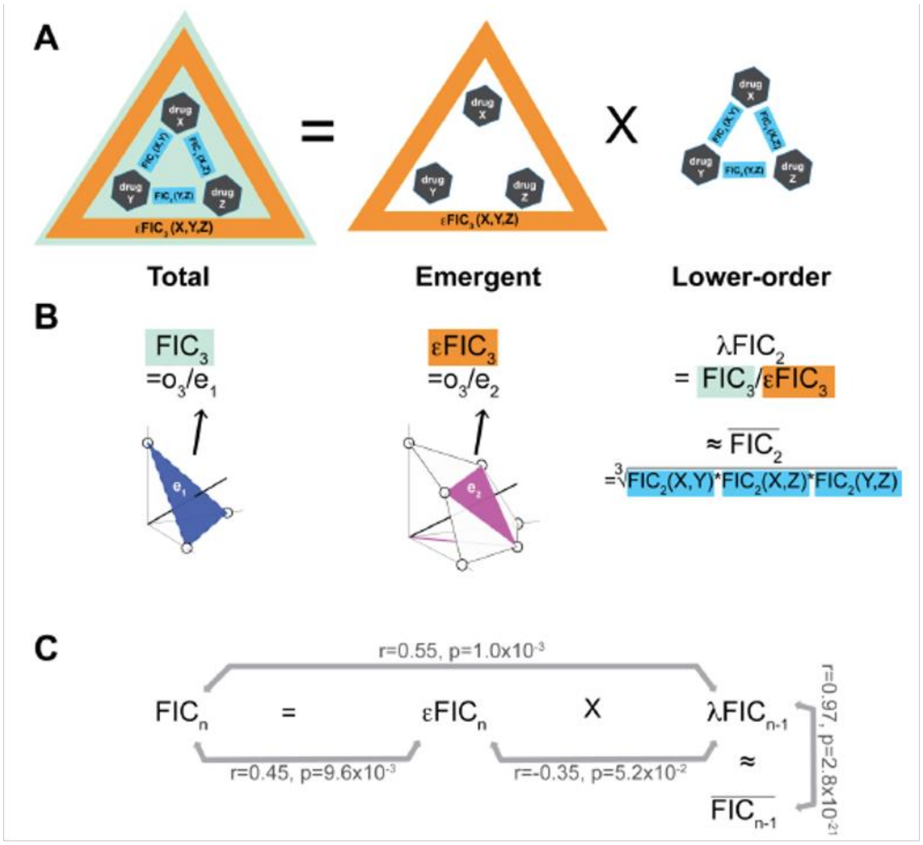

fig. S7. DiaMOND factorization model schematic. A to B) Visual representation (A) of the total, emergent, and lower-order interactions, measurements (A-B) and calculations (B) for a three-way interaction. Measured FICs are color-coded. The lower-order interaction score may be calculated from the total and emergent interaction scores, or it may be approximated by the geometric average of the component lower-order interactions, as shown. The measurement of FIC3, FIC3, all three FIC2 scores requires the measurement of only seven dose-responses (X, Y, Z, X+Y, X+Z, Y+Z, and X+Y+Z). For example, if X, Y and Z have IC50 at 10 units, while X+Y, X+Z and Y+Z have IC50 at 5 units, and then DiaMOND reports that all pairwise combinations are synergistic, with an FIC2 of 0.5 for each pairwise interaction. Imagine that for the same example, the combination dose response for X+Y+Z has an IC50 of 10 units. For the FIC3 calculation, the IC50 of X+Y+Z (10 units) would be compared to the plane defined by the IC50 of X, Y and Z (10 units), and because X+Y+Z's IC50 is same with additive expectation, we conclude an FIC3 score of 1. However, the calculation of eFIC3 requires the comparison of the IC50 of X+Y+Z (10 units)

with the plane defined by the IC50 of the X+Y, X+Z and Y+Z two-drug dose-responses (5 units). Because the three-way combination's IC50 is two times higher than the additive expectation from pairwise IC50's, DiaMOND concludes that eFIC3 of this combination is 2. Therefore, we find FIC3 = 1, and eFIC3 = 2, thus the three-way combination is additive, and it is more antagonistic than a sum of pairwise synergies because the pairwise interactions were overall synergistic. The contribution of pairwise interactions to FIC3 can be directly quantified by FIC3/eFIC3, by equation 4, we defined this quantity as lFIC2; for this example, FIC3/eFIC3 = 1 / 2 = 0.5, indicating that the overall contribution of pairwise interactions is synergy. lFIC2 can also be approximated by the geometric mean of all pairwise interactions, as used in equation 5 (shown in B above). In this example, FIC2 score for all pairwise interactions were 0.5, so their geometric mean is 0.5, perfectly approximating the lFIC2. C) Spearman correlation coefficients (r) and p values among the measured and calculated interaction scores in the factorization formula (equation 6).

Using this framework, we have broken down the factorization of the total drug interaction scores into emergent and lower-order contributions for the case shown in Figure 2B and also for the larger set of 3-drug combinations in the compendium (from Figure 2A). We demonstrate that prevalence of antagonism is a general phenomenon of both the emergent and lower-order interactions and that in the case of the combinations shown in Figure 2B, the emergent interactions are the stronger contributors to the overall effect than the lower-order components. Because the total interaction scores encapsulate both the lower-order and emergent contributions, we report the total interaction scores in this study. We have included the breakdown of the emergent and lower-order contributions to the total (overall) effect in Supp Figure S3B and briefly discuss emergent interactions in the results. In addition, Figure S2 demonstrates that the drug interaction scores are well correlated between the two major null models of Loewe additivity and Bliss independence for our dataset. We discuss this analysis in the revised manuscript:

> Lines: 269-278: To understand whether the drug interactions in high-order (3 or more drugs) combinations were due to lower-order interactions (for example, by pairwise synergies among the three component pairs of a three-way combination) or emergent properties from the high-order combination itself (Cokol et al., 2017, Beppler et al., 2016, Wood et al., 2012), we evaluated the contributions of lower-order and emergent interactions on the total interaction metrics (FICs, as shown in Figure 2A and B). The patterns in total and emergent drug interactions were similar while the lower-order drug interactions were generally more additive than the other two interaction types (Figure S3B). The strong emergent interaction scores indicate that the synergies and antagonisms we observed in total drug interactions are not systematically due to lower-order effects.

5. The fact that ignoring part of the data ('models') in the machine learning approach often leads to better performance (p. 14, top) is confusing. It indicates that the performance of the machine learning algorithms used may be limited (since they do not detect that it would be better to just ignore part of the data). A comparison to a straightforward linear regression (which should at least not suffer from this problem) would be helpful to clarify the value of the machine learning approach.

We apologize for the confusion. Only 67 of the 255 possible *in vitro* model combinations (26.3%) performed better than the classifier trained on all eight models. As shown in Figures 4A and S5G, individual *in vitro* models carry a different amount of signal that separates C0 and C1 data points, and it is not unreasonable to expect that the exclusion of *in vitro* models with poor signal can lead to improved overall performance.

> Lines 386-389: We systematically trained RF classifiers by considering all possible model combinations and observed that among the 255 possible combinations of *in vitro* models, 67 (26.3%) performed better than the classifier trained on all eight models (Data file S4).

The reviewer is correct that machine learning algorithms can automatically detect and exclude features with poor class separation, as is often done through model regularization. Indeed, one could impose a group LASSO penalty (http://dx.doi.org/10.1111/j.1467-9868.2005.00532.x) that would automatically exclude groups of features related to one or more *in vitro* models by setting the corresponding coefficients to zero. However, such an approach would produce a single solution only, while we were interested in systematically evaluating all possible combinations of *in vitro* models.

We thank the reviewer for suggesting to consider linear regression. We have reemphasized the use of linear models (PCA plots in Figures 3, 4, 5, S5, S6, S7) in our study. We note that individual principal components define linear combinations of the original features and marginal distributions presented as box plots provide a measure of performance for the corresponding models.

> Lines 132-134: The compendium contains information that can be linearly combined to distinguish drug combinations that outperform the standard of care.

> Lines 319-329: Principal component analysis (PCA) demonstrated that linear combinations of *in vitro* features could separate C0 and C1 drug combinations containing two and three drugs without using the *in vivo* class information (Figure 3A, Wilcoxon rank-sum, p<0.005, Data file S2). A separation was also apparent in PC spaces of combinations with two, three and four drugs (Figure S5A). Lastly, we also observed similar class separation when PCA was repeated using data from the constant and terminal time points separately (Figure S5B), indicating that similar conclusions would be drawn from any of the time point data. Because PCA does not use class labels in its computation, the observed separation of *in vivo* classes suggests that *in vitro* measurements from the DiaMOND compendium contain strong signal that characterizes the performance of drug combinations *in vivo*.

> Lines 336-339: To better separate C0 and C1 combinations based on the signals observed in PCA and make predictions for new combinations, we trained binary classifiers with eight different machine learning (ML) methods to distinguish C0 and C1 drug combinations and compared their performance in 5-fold cross-validation.

Lines 504-506: PCA on the compendium data alone was able to separate drug combinations that show treatment improvement in these mouse models.

Lines 551-554: We note that PCA and ML classifiers that included several *in vitro* models distinguish C1 and C0 classes better than when data from only one *in vitro* model was used, perhaps because the lesion microenvironment is complex and constitutes multiple stressors that affect Mtb drug response.

MINOR REVISIONS, SUGGESTIONS & COMMENTS

6. Fig. 3A: The fraction of variance explained by the PCs should be shown or mentioned in the figure legend. In particular, how high is it for the 27th PC? Is the clear discrimination for the two PCs shown robust (e.g. if PCA is performed on only part of the dataset or if some weak artificial noise is added to the data)?

We thank the reviewer for highlighting that the figures lacked the variance explained for each PC. We had originally chosen the PCs to display based on their abilities to separate C0 and C1 combinations, but agree that this is not ideal. We have performed a Horn's parallel analysis to determine how many PCs explain more variation than expected by chance. For both the RMM and BHeB outcomes, we find that the top 4 PCs describe significant variation and include this analysis in the results and as Figs S5F and S7A in this revision. We have updated the PCA plots (Figures 3, 4, 5, S5, S6, S7) to use two of the top four PCs and include the variance explained in the axis labels.

Lines 377-378 (for RMM): A Horn's Parallel Analysis (Figure S5F) identified the first four PCs as explaining more variance than expected by chance.

Lines 458-459 (for BHeB): A Horn's Parallel Analysis identified the first four PCs as explaining more variance than expected by chance (Figure S7A).

7. The representation of the drug interaction data in Fig. 2A is not ideal: It remains unclear which drug combinations are shown in the different columns. Since each drug is present in various different combinations, there should be a more systematic way of representing these data (e.g., for the 2-drug combinations, this is commonly done in a two-dimensional matrix, see e.g. Fig. 3 in PMID 16550172). It is clear that this is more difficult for higher-order interactions, but the authors should try to find a more informative way of showing these data than the current Fig. 2A.

We agree that it is ideal to mark each combination and that it is not possible to evaluate the drug interactions for individual combinations in our figure. In previous work we have used such a matrix to identify combinations. We have included below a version of Figure 2A in which we have added a matrix above the heatmap to identify drugs in each combination. However, with 165 combinations from 10 drugs, we believe that the annotated panel would be too dense and unwieldy for a flat figure. Instead, we include the dataset as Data file S1 and in Mendeley as a resource. We are open to working with the editorial staff to include this figure if the reviewers prefer it to our current representation.

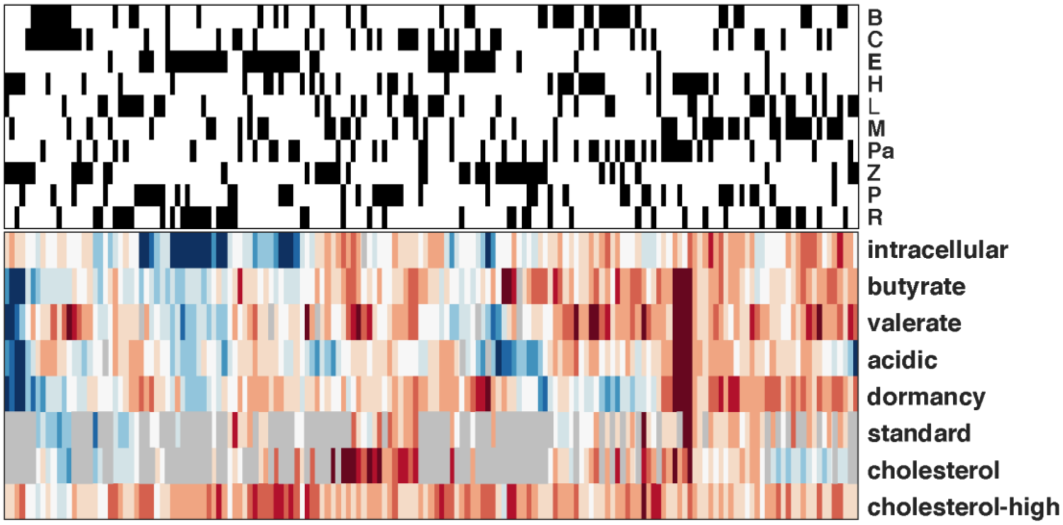

8. The color scale in Fig. 1A needs to be labeled with numbers (and possibly units). According to the figure legend, this color scale shows the IC90 but high IC90s would correspond to low potency, contradicting the label on the color bar. It needs to be clarified what is shown here.

We agree that this figure and legend were not clear. We have added the IC90 values onto the heatmap and clarified the units in the legend (now labeled as Figure 1B).

9. There is no doubt that the correlation in Fig. S2 is significant, but the p-value for the Pearson correlation (p. 9, line 17) is extremely small, which might be a result of strong assumptions underlying the statistical test used. The statistical test that was used to estimate this value should be mentioned here. A permutation test would be suitable here to estimate a more plausible p-value.

We have performed a permutation analysis and included these results in this revision and STAR Methods.

> Lines 231-233: Drug interaction metrics based on Loewe additivity and Bliss independence were correlated (FIC_{50} and FIC_{90} for the constant and terminal time points, r=0.81, p=$1.0x10^{-4}$, Pearson's correlation, using permutation analysis Figure S2).

> Lines 1228-1231: The hypothesis that Loewe and Bliss interaction (FIC) scores were correlated was tested using Pearson correlation, with the corresponding p-value computed empirically by randomly permuting values 20,000 times.

Reviewer #2: This exciting work addresses a fundamental challenge in improving therapies for tuberculosis, which rely on combination therapies. The paper builds on an approach called DiaMOND, developed by the Aldridge group in 2017, in which the universe of combination therapies is sampled in an efficient and optimized way to assess for synergy and antagonism. Here DIAMOND is applied to multiple *in vitro* growth conditions for M. tuberculosis that reflect the diverse microenvironments that M. tuberculosis encounters *in vivo* and then overlaid on fairly comprehensive compiled data from different animal models on efficacy of different regimens.

In the original work, pairwise and combination interactions were examined for nine drugs used against M. tuberculosis under standard *in vitro* growth conditions. But we know that during treatment of TB, one of the relevant factors is the range of microenvironments that we have to treat, and so the authors incorporate this into understanding optimal treatment regimens. Here, the authors expand on the proof-of-principle 2017 work, performing the modelling in 8 different *in vitro* models that capture the complex biology of TB. The DiaMOND method is highly efficient for reducing complexity and enabling assessment of drug combinations under defined conditions.

I really like the work - in concept, rigor, and extent. One of the major advances is the comprehensive integration of multiple *in vitro* conditions reflecting what we know about *in vivo* conditions into the prediction around a given regimen. One point, which the authors might be able to address (or articulate), is the added value of DiaMOND over predictions based on simpler models that would incorporate the 8 *in vitro* models without the array of drug combinations. The training and validation of course accounts for this and the output makes no assumptions about what will be important. But in other words, if one of the interesting conclusions that falls out is that potency is the key metric and that *antagonism* of combinations is correlated with efficacy, will it be more informative ultimately to develop, examine and measure fewer combinations under more and different *in vitro* conditions?

We thank the reviewer for their support and encouragement and for this constructive comment. We have reorganized and expanded the discussion to comment on these ideas. First, we discuss why making measurements in future work in more complex growth conditions may produce more predictive *in vitro* data. Second, we acknowledge that there is a lot more analysis that could be performed on this DiaMOND dataset - it is possible (and likely) that we can use fewer measurements to feed the classifier. We have discussed these ideas for future work in the discussion:

> Lines 594-608: Use of both acidic and lipid-rich *in vitro* models in developing classifiers is an example of how we were able to combine measurement in a “sum-of-parts” approach from relatively simple growth environments to model treatment outcomes despite the complexity of the microenvironments in TB lesions (Figure 4, Figure S5G). These results suggest that there is predictive drug combination response information obtained from simple *in vitro* models that only needs to be combined correctly to predict drug treatment outcomes in mice. The practical implication is that researchers can choose a subset of the most amenable *in vitro* models for performing drug combination experiments and still retain predictive capacity. Despite the success of summing together simple “parts” in predicting coarse outcome classifications, the importance of combination potency and variation of potency among growth conditions raises the question of whether measurement in more complex growth environments (e.g., reflecting multiple aspects of the host microenvironment) would further refine the classifications and improve prediction accuracy. The richness of this DiaMOND dataset will also enable future studies to understand whether *in vivo* combination outcomes can be predicted using few *in vitro* data, for example by using lower-order combination data alone.

> Lines 667-679: We expect this pipeline to improve as the component drug set is expanded and diversified via new drug discovery methods (Hie et al., 2020, Johnson et al., 2019, Rock, 2019, Rock et al., 2017, VanderVen et al., 2015, Bryk et al., 2008, Gold and Nathan, 2017, Gold et al., 2015) and as more animal studies provide outcome data for drug combinations with new combinations and drugs. We also note that the size of drug combination classification sets used in this study was achieved by compiling and giving equal weight to

diverse animal studies from multiple investigators with differences in study designs (e.g. infection protocols, drug treatment times, Mtb strains, etc.). Developing and validating across-study standardizations may improve future analysis, modeling, and predictions. Mtb drug response and virulence can be strain dependent (De Groote et al., 2012, Bigelow et al., 2020) and the strain of Mtb used in this study (Erdman) differed from those used in some of the animal studies (e.g. H37Rv). In future work, including multiple Mtb strains (including clinical isolates) may improve combination classification separation and model predictions.

The mouse outcome data across models is a fantastic compilation of studies and is used to great effect. I do think there should be some discussion of any caveats that might come from 1) combining and giving equal weight to diverse animal studies from different investigators; and 2) using the binary metric of C0 and C1 for outcome. I don't have any problem with the approach, but the authors should consider or mention any caveats.

We thank the reviewer for this feedback. We have made changes in the revision to discuss both points in more detail in:

Lines 313-319: Most studies included a standard of care treatment making these annotations straightforward. For studies where no standard of care treatment was included, we inferred annotation by comparing to a study that shared at least one other drug combination treatment and that also included standard of care. This annotation limits the resolution of treatment improvement we can evaluate, but does not necessitate normalization between studies (e.g. infection inoculum, drug treatment time, or Mtb strain) that might be needed for more quantitative assessment of treatment improvement.

Lines 671-675: We also note that the size of drug combination classification sets used in this study was achieved by compiling and giving equal weight to diverse animal studies from multiple investigators with differences in study designs (e.g. infection protocols, drug treatment times, Mtb strains, etc.). Developing and validating across-study standardizations may improve future analysis, modeling, and predictions.

There were honestly no technical issues at all that I could find - the work is superb.

We are grateful for this kind comment - thank you!

The authors state in passing in the results section that they developed a low-oxygen multi-stress model based on the inclusion of a nitrate source for respiration in addition to butyrate as a carbon source and hypoxia. Since this is the first description of these particular conditions in the literature and is one of the conditions that they use it might be nice to expand a little on this somewhere.

We thank the reviewer for this suggestion. We have expanded the rationale and discussion of this condition in the results:

Lines 166-173: Mtb in a non-replicative state are particularly challenging to sterilize (Sarathy et al., 2018). Many models of non-replicative state have been developed for laboratory study that involve single or multiple stresses (Deb et al., 2009, Early et al., 2019, Del Portillo et al., 2018, Parish, 2020, Gold et al., 2015). Based on some of these previously published models, we chose to develop a model that would be amenable to large-scale, multi-well experiments with drug combinations. Our dormancy model is a low-oxygen

multi-stress model that induces dormancy using butyrate as a carbon source, sodium nitrate to respire (Cunningham-Bussel et al., 2013a, Cunningham-Bussel et al., 2013b, Sohaskey, 2008), and plate seals to limit oxygen (dormancy).

Some of the references appear to be unlisted anywhere (eg Li et al 2015, Li et al 2017). Should double check.

We thank the reviewer for noticing this important oversight. We have added the missing citations to the references and notes along with others that were omitted during reference compilation.

One stylistic point, which is totally up to the authors. Some of the metaphorical jargon, particularly when combined in close succession, can be distracting - pipelines, landscapes, maps, paths - and might be used judiciously.

Guilty as charged! We have revised the manuscript to reduce the jargon.

Reviewer #3: This well-written manuscript describes a very ambitious study in which the authors systematically measured the Mycobacterium tuberculosis response to every 2- and 3-drug combination of 10 drugs in up to 8 different *in vitro* conditions intended to simulate key aspects of the various microenvironments and lesion compartments encountered by the pathogen *in vivo*, then used machine learning to develop classifiers predictive of combination treatment outcomes in previously published mouse model studies. The classifiers were validated using higher order (4- and 5-drug) combinations and combinations including several drugs not included in the training set.

A number of important observations are made. Firstly, the model using cholesterol as a carbon source appeared to be the single *in vitro* model most predictive of results in mice, but signatures including several *in vitro* models performed better than signatures using data from any one *in vitro* model. Moreover, predictors including only the technically simpler *in vitro* models performed as well or better than those including more complex *in vitro* models (ie, intracellular infection, hypoxic stress, high cholesterol concentration). This observation may pave the way to using multiple simpler *in vitro* models together to give a composite ("sum-of-parts") picture of combination performance across several conditions and thereby close a major gap in TB drug and regimen development by enabling confident prediction of *in vivo* efficacy from *in vitro* models. Secondly, drug potency measures were more important predictors of combination drug outcomes than interaction measures, suggesting that potent drug combinations should be prioritized for further study and should not necessarily be deprioritized if they are antagonistic in *in vitro* assays. There are also surprising results that will require further study to confirm and explain. For example, *in vitro* models had less predictive capacity for predicting *in vivo* outcomes based on the change in lung CFU counts in non-C3HeB/FeJ mouse strains (BMM outcomes) compared to outcomes based on relapse in non-C3HeB/FeJ mouse strains (RMM outcomes) and change in CFU in C3HeB/FeJ mice (BHeB outcomes). Other interesting observations stemming from the *in vitro* studies themselves include that synergy appears to be a property of both drug and growth environment rather than an intrinsic property of drugs.

Overall, the manuscript and the work described is a tour de force that is expected to have important and lasting impact on the TB drug and regimen development space. Some additional major and minor comments are provided for the authors' consideration below.

Major comments:
The observation that multiple *in vitro* models may combine to give a "sum-of-parts" picture of *in vivo* efficacy is important. Even in less complex (i.e., non-C3HeB/FeJ) mouse models, some bacilli reside in acidified phagolysosomes (for which the acidic growth model may be most relevant), while others reside in foamy macrophages (for which cholesterol and lipid carbon source models may be most relevant). This point that even the simpler *in vitro* models identified as most informative may be representing specific *in vivo* niches and bacterial sub-populations is implied in the Discussion but could be made a bit more explicitly. The similar predictive power of the best classifiers between the RMM and BHeB outcomes also suggests that the key microenvironments may be more similar in these mouse models than is currently appreciated.

We thank the reviewer for this suggestion. We have expanded areas of the results and discussion to make these points more explicit.

> Lines 585-593: However, like the BALB/c mice which only form lesions with intracellular Mtb, these animals have abundant intracellular bacteria in other lesion types and within macrophages that acidify the intracellular Mtb compartments (Irwin et al., 2016) which may explain why acidic growth environments are important predictors of drug response in this mouse model. Furthermore, Mtb residency in lipid-laden, foamy macrophages is important in both the BALB/c (and similar mouse strains; (Shim et al., 2020, Zhang et al., 2011, Rosenthal et al., 2012, Driver et al., 2012) as well as C3HeB/FeJ mice (Driver et al., 2012, Rosenthal et al., 2012), supporting the idea that *in vitro* lipid growth conditions provide important information about *in vivo* drug response for both the RMM and BHeB model.

> Lines 594-608: Use of both acidic and lipid-rich *in vitro* models in developing classifiers is an example of how we were able to combine measurement in a “sum-of-parts” approach from relatively simple growth environments to model treatment outcomes despite the complexity of the microenvironments in TB lesions (Figure 4, Figure S5G). These results suggest that there is predictive drug combination response information obtained from simple *in vitro* models that only needs to be combined correctly to predict drug treatment outcomes in mice. The practical implication is that researchers can choose a subset of the most amenable *in vitro* models for performing drug combination experiments and still retain predictive capacity. Despite the success of summing together simple “parts” in predicting coarse outcome classifications, the importance of combination potency and variation of potency among growth conditions raises the question of whether measurement in more complex growth environments (e.g., reflecting multiple aspects of the host microenvironment) would further refine the classifications and improve prediction accuracy. The richness of this DiaMOND dataset will also enable future studies to understand whether *in vivo* combination outcomes can be predicted using few *in vitro* data, for example by using lower-order combination data alone.

There is limited diversity in the regimens tested in mice by virtue of relying on published data. Nearly all combinations (e.g., all but 2 3-drug combinations) include either a rifamycin or bedaquiline, and often pyrazinamide. This is an important caveat when assessing the value of drug interaction features for classification. If the efficacy of rifamycin-based and bedaquiline-based regimens is highly dependent on the efficacy of those single drug components (or the combination with pyrazinamide), the observation about the limited predictive impact of drug interaction features may not be as generalizable as one may assume. This may be compounded by the challenges inherent in modeling the *in vivo* effects of pyrazinamide in many *in vitro* conditions.

The high performance of the best-performing classifiers trained on the simple *in vitro* models on the validation set of 4- and 5-drug combinations and additional drugs is promising but it should be acknowledged that 2 of 3 new drugs added in this validation set (delamanid and sutezolid) are from the same drug class and have the same mechanism of action as drugs in the training set. Another limitation is that the efficacy of the higher order combinations is often driven by the efficacy of lower order combinations.

We thank the reviewer for these excellent points, which are similar to those also raised by Reviewer #1. We have expanded the introduction and discussion to describe the iterative TB regimen design process that adds to successful drug combinations and to acknowledge overlap in drugs between the training and test sets. We have also included new analysis that demonstrates that the predictor is dependent on overlap of drugs between the training and test sets, but that leaving one drug out at a time (e.g. to test combinations involving a new drug) produced reasonable accurate classifiers for the majority (but not all) of the drugs in this study. We have updated our results and discussion to directly address the limitation of the modeling and to better describe how *in vitro* measurement should fit into the drug regimen design pipeline. Please refer to our response to Reviewer #1's major comment 2 for a detailed explanation of our new analysis and revision of the manuscript. To address this comment directly, we have also acknowledged the overlap in drug type between the validation and training set in the results:

> Lines 395-397: SQ109 represents a drug in a new antibiotic class, while delamanid and sutezolid are in the same antibiotic classes as drugs in the 10-drug set (pretomanid and linezolid, respectively).

In addition, we have evaluated the relative contributions of emergent and lower-order effects on the drug interactions of high-order combinations. As described in detail in our response to Reviewer #1's major comment 4, we found that both the lower-order and emergent interactions contributed to the total interaction scores, but the total interaction scores are primarily driven by emergent behaviors of the high-order combinations.

The analyses focused on 1-2 time points per *in vitro* model, although more time points were assessed. FigureS3 seems to suggest that results using the terminal time point vs. the constant time point may be more different than one would expect. Were any sensitivity analyses considered or performed to determine whether use of different time points would have altered any results or conclusions?

This is a very good question that we should have addressed more directly in the paper. Due to the different time scales of the measurements made in each *in vitro* model, we do not have identical time points across the conditions. For this reason, we chose the constant (across all time points) and last time point (for each condition) so that we could interpret the data at the relatively early (constant) and late (terminal) times. To understand whether our major conclusions were dependent on the time points we evaluated whether class separation in the DiaMOND data was detected at one time point and not the other, we used PCA to evaluate class separation at the constant and terminal time points, in separate analyses. Because these two time points span the early to late duration of the experiments (and there is no rational way to combine intermediate time points among the conditions), we evaluated the constant and terminal time points only. Because class separation is observed at both of these time points and other time points (intermediate) exhibit behaviors between those in the constant and terminal times, we conclude

that use of other (intermediate) time points would be unlikely to change the outcome of these analyses.

We have included these analyses as FigureS5B and discuss it in the results.

> Lines 323-326: Lastly, we also observed similar class separation when PCA was repeated using data from the constant and terminal time points separately (Figure S5B), indicating that similar conclusions would be drawn from any of the time point data.

It should be acknowledged in the discussion that the bacterial strain used in the *in vitro* studies was not the same strain used in many of the mouse studies. This is not necessarily a weakness but it is a potential limitation that may not be evident to many readers.

This is an excellent point. We have added this point to the discussion.

> Lines 676-679: Mtb drug response and virulence can be strain dependent (De Groote et al., 2012, Bigelow et al., 2020) and the strain of Mtb used in this study (Erdman) differed from those used in some of the animal studies (e.g. H37Rv). In future work, including multiple Mtb strains (including clinical isolates) may improve combination classification separation and model predictions.

Was there any concern for selection of drug-resistant mutants over 7 days in the active growth model(s) that might confound the drug interaction measures?

This is another excellent point. We expect that if drug resistance development were an issue, we would observe static growth at earlier time points followed by increased growth at later time points. This would likely be most extreme in the single drug treatments where selection for resistance would only be needed against a single agent. We did not observe this pattern and therefore we believe that any drug resistance that might have developed during treatment did not influence drug interaction measures. We have added a discussion about possibility of drug resistance and how impact on acquisition of drug resistance should be considered in future drug combination studies:

> Lines 659-665: Though this study was not designed to test the impact of combination therapy on development of drug resistance, future studies could adapt the DiaMOND experiments to assess drug resistance using luminescence reporters(Zhang et al., 2012a). We did not directly evaluate drug resistance in our assays, but we did not observe outgrowth over time at high drug concentrations in the longer drug treatments, suggesting that any resistance development that occurred did not overtake the populations and influence dose response measurements.

Minor comments:

Errors:

> Page 4, line 1 (and elsewhere): suggest "treatment-shortening" rather than "treatment-shorting"

Page 4, last line: suggest "...including granulomas..."

Page 30, paragraph 3, line 3: "To compare low dose potency..."

Page 30, second-to-last line: "We log transform FICs..."

Fig 1 legend: "C" is missing on the figure; "B" should be next to the curve below the cube rather than next to the cube

Fig S5C: cholesterol is missing an "l" at the end in the right y axis.

Figs S8 and S9: there are asterisks in the figure legends but none in the graphs themselves.

Table S2 legend, line 2: combination should be singular, not plural?

Table S7: shouldn't HRpZE be classified as "C1" here, as discussed in the text?

We have corrected these errors.

Page 5, line 3: model-specific drug responses may also be attributable to different lesion structures leading to differential drug exposures in specific lesion compartments in addition to different microenvironments

This is very true. We have added this information:

> Lines 94-99: Mtb drug response differs between these two types of mouse models, and both are important preclinical tools because the model-specific drug response is thought to result from the different lesion microenvironments present in each animal model (Figure 1A) and the differential drug exposure in lesion compartments is influenced by lesion structure (Driver et al., 2012, Lanoix et al., 2015b, Lenaerts et al., 2015, Nuermberger, 2017).

Page 7, paragraph 2: The mechanism of action of clofazimine may not be well described by inhibition of energy metabolism or respiratory inhibitor (as in Table S2). What is the basis for this classification?

The pathway of action of clofazimine is complicated. For consistency, we have updated our table to say it is an "antimycobacterial/multi-process inhibitor". Our initial characterization was based on Cholo et al 2012 who described clofazimine activity at the membrane as leading to disruption of ATP synthesis. In addition, Yano et al 2011 showed that clofazimine is a respiratory chain inhibitor by generating ROS as a likely cause of cell death.

Page 9, 6th line from bottom: the rationale for selecting Loewe additivity over Bliss independence as the null model for systematic analysis in the compendium should be provided for the reader.

We have clarified the reasoning of this choice in the results:

> Lines 231-236: Drug interaction metrics based on Loewe additivity and Bliss independence were correlated (FIC50 and FIC90 for the constant and terminal time points, r=0.81, $p=1.0\times10^{-4}$, Pearson's correlation, using permutation analysis Figure S2). In our analysis, we

proceeded with Loewe additivity as the null model in our drug interaction (FIC50 and FIC90) calculations because we have previously validated additivity measures using sham combinations using Loewe additivity (Cokol et al., 2017).

Page 23, last 10 lines: discussion of Study 31 and the PHZE prediction should also include discussion of the PHZM prediction from the DiaMOND compendium. PHZM is the first trial-confirmed 4-month regimen non-inferior to RHZE. What was the consensus prediction for PHZM from the *in vitro* compendium and how did that align with RMM and clinical outcomes? This section paints the predictions of RMM and DiaMOND as dichotomous (ie, right or wrong) but in reality treatment response is continuous. The rank order of PZM > PZH > RZM > RZH in the RMM was also consistent with rank ordering from Study 31 and REMox (ie, PZMH > PZHE > RZM (H/E) > RZHE, although only PZMH was the only regimen to meet non-inferiority criteria as a 4-month regimen. Therefore, the DiaMOND compendium results may be more consistent with mouse outcomes and not "contradictory", as implied here.

We thank the reviewer for highlighting the missing evaluation of the treatment improving combination. Our validation dataset did not include the PHZM (because it had not been evaluated in mouse models). Because we cannot evaluate whether the consensus predictions are consistent with the clinical outcomes for all three treatment arms (PHZM > HRZE/PHZE), we have limited the discussion relating predictions to clinical outcomes to the REMox trial where we have all the combinations tested *in vitro*. Future *in vitro* studies will certainly include the “Study 31” combinations so that model comparison with clinical outcomes can be evaluated.

Fig S4: why does the vertical order of the models change across the graphs? It makes it more difficult to compare results across the graphs. Is there a rationale for doing this?

We apologize for the confusion. We clustered both by drug combination and *in vitro* model to determine if the relationship between the *in vitro* models was time dependent. We have explained this reasoning in the results.

> Lines 301-306: We also observed different relationships between *in vitro* models when either metrics or separate time points were investigated (Figure S4C and D), suggesting that metrics of potency across timescales provide non-redundant information about drug combinations. The correct ordering of selected drug combinations by mouse outcome suggests that the DiaMOND compendium contains useful information for identifying efficacious drug combinations.

Table S2: it's likely not appropriate to pigeonhole pyrazinamide as a respiration inhibitor based on studies by Thomas Dick and colleagues, nor SQ109 as strictly a cell wall synthesis inhibitor based on evidence for its effects on membrane potential and respiration.

We agree with the reviewer. We have revised the table to more appropriately attribute the pathway of actions to reflect the general mycobacterial activity of the drugs and the multiple affected pathways given the current state of the literature.
